# Remote Migratory Reductive Arylation of Unactivated Alkenes Enabled by Electrochemical Nickel Catalysis

**DOI:** 10.1002/cssc.202402196

**Published:** 2024-11-19

**Authors:** Chao Xu, Ru‐Han A, Xiao‐Feng Wu

**Affiliations:** ^1^ Dalian National Laboratory for Clean Energy Dalian Institute of Chemical Physics Chinese Academy of Sciences Dalian China; ^2^ University of Chinese Academy of Sciences Beijing China; ^3^ Leibniz-Institut für Katalyse e. V. Rostock Germany

**Keywords:** Nickel catalyst, Cross-coupling, Alkene, Aryl bromide, Electrochemistry

## Abstract

Transition metal‐catalyzed cross‐coupling reaction between organometallic reagents and electrophiles is a potent method for constructing C(*sp* ^*2*^)−C(*sp* ^*3*^) bonds. Given the characters of organometallic reagents, cross‐reductive coupling is emerging as an alternative strategy. The resurgence of electrochemistry offers an ideal method for electrochemical reductive of cross‐coupling electrophiles. Inspired by the mechanism of electrochemical metal hydride, our study proposed that Ni−H electrochemically catalyze the hydroarylation coupling of unactivated alkenes with aryl halides. 1,1‐Diarylalkanes can be produced effectively. This method have advantages including mild conditions, excellent regioselectivity, and satisfactory yields.

## Graphical abstract

we have developed an electrochemical Ni−H catalyzed arylation coupling method of unactivated alkenes with aryl halides. The method displays broad functional group tolerance and proceeds under very mild conditions. Furthermore, aryl chlorides were also compatible substrates in this catalytic system. This conversion holds significant implications for the construction of 1,1‐diarylalkanes.

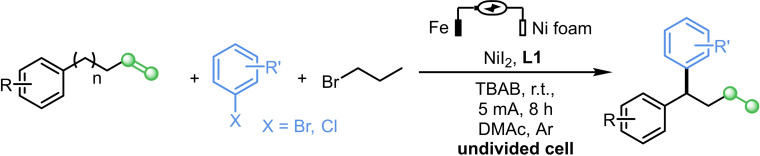

## Introduction

**Scheme 1:**
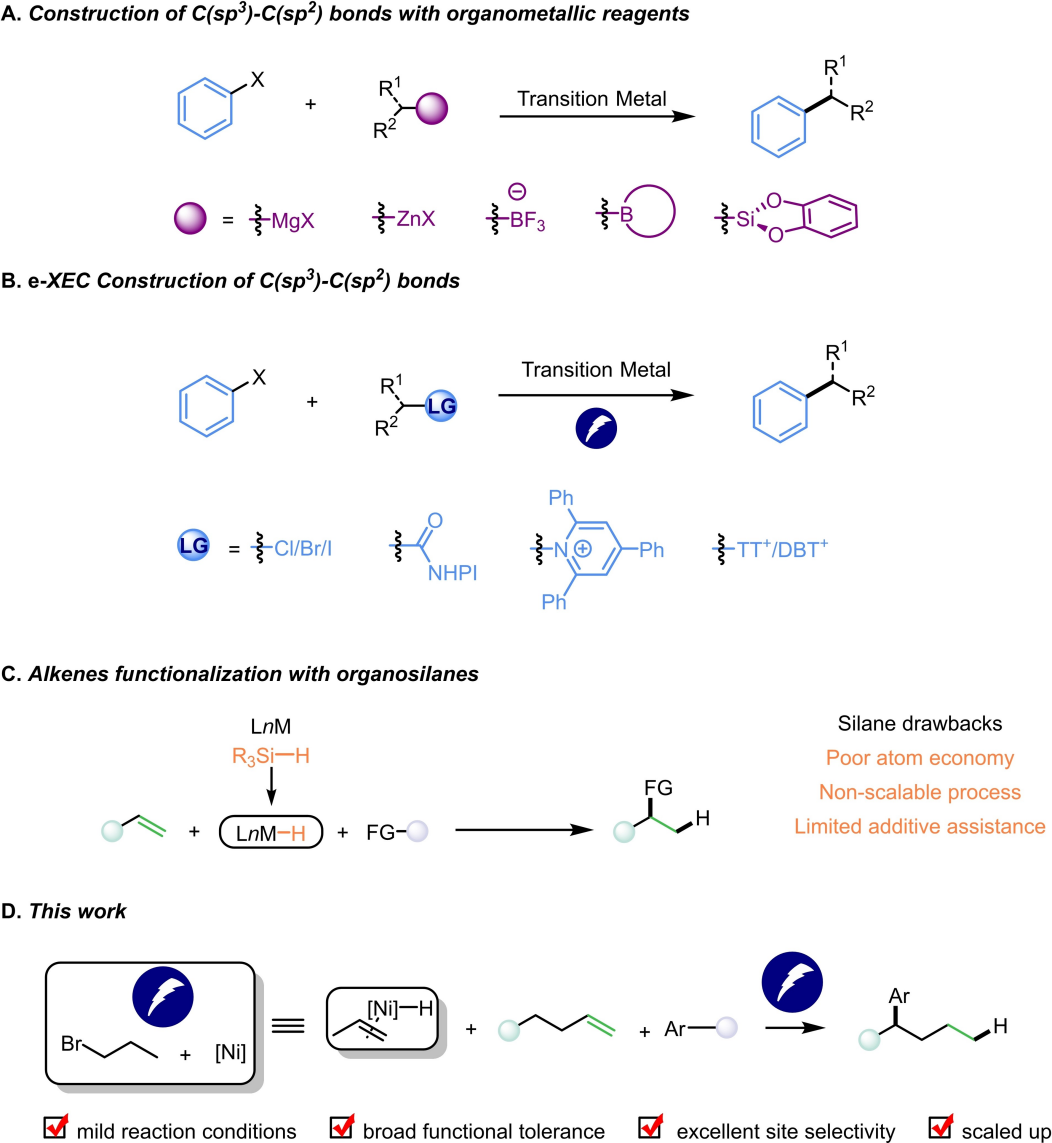
Cross‐coupling reactions and reductive cross‐coupling reactions.

The transition metal‐catalyzed cross‐coupling reactions between organometallic reagents and organic electrophiles have emerged as powerful synthetic tools for forging C(*sp* ^*2*^)−C(*sp* ^*3*^) bonds (Scheme 1A).^[1]^ From the perspective of manipulation step‐ and atomic economy, the requirement of pre‐generated organometallic reagents are problematic sometimes. Recently, substantial efforts have been spent on the development of transition metal‐catalyzed reductive cross‐coupling (XEC) reactions.[^4^, ^5^, ^6^, ^7^, ^8^] Catalysis utilizing earth‐abundant nickel has emerged as a practical tool for carbon−carbon bond formation, standing out among the metal‐catalyzed cross reductive couplings. Inspired by Weix and co‐workers’ seminal work,^[9]^ many nickel‐catalyzed reductive cross‐coupling strategies for constructing C(*sp* ^*2*^)‐C(*sp* ^*3*^) bonds have been developed.^[10]^ And the cross‐coupling of two electrophiles catalyzed by transition metals has emerged as a general, efficient strategy for building C−C bonds. However, these reactions require a metal reductant (typically Mn or Zn) to regenerate the nickel catalyst. Hence, achieving the desired reaction via more environmentally friendly and economical methods remains highly desired but challenge.

Over the past few decades, electrochemical strategies have received significant attention in various organic research fields,^[13]^ and they generally play two crucial roles in organic transformation: oxidation or reduction. The resurgence of organic electrocatalysis is a promising approach for solving synthesis challenges that cannot be effectively addressed by existing methods.

Recently, many strategies for constructing C(*sp* ^*2*^)‐C(*sp* ^*3*^) by electrochemical nickel catalytic reduction have been reported (Scheme 1B).^[22]^ The mild functional group transformation of C(*sp* ^*3*^)−H bonds in the remote position of the alkyl chain often requires the involvement of directing groups, while nickel‐catalyzed reactions often suffer from isomerization mediated by β‐H elimination, which is usually an undesirable process in coupling reactions but is considered to be an effective means of selective C(*sp* ^*3*^)‐H arylation.^[28]^ Electrochemical reduction nickel‐catalyzed chain walking electrophilic cross‐coupling has been reported to achieve alkyl‐chain benzyl arylation under mild electrochemical conditions.^[32]^ From synthetic point of view, as alternative strategy, unactivated alkenes with higher abundance are undoubtedly attractive starting materials to construct C(*sp* ^*2*^)−C(*sp* ^*3*^) bonds.

A significant character of alkenes is that they are not only readily accessible but also possess high reactivity and with various transformation potential. Unlike the conversion of alkenes by metal hydrides with the participation of organosilane (Scheme 1C),^[34]^ the electrochemical reaction involving metal hydrides was less studied.^[37]^ Recently, Qiu and co‐workers revealed that Ni−H catalyzes the C(*sp* ^*3*^)‐C(*sp* ^*3*^) electrochemical coupling between alkyl halides and aliphatic alkenes.^[42]^ In this protocol, alkyl bromine not only served as a coupling partner but also generated Ni−H as the active intermediates. The arylation of activated alkenes catalyzed by electrochemical Ni−H has been mentioned,^[33]^ however, the remote functional group of unactivated alkenes catalyzed by electrochemical Ni−H still requires further investigation. To explore a sustainable approach for electrochemical C(*sp* ^*2*^)−C(*sp* ^*3*^) bond formation, based on our research group's interest in the functional group conversion involving metal hydrides and unactivated alkenes,^[43]^ we hereby present an electrochemical nickel catalyzed arylation coupling method of unactivated alkenes with aryl halides (Scheme 1D). This conversion holds significant implications for the construction of 1,1‐diarylalkanes, a common structural motif found in many natural products and bioactive compounds.^[46]^ This protocol proceeded smoothly under electrochemically mild conditions. Alkyl halides acted as the hydrogen source, obviating the addition of silane. Without reducing metal reagents, various functional groups showed excellent tolerance and yields even in gram scale. This work also exhibited excellent chem‐ and regioselectivity. Besides aryl bromides, aryl chlorides can be effectively tranformed as well.

## Results and Discussion

### Reaction Optimization

The investigation started with the model reaction of electrolyzing **1** with **2** in DMAc. With Ni(ClO₄)_2_ ⋅ 6H₂O (10 mol %) and **L1** as the catalyst, 1‐bromopropane (1.5 equiv.) as the hydrogen source, and tetrabutylammonium bromide (TBAB) as the supporting electrolyte at a constant current (4 mA) in an undivided cell. The desired coupling product **3** can be obtained in 78 % yield with excellent regioselectivity in the presence of 1.5 equivalents of alkene and 1.0 equivalent of aryl bromide (Table 1, entry 1 and Table S1). Alternating solvents had an adverse effect on the reaction outcome (Table S2). NMP can provided similar yield (Table S2, 77 %), but was not further studied due to separation issues. Various nickel salts were evaluated (Table S3, entry 1–10), and NiI_2_ provided the highest yield (Table 1, entry 3, 90 %). It was worth noting that the methyl groups on the ligands **L1**, **L2**, **L3** (Table 1, entries 3–5) were crucial for the reaction. Alternating electrodes had an adverse effect on the reaction results (Table S5). Finally, the effect of current was investigated, and the optimal yield was obtained at 5 mA for 8 hours (92 %) with 5.0 F/mol (Table 1, entry 8). Subsequently, different alkyl bromide compounds were tested and yields of 34–79 % were obtained (Table S7). A control experiment revealed that no coupling product was produced in the absence of the catalyst or ligand (Table 1, entry 10).

**Table 1:** Optimization studies.^[a]^

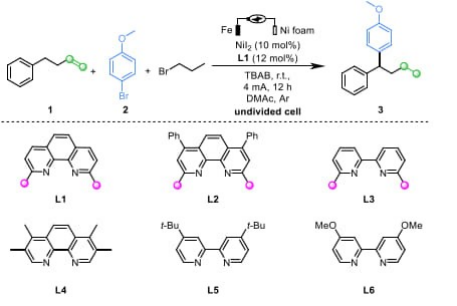			
Entry	Deviation from above	F/mol	Yield (%)
1	Ni(ClO_4_)_2_⋅6H_2_O	6.0	78
2	DMF/MeCN	6.0	22/11
3	None	6.0	90
4	L2	6.0	84
5	L3	6.0	53
6	L4 ‐ L6	6.0	trace
7	4 mA (10 h)	5.0	92
*8*	*5 mA (8 h)*	*5.0*	*92 (84* ^[b]^ *)*
9	6 mA (6 h)	4.5	82
10	other Alkyl bromides	5.0	34–79
11	w/o Ni or Ligand	5.0	0

[a] Reaction conditions: **1** (0.45 mmol), **2** (0.3 mmol), 1‐bromopropane (1.5 eq.), NiI_2_ (10 mol %), **L1** (12 mol %), TBAB (0.3 mmol), DMAc (4.0 mL), in an undivided cell, Fe as anode, Ni Foam as cathode, constant current=4 mA, r.t., 12 h, under Ar atmosphere. Yields were determined by GC with dodecane as an internal standard. [b] Isolated yield.

### Substrate Scope

**Scheme 2:**
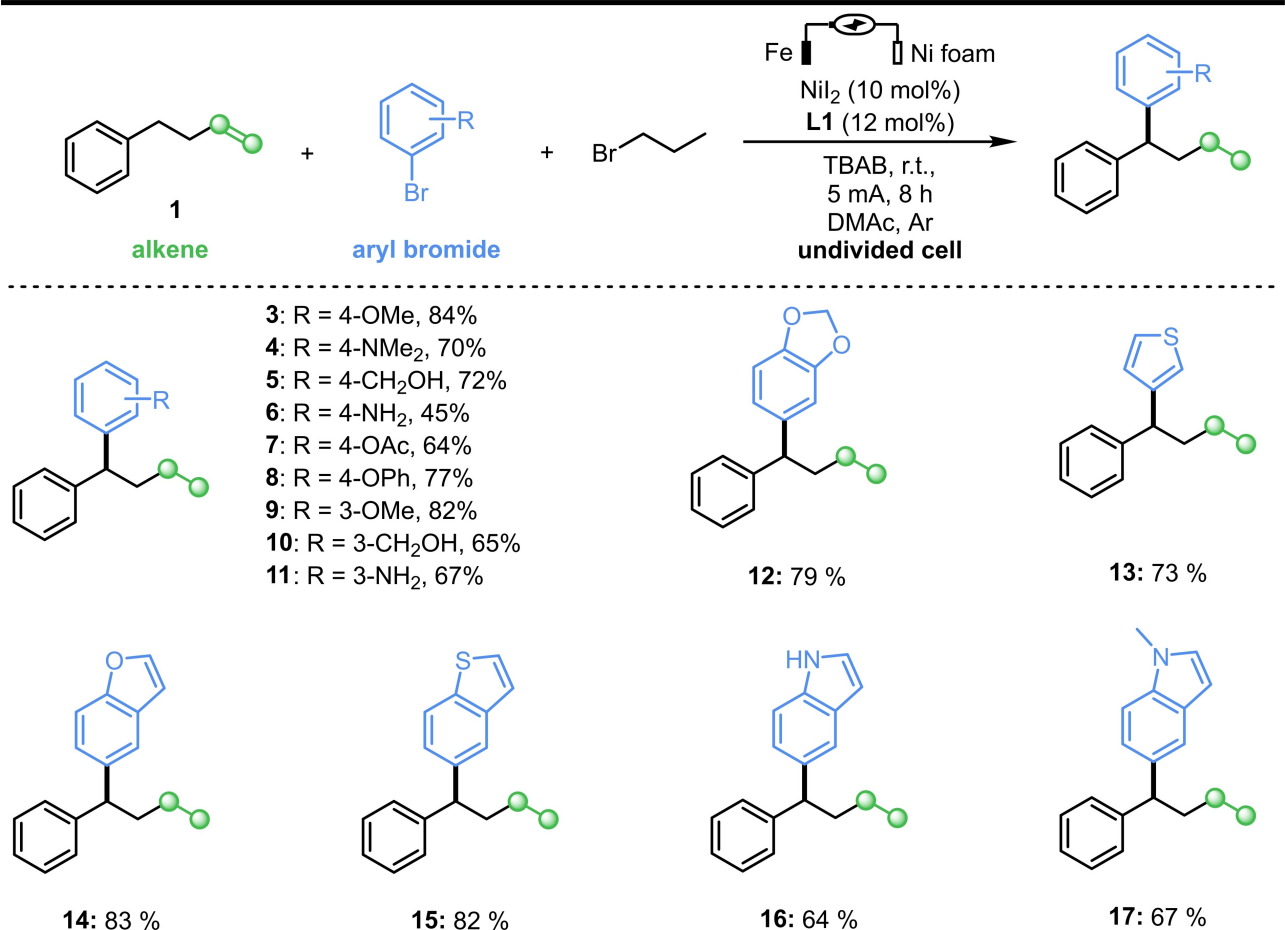
Substrates scope of aryl bromides.

**Scheme 3:**
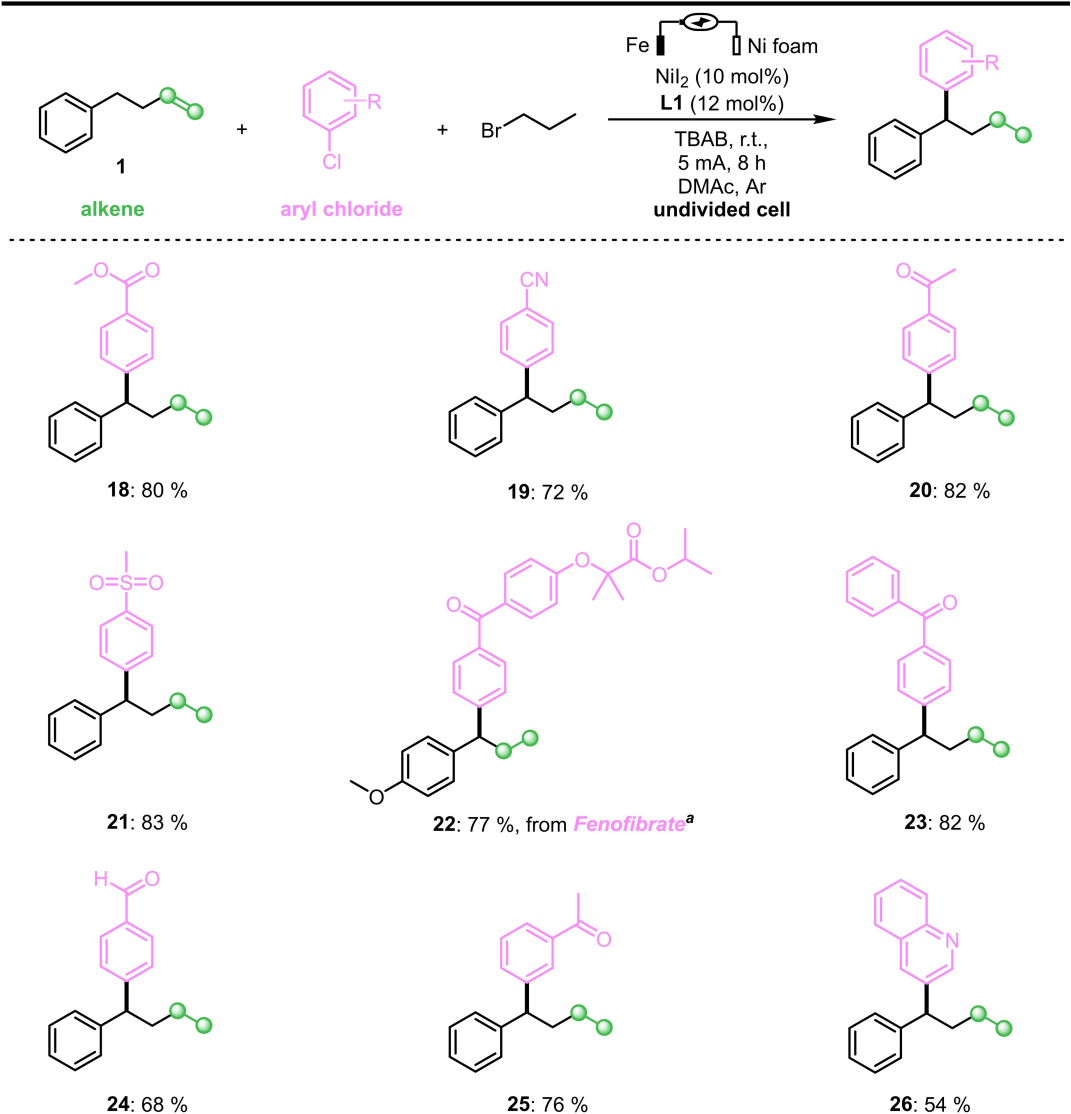
Substrates scope of aryl chlorides. ^[a]^1‐Allyl‐4‐Methoxybenzene as alkene.

With the optimized conditions in hand, the generality of this new protocol was investigated. A series of aryl bromides was evaluated (Scheme 2), affording medium to good yields (**3**–**17**). For substrates containing more active groups such as hydroxyl, amino or protected amino groups (**4**–**6**), the electrochemical protocol could be used to produce the corresponding products. It should be noted that, compared with **6** and **11**, 2‐bromoaniline could not obtain corresponding product. Delightedly, when we investigated the corresponding aryl chlorides, the remote migratory reductive coupling reaction proceeded well with good to excellent yields (Scheme 3). Substrates with ester, nitrile, ketone, methyl sulfonyl, or aldehyde groups in the reaction were well tolerated, affording good yields. This compensates for the defect that aryl bromide substituted by electron‐withdrawing groups is more prone to generate dimer products. Subsequently, heterocyclic rings were considered, and the corresponding products could be obtained from quinoline ring substrates in medium yield (**26**). In addition, we attempted to modify drug molecules using this electrochemical protocol. Fenofibrate, a commercialized drug for treating cardiovascular disease, worked well under the protocol (**22**). Electron‐rich aryl chlorines such as chloranisole have also been investigated, but no corresponding coupling products were obtained.

**Scheme 4:**
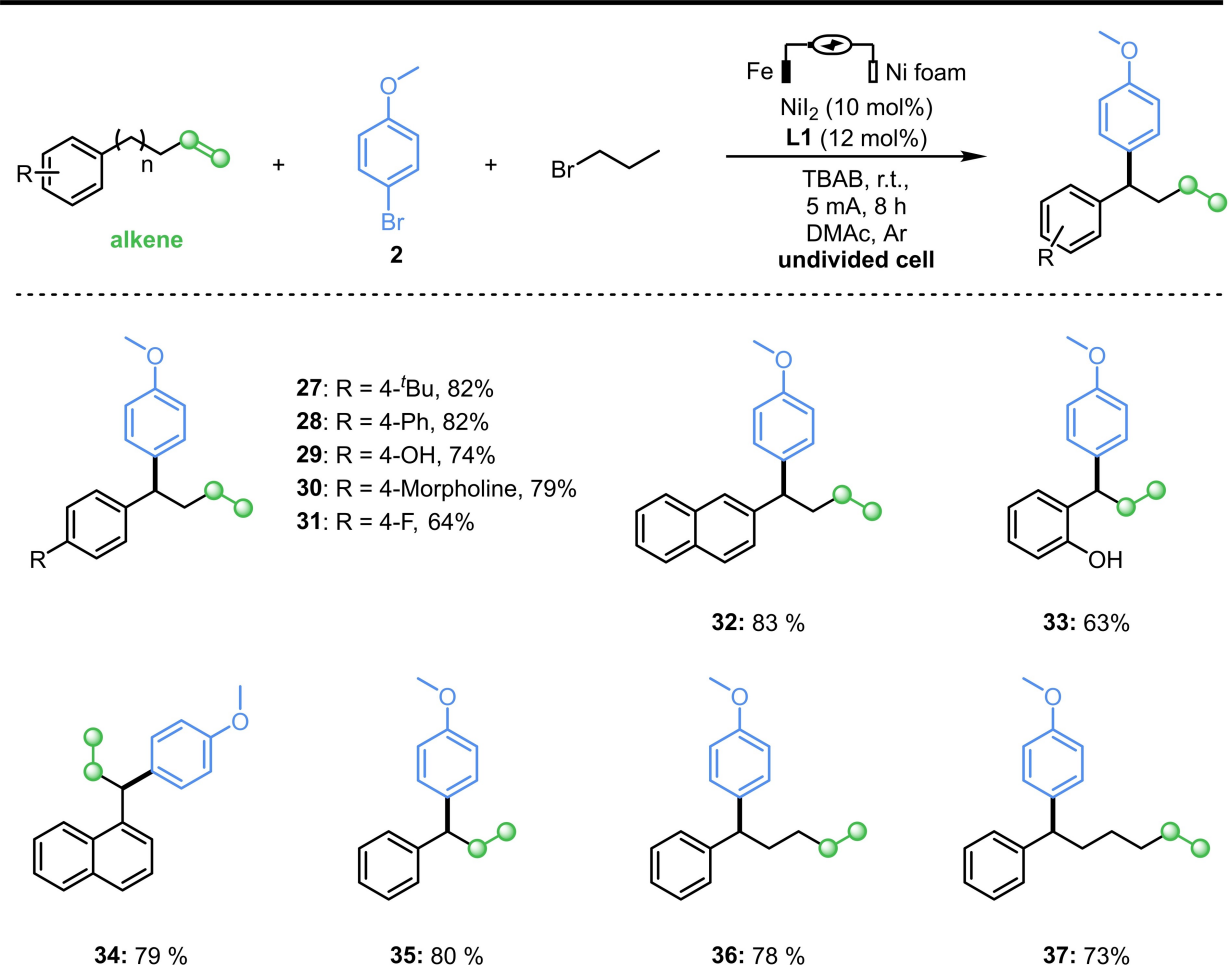
Substrates scope of alkenes.

Next, the influence of alkenes was studied (Scheme 4). The corresponding products from alkene substrates with different substituents (−^*t*^Bu, −Ph, −OH, −Morpholine, −F) could be obtained in good yields (**27**–**31**). Alkenes containing naphthalene ring were also suitable for electrochemical reduction coupling protocol with good yields (**32**, **34**). It was remarkable that *ortho*‐hydroxyl substituted alkene could also obtains target compound in moderate yields (**33**). Moreover, steric hindrance has less impact on alkenes than on aryl bromides. Finally, different alkyl chains of various lengths were examined. The results indicated that the alkenes had an extremely high isomerization efficiency.

**Scheme 5:**
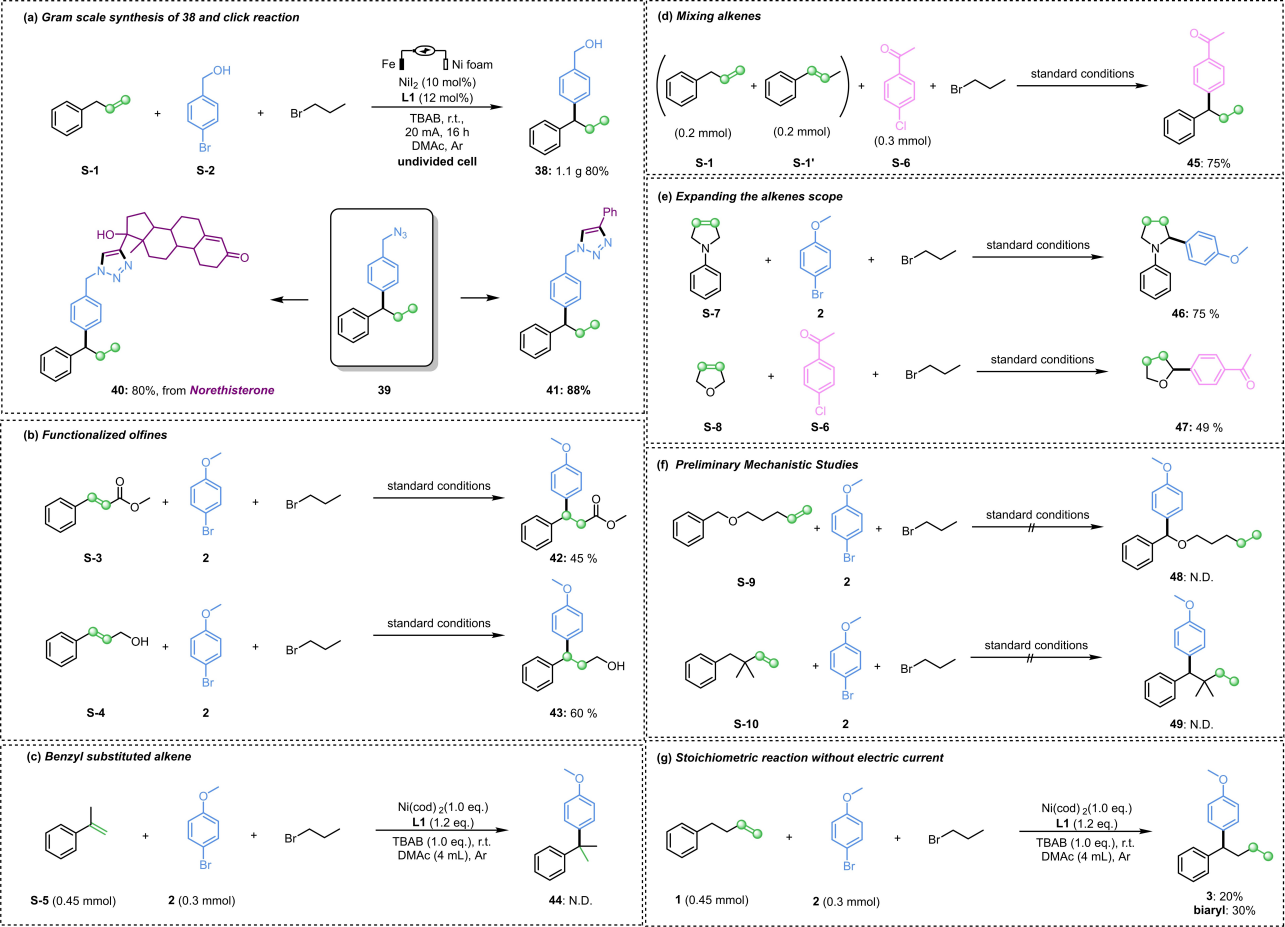
Extension research.

To further demonstrate the synthetic potential of the electro‐reductive protocol, we carried out gram scale experiment and successfully obtained 1.1 g of compound **38** with 80 % yield. Compound **38** was azidated to obtain compound **39**, and the click reaction of compound **39** could be carried out under mild conditions to obtain the cyclization product (Scheme 5a). Subsequently, functionalized alkene substrates were explored, and the target compounds **42** and **43** were obtained. The yields of the ester group and the hydroxyl group on the alkyl chain were 45 % and 60 %, respectively (Scheme 5b). Perhaps the presence of steric hindrance inhibits the formation of metallic species when the substrate **S‐5** with benzyl substituents is used, and thus the target compound **44** cannot be obtained (Scheme 5c). When the alkenes **S‐1** and **S‐1′** were mixed and reacted under standard conditions, a single 1,1‐diaryl product **45** could be obtained in a yield of 75 % with aryl chloride as the substrate (Scheme 5d). In order to expand the range of alkenes, the arylation of *N*‐*α* and *O*‐*α* sites was investigated, and compounds **46** and **47** were obtained with yields of 75 % and 49 %, respectively (Scheme 5e). The alkene substrates **S‐9** and **S‐10** were utilized to verify the mechanism of this reaction. Since sequential β‐H elimination was unable to occur, no corresponding 1,1‐diaryl compounds were detected. Experiments demonstrated that sequential Ni−H addition and β‐H elimination were essential (Scheme 5f). When electricity was substituted by the stoichiometric Ni(0) catalyst, 20 % of **3** was obtained, and the aryl bromide was mainly converted into the homo‐coupling product. The results indicated that the zero‐valence metal catalyst participated in the reaction (Scheme 5g).

In order to gain an understanding of the reaction mechanism, a series of cyclic voltammetry tests were carried out. The reduction potential of **2** is approximately −2.8 V, whereas no reduction potential of the other substrates was observed within the measurement window (Figure S3). A series of nickel catalysts were measured. It was found that the reduction potential from Ni(II) to Ni(I) was approximately −2.2 V, and the reduction potential from Ni(I) to Ni(0) was about −1.0 V. Since Ni(ClO_4_)_2_ ⋅ 6H₂O has a better electrochemical response signal than NiI₂, Ni(ClO_4_)_2_ ⋅ 6H₂O was used in the subsequent tests (Figure S4). In the presence of ligand **L1**, a significant enhancement of the nickel reduction signal was observed (Figure S5). When catalytic amounts of 4‐bromoanisole and 3‐bromopropane were added, a remarkable enhancement of the catalytic current was observed, and the current signal increased as the concentration increased. Since the signal enhancement of 4‐bromoanisole was more pronounced than that of 1‐bromopropane, we hypothesized that the oxidative addition of Ni(0) to aryl halide was more facile than the SET process of Ni(0) with alkyl bromide (Figure S6–8).

**Scheme 6:**
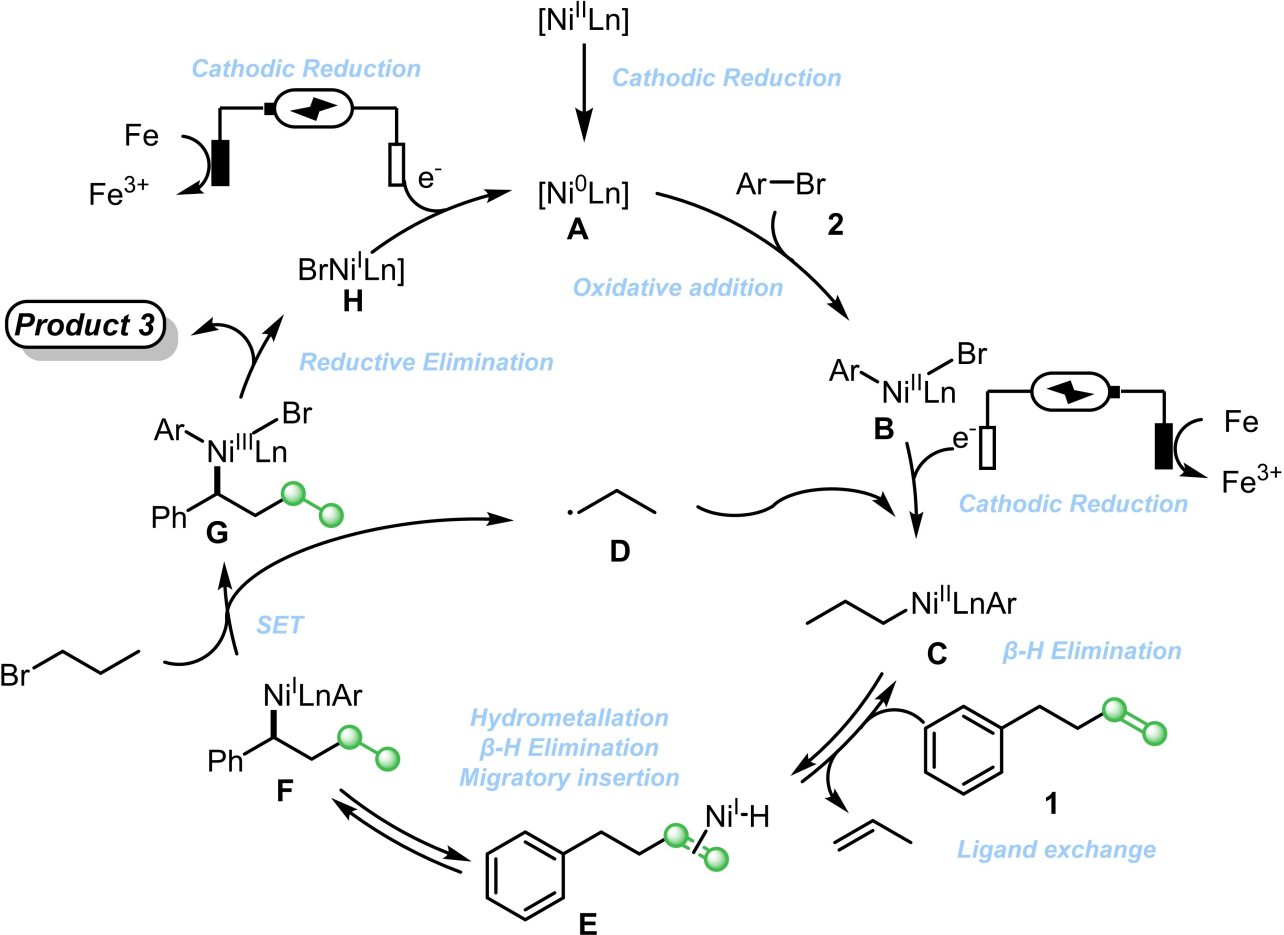
Possible mechanism.

The reaction mechanism was proposed according to previous literatures (Scheme 6).^[32]^ Ni(0) was initially formed with the participation of electric current and ligand. Subsequently, the intermediate **B** was obtained after **A** had undergone the oxidation addition process. Intermediate **B** was reduced by the electrode. Then, it underwent single‐electron oxidation with species **D** to obtain intermediate **C**. Then, the intermediate underwent rapid β‐H elimination and ligand exchange to obtain the intermediate **E**, which underwent a series of hydrogen metallization and β‐H elimination processes to form the thermodynamically dominant intermediate **F**. The intermediate **F** and 1‐bromopropane underwent single‐electron oxidation to obtain the high‐valence ‐state nickel **G**, followed by mild reduction elimination to obtain the target product and Ni(I), and Ni(I) was reduced to Ni(0) under electric current, participating in the next cycle.

## Conclusions

In conclusion, we have developed an electrochemical Ni−H catalyzed arylation coupling method of unactivated alkenes with aryl halides. Electrochemistry and the use of alkyl bromine was a key to the process, avoiding the use of stoichiometric reducing agents and silanes. The mechanism experiments showed that the oxidative addition of Ni(0) to aromatic halide was easier than the single‐electron oxidation of alkyl bromide. The method displays broad functional group tolerance and proceeds under very mild conditions. Furthermore, aryl chlorides were also compatible substrates in this catalytic system. This conversion holds significant implications for the construction of 1,1‐diarylalkanes.

## Supporting Information Summary

The authors have cited additional references within the Supporting Information.[^47^, ^48^, ^49^, ^50^, ^51^, ^52^, ^53^, ^54^, ^55^]

## Conflict of Interests

The authors declare no conflict of interest.

1

## Supporting information

As a service to our authors and readers, this journal provides supporting information supplied by the authors. Such materials are peer reviewed and may be re‐organized for online delivery, but are not copy‐edited or typeset. Technical support issues arising from supporting information (other than missing files) should be addressed to the authors.

Supporting Information

## Data Availability

The data that support the findings of this study are available in the supplementary material of this article.
