# Supplementary material for: Remote Migratory Reductive Arylation of Unactivated Alkenes Enabled by Electrochemical Nickel Catalysis
Source: ChemSusChem. 2024 Nov 19;18(6):e202402196. doi: 10.1002/cssc.202402196 (PMC11911989; doi:10.1002/cssc.202402196)

ChemSusChem

Supporting Information

Remote Migratory Reductive Arylation of Unactivated Alkenes Enabled by Electrochemical Nickel Catalysis

Chao Xu, Ru-Han A, and Xiao-Feng Wu*

1 General remarks....................2
2 Structures of Starting Materials....................3
3 General procedure for the synthesis of alkenes....................4
4. Optimization of reaction conditions....................6
5. General procedure of electroreduction of unactivated alkenes....................11
6. General procedure of gram scale reaction....................12
7. Conversion of **38**....................13
8. Characterization data of products....................14
9. Cyclic voltammetry....................31
10. Reference....................34
11. Spectra of compounds....................35

1 General remarks

Electroreductive reactions were carried out in undivided electrochemical cells (15 mL) using pre-dried glassware, if not noted otherwise. Iron plate electrodes (0.2 mm × 10.0 mm × 20.0 mm, 99.9%; obtained from High purity metal materials Research Institute, Anhui, China), nickel foam electrodes (1.0 mm × 10.0 mm × 20.0 mm, 99.9%; obtained from Keshenghe, Jiangsu, China), were connected using stainless steel adapters. Electroreduction was conducted using an Admiral Squidsta Plus workstation potentiostat in constant current mode. Cyclic Voltammetry studies were performed using an Admiral Squidsta Plus workstation and SUI v2.0 Beat software. Yields refer to isolated compounds, estimated to be >95% purity as determined by ^{1}H-NMR. Flash chromatography was performed using Silica gel (200-300 mesh) purchased from Qingdao Haiyang Chemical Co., China. NMR spectra were recorded on Bruker AVANCE AV 400 in the solvent indicated; chemical shifts (δ) are given in ppm relative to the residual solvent peak. Multiplicities are recorded as: s = singlet, d = doublet, t = triplet, dd = doublet of doublets, m = multiplet.

2 Structures of Starting Materials

Aryl halide

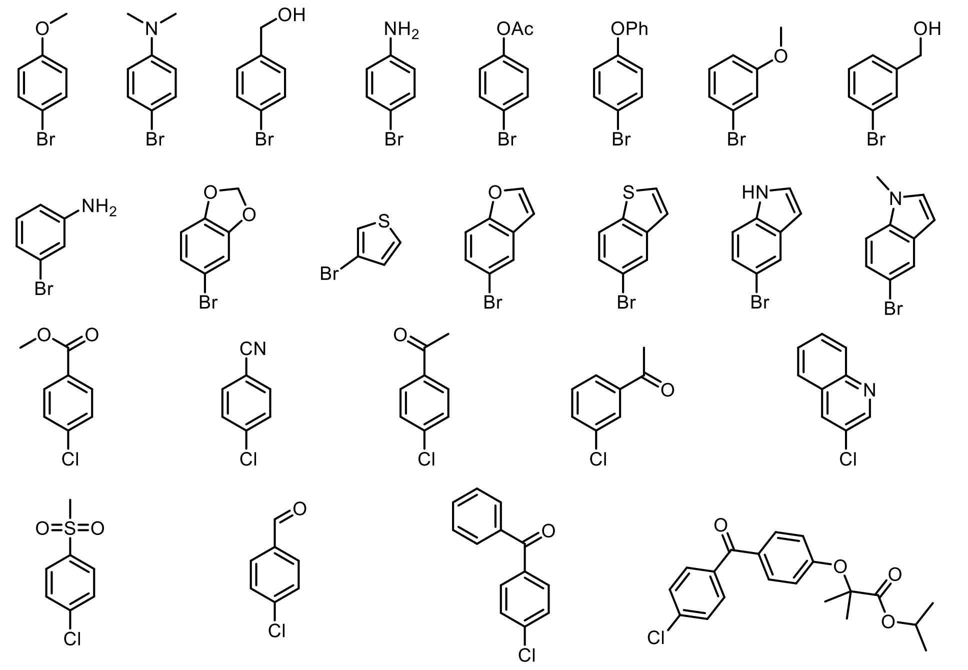

Alkene

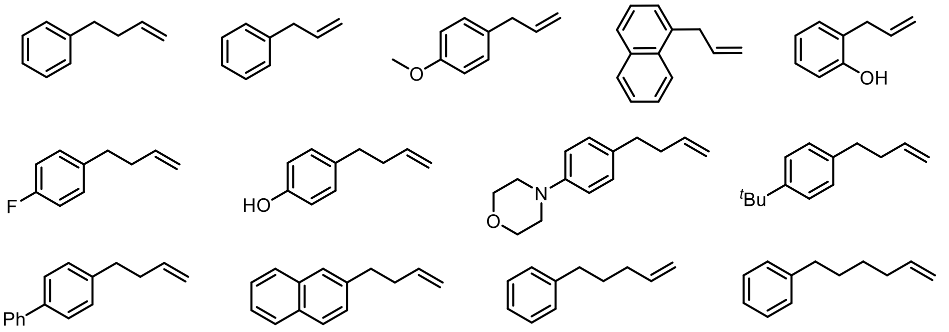

Failed substrates

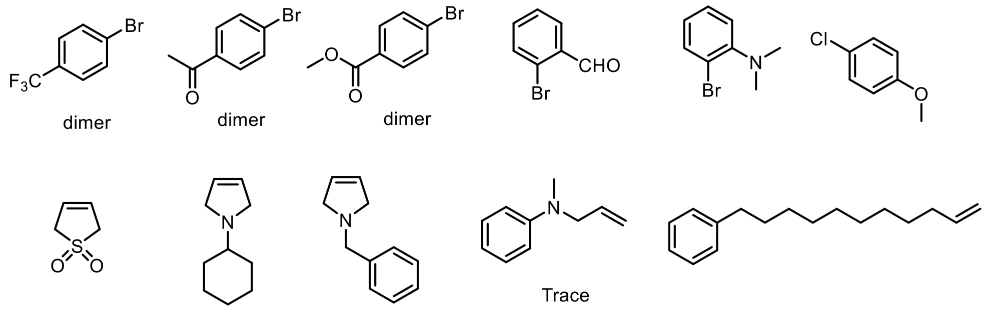

3 General procedure for the synthesis of alkenes

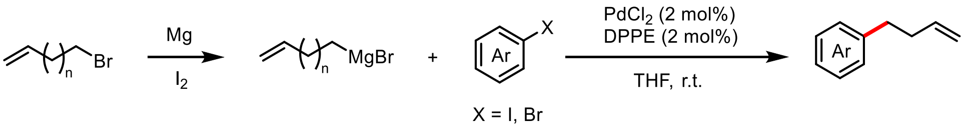

The alkenes were prepared according to our previous work.[1]

Step 1: to an oven dried 100 mL Schlenk tube equipped with a magnetic stir bar, activated magnesium powder (1.3 g, 54 mmol, 1.2 equiv) was added. The equipment was sealed with rubber septum, evacuated, and backfilled with nitrogen and this operation was repeated three times. Then I2 and dry THF (20 mL) were added to the tube. The corresponding 4-bromo-1-butene (4.6 mL, 45 mmol) in dry THF (25 mL) was added dropwise by syringe at room temperature. The reaction mixture was stirred at 45 °C for 2-4 h. Then, the reaction mixture was cooled to room temperature and used directly in the next step.

Step 2: The solvent of but-3-en-1-yl magnesium bromide in THF (6.0 mL, 6.0 mmol, 1M) was added dropwise to a solvent of aryl iodide or bromide (2 mmol), $PdCl_2$ (17.7 mg, 5 mol%) and DPPF (55.4 mg, 5 mol%) in dry THF (10 mL) under nitrogen. Then the reaction mixture was stirred at room temperature for 12 h and quenched by a cold solution of NH_4Cl (sat. aq.). The mixture was extracted with CH_2Cl_2. The combined organic layer was dried over Na_2SO_4, filtered and concentrated under reduced pressure. The crude residue was purified by flash chromatography using pentane to afford the corresponding products.

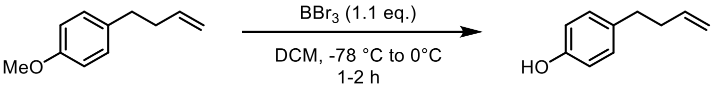

The solvent of 1-(but-3-en-1-yl)-4-methoxybenzene (0.48 g, 3 mmol) in CH_2Cl_2 (6 mL) was cooled to -78 °C, then BBr_3 (3.3 mL, 1M in CH_2Cl_2, 3.3 mmol, 1.1 equiv.) was added dropwise to the solvent. The reaction mixture was stirred at -78 °C for 1 h and then stirred at 0 °C for another 1 h. The reaction was detected by TLC, if it was incomplete, additional BBr_3 (0.9 mL, 1M in CH_2Cl_2, 0.9 mmol, 0.3 equiv.) was added dropwise to the solvent at 0 °C and stirred for 1 h. The mixture was quenched with H_2O and extracted with CH_2Cl_2. The combined organic layer was dried over Na_2SO_4, filtered and concentrated under reduced pressure. The crude product was further purified by flash column chromatograph (pentane/EA = 5:1, R_f = 0.4) to give 4-(but-3-en-1-yl)phenol as a

colorless oil (0.27 g, 62%).

(2,2-Dimethylbut-3-en-1-yl)benzene (S-9)[2]

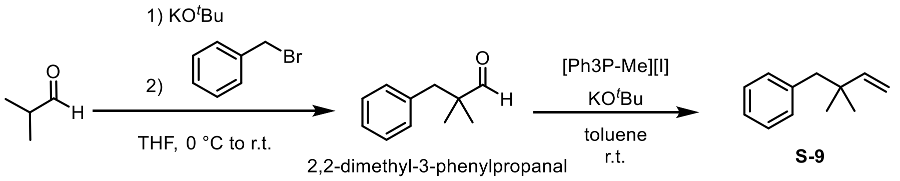

Step 1: Aldehyde S6 was prepared according to a literature procedure. To an oven dried 2-necked 100 mL round bottom flask charged with KO^{t}Bu (2.03 g, 18.1 mmol) and THF (80 mL) was added isobutyraldehyde (1.50 mL, 16.4 mmol) dropwise at 0 °C with strong stirring under N_2. The reaction mixture was allowed to stir for 10 min, at which time benzyl bromide (2.15 mL, 18.1 mmol) was added dropwise. The reaction mixture was allowed to warm to room temperature and stir for 2 h, at which time it was quenched by the addition of water (100 mL). The reaction mixture was extracted with EtOAc (3 x 50 mL). The combined organic extracts were washed with water (2 x 50 mL) and then brine (50 mL). The combined organic extracts were then dried over Na_2SO_4 and concentrated in vacuo to provide 2,2-dimethyl-3-phenylpropanal as a colorless oil and used without further purification.

Step 2: To an oven-dried 3-necked 500 mL round bottom flask charged with methyltriphenylphosphonium iodide (7.70 g, 19.0 mmol) and toluene (150 mL) was added KO^{t}Bu (3.56 g, 31.7 mmol). The reaction mixture was heated at 90 °C for 3 h. The reaction mixture was cooled to room temperature, and 2,2-dimethyl-3-phenylpropanal (2.06 g, 12.7 mmol) was added dropwise. The reaction mixture was allowed to stir for 16 h and was then filtered over a plug of silica, eluting with hexanes to remove phosphine oxide. The filtrate was concentrated in vacuo and purified by column chromatography (hexanes) to provide (2,2-dimethylbut-3-en-1-yl) benzene (22, 0.95 g, 47%) as a clear oil. The ^{1}H and ^{13}C NMR spectral data were identical to those previously reported.

^{1}H NMR (400 MHz, $CDCl_3$) δ 7.28 – 7.24 (m, 2H), 7.21 (tt, J = 7.4, 1.8 Hz, 1H), 7.14 – 7.11 (m, 2H), 5.87 (dd, J = 17.5, 10.7 Hz, 1H), 4.92 (dd, J = 10.7, 1.3 Hz, 1H), 4.86 (dd, J = 17.4, 1.3 Hz, 1H), 2.59 (s, 3H), 1.01 (s, 6H); **^{13}C NMR** (100 MHz, $CDCl_3$) δ 148.1, 138.9, 130.6, 127.6, 125.9, 110.6, 49.1, 37.7, 26.5. **MS (EI)** $[M]^+$ *m/z* 160.

4. Optimization of reaction conditions

Table S1: The effect of equivalent of **1**.

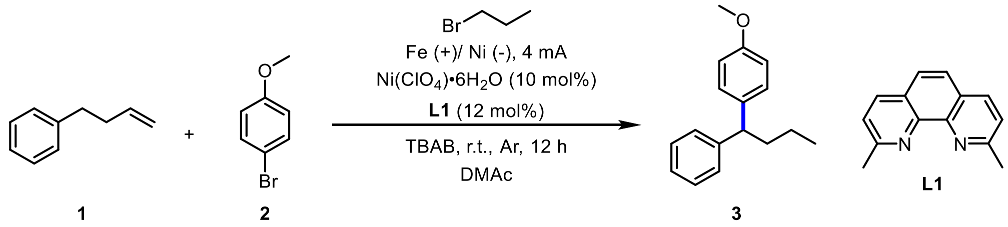

Entry	Equivalent (x eq.)	Yield (%)[a]
1	0.5	61
2	1.0	67
3	1.2	72
4	***1.5***	***78***
5	2.0	76

[a]Reaction conditions: **1** (x eq.), **2** (0.3 mmol), bromopropane (1.5 eq.), $Ni(ClO_4)•6H_2O$ (10 mol%), **L1** (12 mol%), TBAB (0.3 mmol), DMAc (4.0 mL), in an undivided cell, Fe as anode, Ni Foam as cathode, constant current = 4 mA, r.t., 12 h, under Ar atmosphere. Yields were determined by GC with dodecane as an internal standard.

Table S2: The effect of solvents.

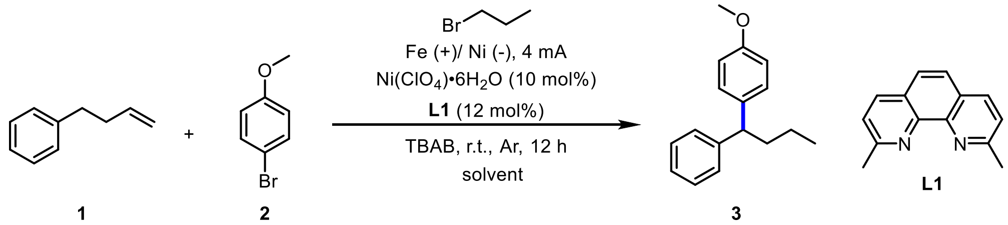

Entry	Solvent	Yield (%)[a]
1	***DMAc***	***78***
2	DMF	22
3	NMP	77
4	MeCN	11
5	Acetone/DMAc (1/1)	35

[a]Reaction conditions: **1** (0.45 mmol), **2** (0.3 mmol), bromopropane (1.5 eq.), $Ni(ClO_4)•6H_2O$ (10 mol%), **L1** (12 mol%), TBAB (0.3 mmol), solvent (4.0 mL), in an undivided cell, Fe as anode, Ni Foam as cathode, constant current = 4 mA, r.t., 12 h, under Ar atmosphere. Yields were determined by GC with dodecane as an internal standard.

Table S3: The effect of [Ni] salts.

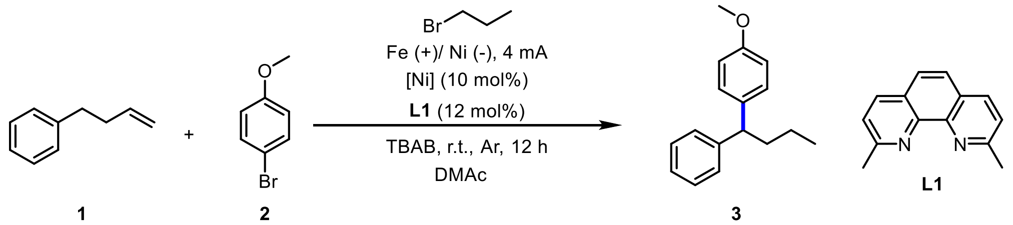

Entry	[Ni] salt	Yield (%)[a]
1	Ni(ClO$_4$)•6H$_2$O	78
2	NiBr$_2$•3H$_2$O	22
3	NiBr$_2$	86
4	NiBr$_2$•DME	85
5	***NiI$_2$***	***90***
6	NiI$_2$•6H$_2$O	76
7	Ni(OTf)$_2$	72
8	NiCl$_2$	72
9	Ni(acac)$_2$	74
10	NiI$_2$ under air	13
11	Without [Ni]	0

[a]Reaction conditions: **1** (0.45 mmol), **2** (0.3 mmol), bromopropane (1.5 eq.), [Ni] (10 mol%), **L1** (12 mol%), TBAB (0.3 mmol), DMAc (4.0 mL), in an undivided cell, Fe as anode, Ni Foam as cathode, constant current = 4 mA, r.t., 12 h, under Ar atmosphere. Yields were determined by GC with dodecane as an internal standard.

Table S4 The effect of ligand. [3]

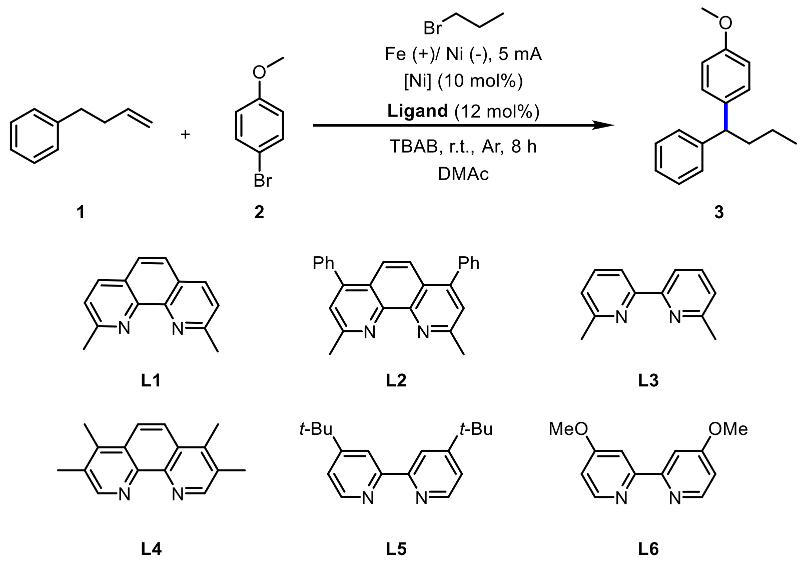

Entry	Anode/Cathode	Yield (%)[a]
1	***L1***	***90***
2	L2	84
3	L3	53
4	L4	Trace
5	L5	Trace
6	L6	Trace
7	Without ligand	0

[a]Reaction conditions: **1** (0.45 mmol), **2** (0.3 mmol), bromopropane (1.5 eq.), [Ni] (10 mol%), **L1** (12 mol%), TBAB (0.3 mmol), DMAc (4.0 mL), in an undivided cell, Fe as anode, Ni Foam as cathode, constant current = 5 mA, r.t., 8 h, under Ar atmosphere. Yields were determined by GC with dodecane as an internal standard.

Table S5: The effect of electrodes.

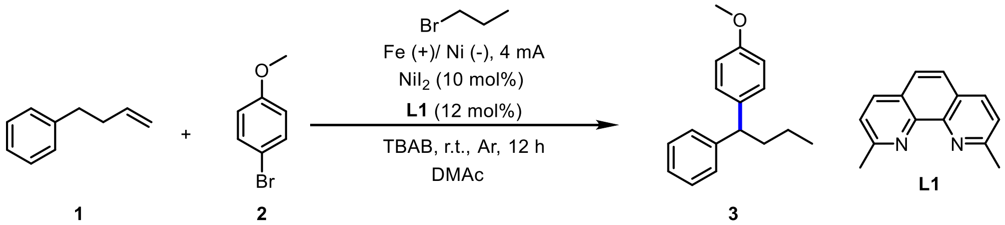

Entry	Anode/Cathode	Yield (%)[a]
1	***Fe(+)/Ni Foam(-)***	***90***
2	Fe(+)/RVC(-)	74
3	Fe(+)/GF(-)	38
4	Fe(+)/Pt(-)	24
5	Zn(+)/Ni foam(-)	6

[a]Reaction conditions: **1** (0.45 mmol), **2** (0.3 mmol), bromopropane (1.5 eq.), NiI_2 (10 mol%), **L1** (12 mol%), TBAB (0.3 mmol), DMAc (4.0 mL), in an undivided cell, constant current = 4 mA, r.t., 12 h, under Ar atmosphere. Yields were determined by GC with dodecane as an internal standard.

Table S6 The effect of constant current.

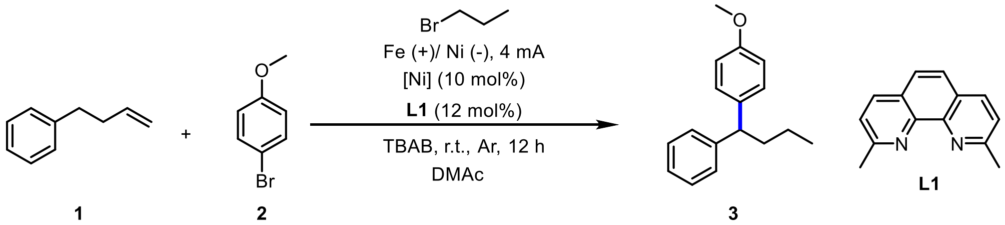

Entry	Current (X mA)	F/ mol	Yield (%)[a]
1	4 (12 h)	6.0	90
2	4 (10 h)	5.0	92.2
3	4 (8 h)	4.0	83
3	***5 (8 h)***	***5.0***	***92.4(84[b])***
4	5 (6 h)	3.8	86
5	5 (5 h)	3.1	82
6	6 (8 h)	6.0	87
7	6 (6 h)	4.5	82
8	6 (5 h)	3.7	80
9	Without electric	0	0

[a]Reaction conditions: **1** (0.45 mmol), **2** (0.3 mmol), bromopropane (1.5 eq.), [Ni] (10 mol%),

L1 (12 mol%), TBAB (0.3 mmol), DMAc (4.0 mL), in an undivided cell, Fe as anode, Ni Foam as cathode, constant current = X mA, r.t., 12 h, under Ar atmosphere. Yields were determined by GC with dodecane as an internal standard. [b] Isolated yield.

Table S7 The effect of alkyl bromines

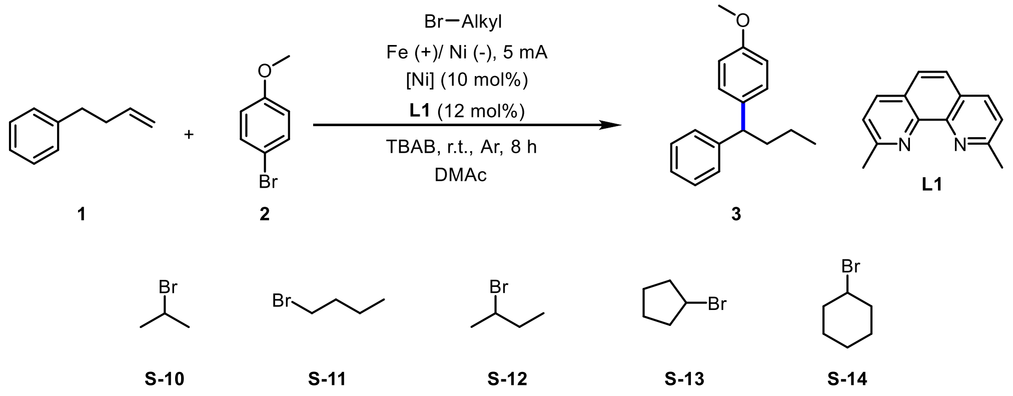

Entry	Alkyl bromide	Yield (%)[a]
1	**S-10**	64
2	**S-11**	78
3	**S-12**	65
4	**S-13**	34
5	**S-14**	79

[a]Reaction conditions: **1** (0.45 mmol), **2** (0.3 mmol), Alkyl bromine (1.5 eq.), [Ni] (10 mol%), **L1** (12 mol%), TBAB (0.3 mmol), DMAc (4.0 mL), in an undivided cell, Fe as anode, Ni Foam as cathode, constant current = 5 mA, r.t., 8 h, under Ar atmosphere. Yields were determined by GC with dodecane as an internal standard.

5. General procedure of electroreduction of unactivated alkenes

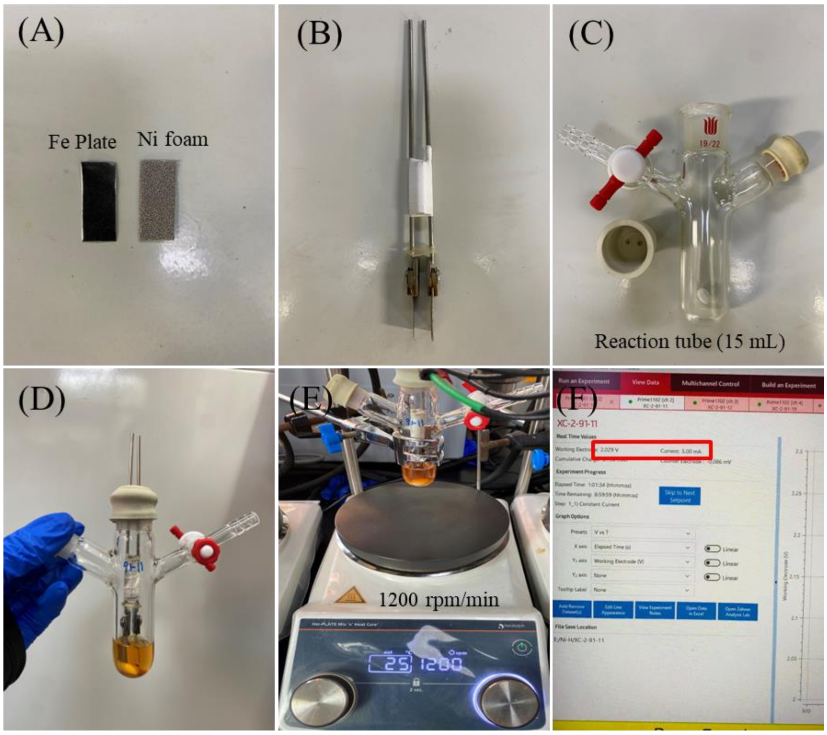

Figure S1. Pictures of the reaction setups for electrochemical reduction of unactivated alkenes

The electrolysis process of 0.3 mmol scale was carried out in an undivided cell with a Fe plate anode (10 mm × 20 mm × 0.20 mm) and a Ni Foam cathode (10 mm × 20 mm × 0.30 mm). To a 15 mL oven-dried undivided electrochemical cell equipped with a magnetic bar was added NiI_2 (9.4 mg, 10 mol%), **L1** (7.5 mg, 12 mol%) and $^{n}Bu_4NBr$ (96.7 mg, 0.30 mmol). The reaction tube was transferred to the glove box filled with argon gas. Next, the alkene (0.45 mmol, 1.5 eq.), aryl halogen (0.3 mmol, 1.0 eq.) and 1-bromopropane (33 μL, 1.5 eq.) dissolved in anhydrous DMAc (4.0 mL) was injected into the tube. After that, the reaction mixture was electrolyzed under a constant current of 5 mA for 8 h. After the reaction was completed, the mixture was diluted with sat. NH_4Cl (about 40 mL) and washed with EtOAc (3 x 40 mL), dried over Na_2SO_4, filtered, and concentrated in vacuo. Remove residual DMA with oil pump. The crude product was purified by column chromatography to furnish the desired product.

6. General procedure of gram scale reaction

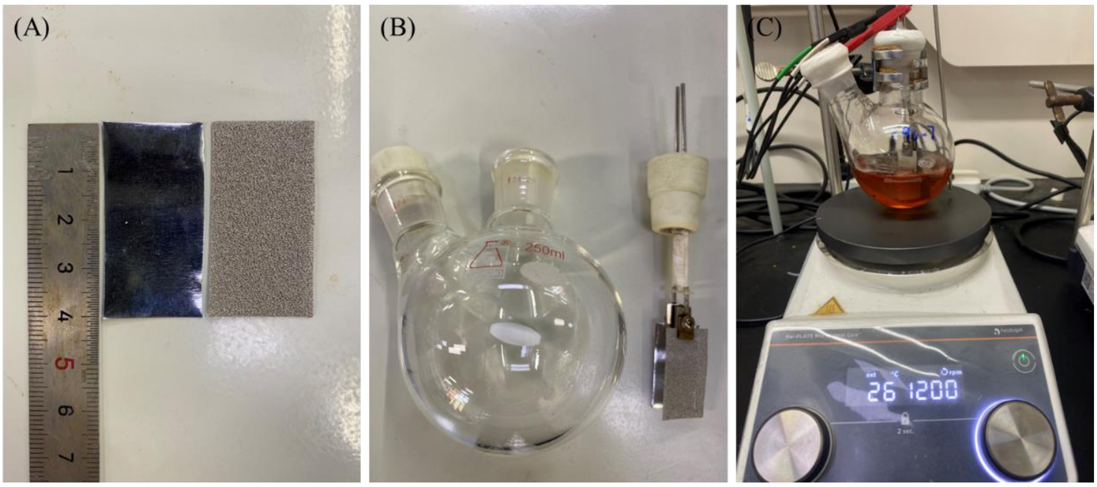

Figure S2. Pictures of the reaction setups for the gram scale reaction

The electrolysis process of 6 mmol scale was carried out in a 250ml double-neck round bottom flask with a Fe plate anode (20 mm × 40 mm × 0.20 mm) and a Ni Foam cathode (20 mm × 40 mm × 0.30 mm). The flask equipped with a magnetic bar was added NiI_2 (188 mg, 10 mol%), **L1** (150 mg, 12 mol%) and $^{n}Bu_4NBr$ (1.9 g, 6 mmol). The reaction tube was transferred to the glove box filled with argon gas. Next, the alkene (0.45 mmol, 1.5 eq.), aryl halogen (0.3 mmol, 1.0 eq.) and 1-bromopropane (660 µL, 1.5 eq.) dissolved in anhydrous DMAc (40 mL) was injected into the flask. After that, the reaction mixture was electrolyzed under a constant current of 20 mA for 16 h. The reaction was monitored by GC, after the reaction was completed, the mixture was diluted with sat. NH_4Cl and washed with EtOAc, dried over Na_2SO_4, filtered, and concentrated in vacuo. Remove residual DMA with oil pump. The crude product was purified by column chromatography to furnish the desired product (1.1 g, 80%).

7. Conversion of 38

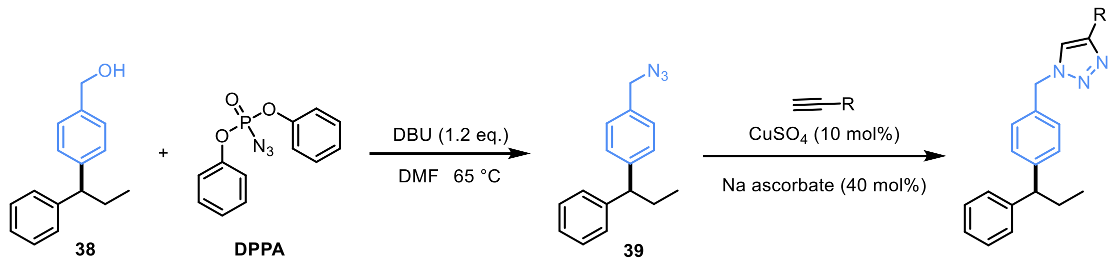

Step 1: A mixture of **38** (4 mmol) and diphenylphosphoryl azide (1.2 eq.) were dissolved in dry solvent (7 mL). To the mixture at 0 °C, under N_2 atmosphere was added DBU (1.2 eq.). The reaction was warmed to room temperature and stirred until complete. The resulting mixture was washed with H_2O and 5% HCl. The organic phase was concentrated in vacuum and purified by silica gel chromatography using petroleum ether.

Step 2: To a 10 mL Schlenk tube azide product (0.1 mmol), aryl alkynes (0.3 mmol), $CuSO_4$ (10 mol%) and sodium *L*-ascorbate (40 mol%) and DMF (1 mL) were added. The mixture was stirred at room temperature overnight. The mixture was washed with H_2O and EtOAc, dried over Na_2SO_4, filtered, and concentrated in vacuo. The crude product was purified by column chromatography to furnish the desired product.[4]

8. Characterization data of products

1-Methoxy-4-(1-phenylbutyl) benzene (3)

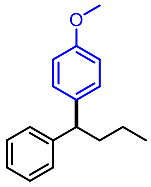

The title compound was prepared following the general procedure, purification by column chromatography on silica gel (petroleum ether/EtOAc = 100:1) yielded (60 mg, 84%) as a colorless oil. **^{1}H NMR** (400 MHz, $CDCl_3$) δ 7.30 – 7.19 (m, 4H), 7.18 – 7.09 (m, 3H), 6.88 – 6.76 (m, 2H), 3.85 (d, *J* = 8.0 Hz, 1H), 3.74 (s, 3H), 2.19 – 1.83 (m, 2H), 1.42 – 1.15 (m, 2H), 0.91 (t, *J* = 7.6 Hz, 3H). **^{13}C NMR** (100 MHz, $CDCl_3$) δ 157.8, 145.8, 137.5, 128.8, 128.4, 127.8, 125.9, 113.8, 55.2, 50.2, 38.1, 21.2, 14.1. **MS (EI)** $[M]^+$ *m*/*z* 240.

***N, N*-dimethyl-4-(1-phenylbutyl) aniline (4)**

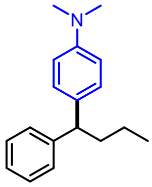

The title compound was prepared following the general procedure, purification by column chromatography on silica gel (petroleum ether/EtOAc = 20:1) yielded (53 mg, 70%) as a colorless oil. **^{1}H NMR** (400 MHz, $CDCl_3$) δ 7.30 – 7.19 (m, 4H), 7.16 – 7.05 (m, 3H), 6.67 (d, *J* = 8.7 Hz, 1H), 3.81 (d, *J* = 8.0 Hz, 1H), 2.88 (s, 6H), 2.09 – 1.91 (m, 2H), 1.54 – 1.15 (m, 2H), 0.91 (t, *J* = 7.6 Hz, 3H). ^{13}C NMR (100 MHz, $CDCl_3$) δ 149.0, 146.3, 133.5, 128.4, 128.3, 127.8, 125.7, 112.8, 50.1, 40.8, 38.2, 21.3, 14.1. **MS (EI)** $[M]^+$ *m*/*z* 253.

(4-(1-Phenylbutyl) phenyl) methanol (5)

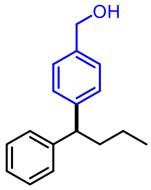

The title compound was prepared following the general procedure, purification by column chromatography on silica gel (petroleum ether/EtOAc = 10:1) yielded (51 mg, 72%) as a colorless oil. **^{1}H NMR** (400 MHz, $CDCl_3$) δ 7.36 – 7.18 (m, 9H), 7.19 – 7.10 (m, 1H), 4.59 (s, 2H), 3.90 (d,

J = 8.0 Hz, 1H), 2.46 – 1.96 (m, 2H), 1.48 – 1.14 (m, 2H), 0.91 (t, J = 7.6 Hz, 3H). **^{13}C NMR** (100 MHz, $CDCl_3$) δ 145.3, 144.9, 138.6, 128.4, 128.1, 127.9, 127.3, 126.1, 65.2, 50.8, 37.9, 21.2, 14.1. HRMS (EI) calculated for $C_{17}H_{20}O$ $[M]^+$ 240.3460, found 240.3462.

4-(1-Phenylbutyl) aniline (6)

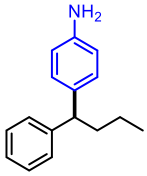

The title compound was prepared following the general procedure, purification by column chromatography on silica gel (petroleum ether/EtOAc = 5:1) yielded (30 mg, 45%) as a colorless oil. **^{1}H NMR** (400 MHz, $CDCl_3$) δ 7.34 – 7.19 (m, 5H), 7.17 – 7.10 (m, 1H), 7.05 – 7.00 (m, 2H), 6.78 – 6.57 (m, 2H), 3.80 (t, J = 7.6 Hz, 1H), 2.13 – 1.85 (m, 2H), 1.33 – 1.21 (m, 2H), 0.91 (t, J = 7.2 Hz, 3H). **^{13}C NMR** (100 MHz, $CDCl_3$) δ 146.1, 144.0, 135.8, 128.7, 128.3, 127.8, 125.8, 115.4, 50.2, 38.1, 21.2, 14.1. **MS (EI)** $[M]^+$ m/z 225.

4-(1-Phenylbutyl) phenyl acetate (7)

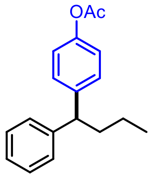

The title compound was prepared following the general procedure, purification by column chromatography on silica gel (petroleum ether/EtOAc = 30:1) yielded (51 mg, 64%) as a colorless oil. **^{1}H NMR** (400 MHz, $CDCl_3$) δ 7.30 – 7.20 (m, 6H), 7.18 – 7.13 (m, 1H), 7.03 – 6.93 (m, 2H), 3.90 (d, J = 8.0 Hz, 1H), 2.25 (s, 3H), 2.08 – 1.95 (m, 2H), 1.40 – 1.17 (m, 2H), 0.91 (t, J = 7.6 Hz, 3H). **^{13}C NMR** (100 MHz, $CDCl_3$) δ 169.6, 148.8, 145.0, 142.9, 128.8, 128.4, 127.9, 126.1, 121.3, 50.5, 38.0, 21.2, 21.1, 14.1. **MS (EI)** $[M]^+$ m/z 268.

1-Phenoxy-4-(1-phenylbutyl) benzene (8)

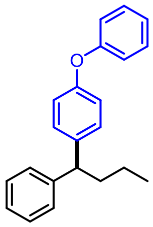

The title compound was prepared following the general procedure, purification by column chromatography on silica gel (petroleum ether/EtOAc = 50:1) yielded (69 mg, 77%) as a colorless oil. **^{1}H NMR** (400 MHz, $CDCl_3$) δ 7.34 – 7.22 (m, 6H), 7.21 – 7.14 (m, 3H), 7.10 – 7.03 (m, 1H), 7.01 – 6.95 (m, 2H), 6.95 – 6.88 (m, 2H), 3.89 (d, J = 8.0 Hz, 1H), 2.11 – 1.92 (m, 2H), 1.39 – 1.20 (m, 2H), 0.92 (t, J = 7.6 Hz, 3H). **^{13}C NMR** (100 MHz, $CDCl_3$) δ 157.5, 155.3, 145.4, 140.3, 129.7, 129.0, 128.4, 127.9, 126.1, 123.0, 118.9, 118.7, 50.4, 38.1, 21.2, 14.1. **MS (EI)** $[M]^+$ m/z 302.

1-Methoxy-3-(1-phenylbutyl) benzene (9)

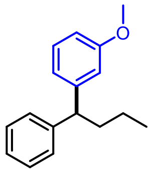

The title compound was prepared following the general procedure, purification by column chromatography on silica gel (petroleum ether/EtOAc = 100:1) yielded (59 mg, 82%) as a colorless oil. **^{1}H NMR** (400 MHz, $CDCl_3$) δ 7.32 – 7.21 (m, 4H), 7.21 – 7.12 (m, 2H), 6.84 (dd, J = 8.0, 1.2 Hz, 1H), 6.79 (t, J = 2.1 Hz, 1H), 6.74 – 6.65 (m, 1H), 3.87 (t, J = 8.0 Hz, 1H), 3.76 (s, 3H), 2.07 – 1.95 (m, 2H), 1.27 (tt, J = 8.4, 6.4 Hz, 2H), 0.91 (t, J = 7.6 Hz, 3H). **^{13}C NMR** (100 MHz, $CDCl_3$) δ 159.6, 147.0, 145.2, 129.3, 128.4, 128.3, 127.9, 126.1, 120.4, 114.1, 110.8, 55.1, 51.1, 37.9, 21.2, 14.1. **MS (EI)** $[M]^+$ m/z 240.

(3-(1-Phenylbutyl) phenyl) methanol (10)

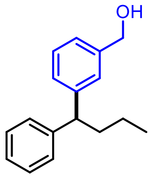

The title compound was prepared following the general procedure, purification by column chromatography on silica gel (petroleum ether/EtOAc = 10:1) yielded (46 mg, 65%) as a colorless oil. **^{1}H NMR** (400 MHz, $CDCl_3$) δ 7.25 (qd, J = 6.3, 2.9 Hz, 6H), 7.19 – 7.11 (m, 3H), 4.61 (s, 2H),

3.91 (t, *J* = 8.0 Hz, 1H), 2.09 – 1.94 (m, 2H), 1.36 – 1.18 (m, 2H), 0.91 (t, *J* = 7.6 Hz, 3H). **^{13}C NMR** (101 MHz, $CDCl_3$) δ 145.8, 145.2, 140.9, 128.6, 128.4, 127.9, 127.2, 126.6, 126.1, 124.7, 65.5, 51.1, 37.9, 21.2, 14.1. HRMS (EI) calculated for $C_{17}H_{20}O$ $[M]^+$ 240.3460, found 240.3459.

3-(1-phenylbutyl) aniline (11)

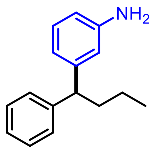

The title compound was prepared following the general procedure, purification by column chromatography on silica gel (petroleum ether/EtOAc = 5:1) yielded (45 mg, 67%) as a colorless oil. **^{1}H NMR** (400 MHz, $CDCl_3$) δ 7.31 – 7.21 (m, 4H), 7.19 – 7.12 (m, 1H), 7.05 (t, *J* = 8.0 Hz, 1H), 6.66 (d, *J* = 7.6 Hz, 1H), 6.53 (t, *J* = 2.0 Hz, 1H), 6.48 (dd, *J* = 8.0, 2.4 Hz, 1H), 3.80 (t, *J* = 8.0 Hz, 1H), 3.34 (s, 3H), 2.19 – 1.92 (m, 2H), 1.40 – 1.18 (m, 2H), 0.91 (t, *J* = 7.4 Hz, 3H). **^{13}C NMR** (100 MHz, $CDCl_3$) δ 146.6, 146.3, 145.4, 129.2, 128.3, 127.9, 125.9, 118.3, 114.8, 113.0, 51.0, 37.8, 21.2, 14.1. **MS (EI)** $[M]^+$ *m*/*z* 225.

5-(1-Phenylbutyl) benzo[d] [1,3] dioxole (12)

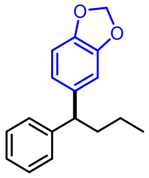

The title compound was prepared following the general procedure, purification by column chromatography on silica gel (petroleum ether/EtOAc = 100:1) yielded (60 mg, 79%) as a colorless oil. **^{1}H NMR** (400 MHz, $CDCl_3$) δ 7.33 – 7.19 (m, 4H), 7.18 – 7.13 (m, 1H), 6.71 (s, 3H), 5.88 (d, *J* = 1.2 Hz, 1H), 3.82 (d, *J* = 8.0 Hz, 1H), 2.05 – 1.88 (m, 2H), 1.40 – 1.17 (m, 2H), 0.91 (t, *J* = 7.6 Hz, 3H). **^{13}C NMR** (100 MHz, $CDCl_3$) δ 147.7, 145.7, 145.5, 139.4, 128.4, 127.7, 126.1, 120.8, 108.3, 108.1, 100.8, 50.7, 38.0, 21.2, 14.1. **MS (EI)** $[M]^+$ *m*/*z* 254.

3-(1-Phenylbutyl) thiophene (13)

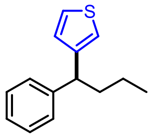

The title compound was prepared following the general procedure, purification by column chromatography on silica gel (petroleum ether/EtOAc = 100:1) yielded (47 mg, 73%) as a colorless

oil. **^{1}H NMR** (400 MHz, $CDCl_3$) δ 7.37 – 7.14 (m, 6H), 6.98 (d, *J* = 2.8 Hz, 1H), 6.90 (dd, *J* = 4.8, 1.2 Hz, 1H), 3.96 (d, *J* = 8.0 Hz, 1H), 2.15 – 1.83 (m, 2H), 1.38 – 1.12 (m, 2H), 0.91 (t, *J* = 7.6 Hz, 3H). **^{13}C NMR** (100 MHz, $CDCl_3$) δ 146.4, 145.0, 128.4, 127.9, 127.8, 126.2, 125.3, 119.9, 46.7, 38.4, 21.1, 14.1. **MS (EI)** $[M]^+$ *m*/*z* 216.

5-(1-Phenylbutyl) benzofuran (14)

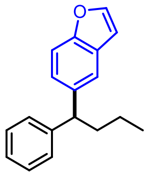

The title compound was prepared following the general procedure, purification by column chromatography on silica gel (petroleum ether/EtOAc = 100:1) yielded (62 mg, 83%) as a colorless oil. **^{1}H NMR** (400 MHz, $CDCl_3$) δ 7.55 (d, *J* = 2.4 Hz, 1H), 7.46 (d, *J* = 1.6 Hz, 1H), 7.38 (dt, *J* = 8.8, 0.8 Hz, 1H), 7.29 – 7.24 (m, 5H), 7.19 – 7.11 (m, 2H), 6.68 (dd, *J* = 2.0, 0.8 Hz, 1H), 4.00 (d, *J* = 8.0 Hz, 1H), 2.51 – 1.86 (m, 2H), 1.67 – 1.18 (m, 2H), 0.92 (t, *J* = 7.6 Hz, 3H). **^{13}C NMR** (100 MHz, $CDCl_3$) δ 153.6, 145.8, 145.1, 134.0, 128.4, 127.9, 127.5, 126.0, 124.6, 119.9, 111.1, 106.6, 50.9, 38.3, 21.3, 14.1. **MS (EI)** $[M]^+$ *m*/*z* 250.

5-(1-Phenylbutyl) benzo[b]thiophene (15)

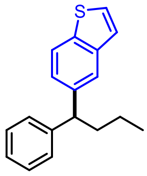

The title compound was prepared following the general procedure, purification by column chromatography on silica gel (petroleum ether/EtOAc = 100:1) yielded (65 mg, 82%) as a colorless oil. **^{1}H NMR** (400 MHz, $CDCl_3$) δ 7.75 (d, *J* = 8.4 Hz, 1H), 7.69 (d, *J* = 2.0 Hz, 1H), 7.37 (d, *J* = 5.2 Hz, 1H), 7.30 – 7.25 (m, 5H), 7.24 – 7.19 (m, 1H), 7.15 (h, *J* = 4.4 Hz, 1H), 4.03 (d, *J* = 8.0 Hz, 1H), 2.21 – 1.90 (m, 2H), 1.47 – 1.20 (m, 2H), 0.93 (t, *J* = 7.6 Hz, 3H). **^{13}C NMR** (100 MHz, $CDCl_3$) δ 145.5, 141.6, 139.9, 137.5, 128.4, 127.9, 126.5, 126.1, 125.0, 123.9, 122.5, 122.4, 50.9, 38.1, 21.2, 14.1. **MS (EI)** $[M]^+$ *m*/*z* 266.

5-(1-Phenylbutyl)-1*H*-indole (16)

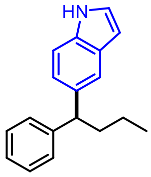

The title compound was prepared following the general procedure, purification by column chromatography on silica gel (petroleum ether/acetone = 24:1) yielded (48 mg, 64%) as a colorless oil. **^{1}H NMR** (400 MHz, $CDCl_3$) δ 7.96 (s, 1H), 7.52 (d, *J* = 2.0 Hz, 1H), 7.36 – 7.20 (m, 5H), 7.17 – 7.08 (m, 2H), 7.05 (dd, *J* = 8.4, 1.6 Hz, 1H), 6.56 – 6.30 (m, 1H), 4.00 (d, *J* = 8.0 Hz, 1H), 2.07 (qd, *J* = 7.2, 2.4 Hz, 2H), 1.43 – 1.20 (m, 2H), 0.92 (t, *J* = 7.6 Hz, 3H). **^{13}C NMR** (100 MHz, $CDCl_3$) δ 146.4, 136.89, 134.4, 128.3, 127.9, 125.7, 124.3, 122.7, 119.3, 110.9, 102.5, 51.1, 38.4, 21.3, 14.2. **MS (EI)** $[M]^+$ *m*/*z* 249.

1-Methyl-5-(1-phenylbutyl)-1*H*-indole (17)

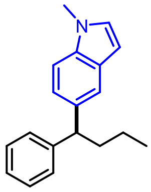

The title compound was prepared following the general procedure, purification by column chromatography on silica gel (petroleum ether/EtOAc = 30:1) yielded (53 mg, 67%) as a colorless oil. **^{1}H NMR** (400 MHz, $CDCl_3$) δ 7.51 (s, 1H), 7.35 – 7.20 (m, 6H), 7.16 – 7.04 (m, 2H), 7.00 (d, *J* = 3.2 Hz, 1H), 6.41 (d, *J* = 3.2 Hz, 1H), 4.01 (d, *J* = 8.0 Hz, 1H), 3.73 (s, 3H), 2.41 – 1.85 (m, 2H), 1.47 – 1.09 (m, 3H), 0.92 (t, *J* = 7.6 Hz, 3H). ^{13}C NMR (101 MHz, $CDCl_3$) δ 146.5, 136.3, 135.4, 128.9, 128.5, 128.3, 127.9, 125.7, 122.2, 119.5, 109.1, 100.7, 51.0, 38.4, 32.9, 21.3, 14.2. **MS (EI)** $[M]^+$ *m*/*z* 263.

1-(4-(1-Phenylbutyl) phenyl) ethan-1-one (18)

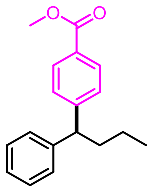

The title compound was prepared following the general procedure, purification by column chromatography on silica gel (petroleum ether/EtOAc = 50:1) yielded (64 mg, 80%) as a colorless oil. **^{1}H NMR** (400 MHz, $CDCl_3$) δ 7.98 – 7.90 (m, 2H), 7.33 – 7.25 (m, 4H), 7.24 – 7.17 (m, 3H),

3.96 (t, J = 8.0 Hz, 1H), 3.88 (s, 3H), 2.21 – 1.89 (m, 2H), 1.40 – 1.15 (m, 2H), 0.92 (t, J = 7.2 Hz, 3H). ^{13}**C NMR** (100 MHz, CDCl3) δ 167.1, 150.8, 144.4, 129.8, 128.5, 128.0, 127.9, 127.9, 126.3, 52.0, 51.1, 37.6, 21.1, 14.0. **MS (EI)** $[M]^+$ m/z 268.

4-(1-Phenylbutyl) benzonitrile (19)

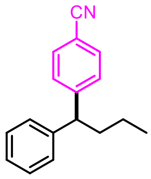

The title compound was prepared following the general procedure, purification by column chromatography on silica gel (petroleum ether/EtOAc = 30:1) yielded (51 mg, 72%) as a colorless oil. ^{1}**H NMR** (400 MHz, $CDCl_3$) δ 7.55 (d, J = 8.4 Hz, 2H), 7.38 – 7.26 (m, 4H), 7.23 – 7.16 (m, 3H), 3.95 (t, J = 8.0 Hz, 1H), 2.23 – 1.76 (m, 2H), 1.48 – 1.14 (m, 2H), 0.92 (t, J = 7.6 Hz, 3H). ^{13}**C NMR** (100 MHz, $CDCl_3$) δ 151.0, 143.6, 132.3, 128.7, 127.8, 126.6, 119.0, 109.9, 51.1, 37.4, 21.0, 14.0. **MS (EI)** $[M]^+$ m/z 235.

1-(4-(1-Phenylbutyl) phenyl) ethan-1-one (20)

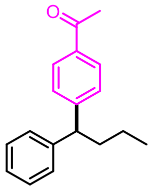

The title compound was prepared following the general procedure, purification by column chromatography on silica gel (petroleum ether/EtOAc = 100:1) yielded (62 mg, 82%) as a colorless oil. ^{1}**H NMR** (400 MHz, $CDCl_3$) δ 7.93 – 7.82 (m, 2H), 7.33 (d, J = 8.4 Hz, 2H), 7.30 – 7.24 (m, 2H), 7.23 – 7.14 (m, 3H), 3.97 (t, J = 8.0 Hz, 1H), 2.55 (s, 3H), 2.19 – 1.92 (m, 2H), 1.41 – 1.20 (m, 2H), 0.92 (t, J = 7.6 Hz, 3H). ^{13}**C NMR** (100 MHz, $CDCl_3$) δ 197.8, 151.0, 144.3, 135.2, 128.6, 128.55, 128.1, 127.8, 126.4, 51.1, 37.6, 26.6, 21.1, 14.0. **MS (EI)** $[M]^+$ m/z 252.

1-(Methylsulfonyl)-4-(1-phenylbutyl) benzene (21)

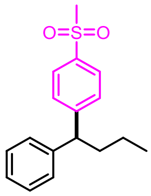

The title compound was prepared following the general procedure, purification by column chromatography on silica gel (petroleum ether/acetone = 20:1) yielded (71 mg, 83%) as a colorless

oil. **¹H NMR** (400 MHz, $CDCl_3$) δ 7.87 – 7.80 (m, 2H), 7.50 – 7.40 (m, 2H), 7.36 – 7.27 (m, 2H), 7.24 – 7.17 (m, 3H), 4.00 (d, *J* = 8.0 Hz, 1H), 3.02 (s, 3H), 2.15 – 1.93 (m, 2H), 1.39 – 1.18 (m, 2H), 0.93 (t, *J* = 7.6 Hz, 3H). **¹³C NMR** (100 MHz, $CDCl_3$) δ 151.9, 143.7, 138.2, 128.8, 128.7, 127.8, 127.6, 126.6, 51.0, 44.6, 37.5, 21.0, 14.0. **MS (EI)** $[M]^+$ *m*/*z* 288.

Isopropyl-2-(4-(4-(1-(4-methoxyphenyl) propyl) benzoyl) phenoxy)-2-methylpropanoate (22)

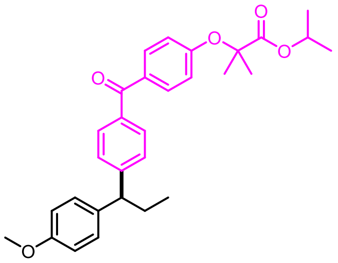

The title compound was prepared following the general procedure, purification by column chromatography on silica gel (petroleum ether/EtOAc = 5:1) yielded (109.5 mg, 77%) as a colorless oil. **¹H NMR** (400 MHz, $CDCl_3$) δ 7.74 (d, *J* = 8.8 Hz, 2H), 7.68 (d, *J* = 8.0 Hz, 2H), 7.31 (d, *J* = 8.4 Hz, 2H), 7.16 (d, *J* = 8.8 Hz, 2H), 6.84 (dd, *J* = 8.8, 1.6 Hz, 4H), 5.19 – 4.98 (m, 1H), 3.82 (t, *J* = 8.0 Hz, 1H), 3.77 (s, 3H), 2.15 – 1.99 (m, 2H), 1.65 (s, 7H), 1.19 (d, *J* = 6.4 Hz, 7H), 0.91 (t, *J* = 7.6 Hz, 3H). **¹³C NMR** (100 MHz, $CDCl_3$) δ 195.2, 173.2, 159.4, 158.1, 150.2, 136.4, 135.9, 132.0, 130.8, 130.1, 128.8, 127.7, 117.1, 113.9, 79.3, 69.3, 55.2, 52.4, 28.6, 25.4, 21.5, 12.7. HRMS (EI) calculated for $C_{30}H_{34}O_5$ $[M]^+$ 474.5970, found 474.5972.

Phenyl(4-(1-phenylbutyl) phenyl) methanone (23)

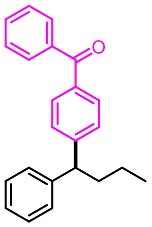

The title compound was prepared following the general procedure, purification by column chromatography on silica gel (petroleum ether/EtOAc = 100:1) yielded (77 mg, 82%) as a colorless oil. **¹H NMR** (400 MHz, $CDCl_3$) δ 7.83 – 7.69 (m, 4H), 7.59 – 7.52 (m, 1H), 7.48 – 7.42 (m, 2H), 7.37 – 7.32 (m, 2H), 7.32 – 7.23 (m, 4H), 7.22 – 7.16 (m, 1H), 3.99 (d, *J* = 8.0 Hz, 1H), 2.75 – 1.95 (m, 2H), 1.55 – 1.15 (m, 2H), 0.94 (t, *J* = 7.6 Hz, 3H). **¹³C NMR** (100 MHz, $CDCl_3$) δ 196.4, 150.4, 144.4, 137.8, 135.4, 132.2, 130.4, 130.0, 128.6, 128.2, 127.9, 127.8, 126.4, 51.2, 37.7, 21.1, 14.1. **MS (EI)** $[M]^+$ *m*/*z* 314.

4-(1-Phenylbutyl) benzaldehyde (24)

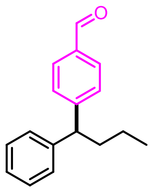

The title compound was prepared following the general procedure, purification by column chromatography on silica gel (petroleum ether/EtOAc = 50:1) yielded (48 mg, 68%) as a colorless oil. **^{1}H NMR** (400 MHz, $CDCl_3$) δ 9.95 (s, 1H), 7.85 – 7.73 (m, 2H), 7.40 (d, *J* = 8.0 Hz, 2H), 7.34 – 7.15 (m, 5H), 3.99 (t, *J* = 8.0 Hz, 1H), 2.13 – 1.99 (m, 2H), 1.40 – 1.20 (m, 2H), 0.93 (t, *J* = 7.6 Hz, 3H). **^{13}C NMR** (100 MHz, $CDCl_3$) δ 192.0, 152.7, 144.0, 134.6, 130.0, 128.6, 128.6, 127.9, 126.5, 51.3, 37.6, 21.1, 14.1. **MS (EI)** $[M]^+$ *m*/*z* 238.

1-(3-(1-Phenylbutyl) phenyl) ethan-1-one (25)

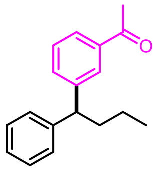

The title compound was prepared following the general procedure, purification by column chromatography on silica gel (petroleum ether/EtOAc = 50:1) yielded (57 mg, 76%) as a colorless oil. **^{1}H NMR** (400 MHz, $CDCl_3$) δ 7.87 (t, *J* = 2.0 Hz, 1H), 7.75 (dt, *J* = 8.0, 1.6 Hz, 1H), 7.44 (dt, *J* = 7.6, 1.6 Hz, 1H), 7.36 (t, *J* = 7.6 Hz, 1H), 7.31 – 7.14 (m, 5H), 3.97 (t, *J* = 8.0 Hz, 1H), 2.58 (s, 3H), 2.08 – 2.02 (m, 2H), 1.48 – 1.17 (m, 2H), 0.93 (t, *J* = 7.6 Hz, 3H); **^{13}C NMR** (100 MHz, CDCl3) δ 198.4, 146.0, 144.6, 137.3, 132.8, 128.7, 128.5, 127.8, 127.5, 126.4, 126.3, 51.0, 37.8, 26.7, 21.1, 14.1. **MS (EI)** $[M]^+$ *m*/*z* 252.

3-(1-Phenylbutyl) quinoline (26)

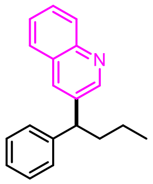

The title compound was prepared following the general procedure, purification by column chromatography on silica gel (petroleum ether/EtOAc = 20:1) yielded (42 mg, 54%) as a colorless oil. **^{1}H NMR** (400 MHz, $CDCl_3$) δ 8.81 (d, *J* = 2.3 Hz, 1H), 8.07 (d, *J* = 8.8 Hz, 1H), 7.97 (d, *J* = 2.4 Hz, 1H), 7.78 (dd, *J* = 8.0, 1.6 Hz, 1H), 7.72 – 7.62 (m, 1H), 7.56 – 7.48 (m, 1H), 7.34 – 7.25

(m, 4H), 7.23 – 7.17 (m, 1H), 4.13 (t, J = 8.0 Hz, 1H), 2.23 – 2.10 (m, 2H), 1.43 – 1.29 (m, 2H), 0.96 (t, J = 7.2 Hz, 3H). **^{13}C NMR** (175 MHz, $CDCl_3$) δ 151.8, 146.8, 143.9, 138.0, 133.6, 129.0, 128.9, 128.7, 128.2, 128.0, 127.6, 126.7, 126.6, 48.8, 37.6, 21.1, 14.1. **MS (EI)** $[M]^+$ *m/z* 261.

1-(Tert-butyl)-4-(1-(4-methoxyphenyl) butyl) benzene (27)

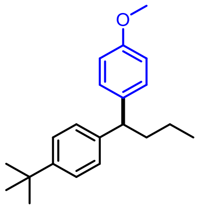

The title compound was prepared following the general procedure, purification by column chromatography on silica gel (petroleum ether/EtOAc = 100:1) yielded (73 mg, 82%) as a colorless oil. **^{1}H NMR** (400 MHz, $CDCl_3$) δ 7.29 – 7.25 (m, 2H), 7.20 – 7.10 (m, 4H), 6.91 – 6.77 (m, 2H), 3.82 (t, J = 8.0 Hz, 1H), 3.75 (s, 3H), 2.10 – 1.88 (m, 2H), 1.35 – 1.20 (m, 11H), 0.91 (t, J = 7.2 Hz, 3H). **^{13}C NMR** (100 MHz, $CDCl_3$) δ 157.8, 148.5, 142.8, 137.7, 128.8, 127.3, 125.2, 113.7, 55.2, 49.8, 38.3, 34.3, 31.4, 21.3, 14.1. **MS (EI)** $[M]^+$ *m/z* 296.

4-(1-(4-Methoxyphenyl) butyl)-1,1'-biphenyl (28)

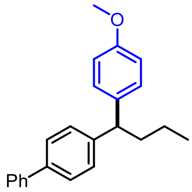

The title compound was prepared following the general procedure, purification by column chromatography on silica gel (petroleum ether/EtOAc = 100:1) yielded (78 mg, 82%) as a colorless oil. **^{1}H NMR** (400 MHz, $CDCl_3$) δ 7.58 – 7.53 (m, 2H), 7.49 (d, J = 8.4 Hz, 2H), 7.39 (d, J = 7.2 Hz, 2H), 7.33 – 7.25 (m, 3H), 7.21 – 7.14 (m, 2H), 6.88 – 6.80 (m, 2H), 3.89 (d, J = 8.0 Hz, 1H), 3.75 (s, 3H), 2.17 – 1.91 (m, 2H), 1.41 – 1.21 (m, 2H), 0.93 (t, J = 7.6 Hz, 3H). **^{13}C NMR** (100 MHz, $CDCl_3$) δ 157.9, 144.9, 141.0, 138.8, 137.4, 128.8, 128.7, 128.2, 127.1, 127.0, 127.0, 113.8, 55.2, 49.9, 38.1, 21.2, 14.1. **MS (EI)** $[M]^+$ *m/z* 316.

4-(1-(4-Methoxyphenyl) butyl) phenol (29)

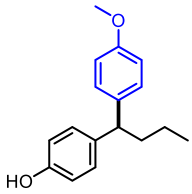

The title compound was prepared following the general procedure, purification by column chromatography on silica gel (petroleum ether/EtOAc = 20:1) yielded (57 mg, 74%) as a colorless oil. **^{1}H NMR** (400 MHz, $CDCl_3$) δ 7.12 (d, *J* = 8.4 Hz, 1H), 7.06 (d, *J* = 8.4 Hz, 1H), 6.81 (d, *J* = 8.8 Hz, 1H), 6.71 (d, *J* = 8.4 Hz, 1H), 5.34 (s, 1H), 3.78 (d, *J* = 8.0 Hz, 1H), 3.75 (s, 2H), 2.03 – 1.82 (m, 1H), 1.31 – 1.17 (m, 2H), 0.90 (t, *J* = 7.6 Hz, 2H). **^{13}C NMR** (100 MHz, $CDCl_3$) δ 157.6, 153.6, 138.0, 138.0, 128.8, 128.7, 115.2, 113.8, 55.3, 49.3, 38.3, 21.1, 14.1. **MS (EI)** $[M]^+$ *m/z* 256.

4-(4-(1-(4-Methoxyphenyl) butyl) phenyl) morpholine (30)

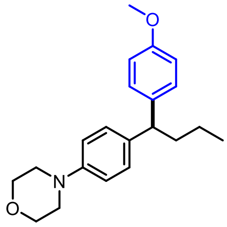

The title compound was prepared following the general procedure, purification by column chromatography on silica gel (petroleum ether/EtOAc = 25:1) yielded (77 mg, 79%) as a colorless oil. **^{1}H NMR** (400 MHz, $CDCl_3$) δ 7.17 – 7.09 (m, 4H), 6.92 – 6.73 (m, 4H), 3.87 – 3.76 (m, 5H), 3.75 (s, 3H), 3.33 – 2.76 (m, 4H), 2.03 – 1.87 (m, 2H), 1.35 – 1.20 (m, 2H), 0.90 (t, *J* = 7.6 Hz, 3H). **^{13}C NMR** (101 MHz, $CDCl_3$) δ 157.7, 149.3, 138.0, 137.4, 128.7, 128.4, 115.7, 113.7, 67.0, 55.2, 49.5, 49.3, 38.2, 21.2, 14.1. **MS (EI)** $[M]^+$ *m/z* 325.

1-Fluoro-4-(1-(4-methoxyphenyl) butyl) benzene (31)

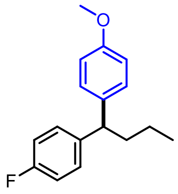

The title compound was prepared following the general procedure, purification by column chromatography on silica gel (petroleum ether/EtOAc = 100:1) yielded (49 mg, 64%) as a colorless oil. **^{1}H NMR** (400 MHz, $CDCl_3$) δ 7.21 – 7.08 (m, 4H), 6.99 – 6.91 (m, 2H), 6.87 – 6.78 (m, 2H), 3.84 (t, *J* = 8.0 Hz, 1H), 3.76 (s, 3H), 2.06 – 1.88 (m, 2H), 1.37 – 1.17 (m, 2H), 0.91 (t, *J* = 7.2 Hz,

3H). **^{13}C NMR** (100 MHz, $CDCl_3$) δ 161.2 (d, *J* = 242.2 Hz), 158.0, 141.5 (d, *J* = 3.2 Hz) , 137.4, 129.1 (d, *J* = 7.8Hz), 128.7, 115.1 (d, *J* = 20.8 Hz), 113.9, 55.3, 49.5, 38.3, 21.2, 14.1. **^{19}F NMR** (376 MHz, $CDCl_3$) δ -117.7. **MS (EI)** $[M]^+$ *m*/*z* 258.

2-(1-(4-Methoxyphenyl) butyl) naphthalene (32)

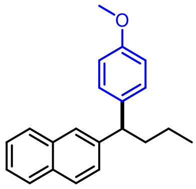

The title compound was prepared following the general procedure, purification by column chromatography on silica gel (petroleum ether/EtOAc = 100:1) yielded (72 mg, 83%) as a colorless oil. **^{1}H NMR** (400 MHz, $CDCl_3$) δ 7.83 – 7.65 (m, 4H), 7.48 – 7.34 (m, 2H), 7.31 (dd, *J* = 8.4, 1.6 Hz, 1H), 7.22 – 7.14 (m, 2H), 6.89 – 6.77 (m, 2H), 4.01 (d, *J* = 8.0 Hz, 1H), 3.73 (s, 3H), 2.17 – 1.98 (m, 2H), 1.38 – 1.23 (m, 2H), 0.93 (t, *J* = 7.6 Hz, 3H). **^{13}C NMR** (100 MHz, $CDCl_3$) δ 157.9, 143.2, 137.3, 133.6, 132.1, 128.9, 128.0, 127.7, 127.6, 126.8, 125.9, 125.7, 125.3, 113.8, 55.2, 50.2, 37.9, 21.2, 14.2. **MS (EI)** $[M]^+$ *m*/*z* 290.

2-(1-(4-Methoxyphenyl) propyl) phenol (33)

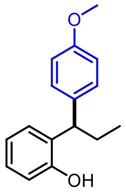

The title compound was prepared following the general procedure, purification by column chromatography on silica gel (petroleum ether/EtOAc = 30:1) yielded (46 mg, 63%) as a colorless oil. **^{1}H NMR** (400 MHz, $CDCl_3$) δ 7.28 – 7.24 (m, 1H), 7.19 – 7.14 (m, 2H), 7.09 (td, *J* = 7.6, 1.6 Hz, 1H), 6.93 (td, *J* = 7.6, 1.2 Hz, 1H), 6.85 – 6.79 (m, 2H), 6.73 (dd, *J* = 8.0, 1.6 Hz, 1H), 4.72 (s, 1H, OH), 4.00 (d, *J* = 7.2 Hz, 1H), 3.76 (s, 2H), 2.17 – 1.90 (m, 2H), 0.91 (t, *J* = 7.3 Hz, 3H). **^{13}C NMR** (100 MHz, $CDCl_3$) δ 158.1, 153.5, 136.1, 131.3, 129.0, 127.9, 127.3, 120.8, 116.0, 114.0, 55.2, 45.5, 27.8, 12.7. **MS (EI)** $[M]^+$ *m*/*z* 242.

1-(1-(4-Methoxyphenyl) propyl) naphthalene (34)

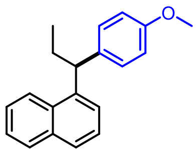

The title compound was prepared following the general procedure, purification by column chromatography on silica gel (petroleum ether/EtOAc = 100:1) yielded (65 mg, 79%) as a white solid. **^{1}H NMR** (400 MHz, $CDCl_3$) δ 8.15 – 8.08 (m, 1H), 7.84 – 7.79 (m, 1H), 7.75 – 7.67 (m, 1H), 7.49 – 7.38 (m, 4H), 7.18 (d, J = 8.8 Hz, 2H), 6.78 (d, J = 8.4 Hz, 2H), 4.55 (d, J = 7.6 Hz, 1H), 3.73 (s, 3H), 2.61 – 1.83 (m, 2H), 0.98 (t, J = 7.2 Hz, 3H). **^{13}C NMR** (100 MHz, $CDCl_3$) δ 157.8, 140.9, 137.2, 134.1, 132.0, 129.1, 128.8, 126.8, 125.8, 125.4, 125.3, 124.1, 123.8, 113.7, 55.2, 47.4, 29.4, 13.0. **MS (EI)** $[M]^+$ *m/z* 276.

1-Methoxy-4-(1-phenylpropyl) benzene (35)

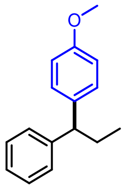

The title compound was prepared following the general procedure, purification by column chromatography on silica gel (petroleum ether/EtOAc = 100:1) yielded (54 mg, 80%) as a colorless oil. **^{1}H NMR** (400 MHz, $CDCl_3$) δ 7.30 – 7.19 (m, 4H), 7.17 – 7.09 (m, 3H), 6.88 – 6.78 (m, 2H), 3.78 – 3.70 (m, 4H), 2.50 – 1.99 (m, 2H), 0.88 (t, J = 7.2 Hz, 3H). **^{13}C NMR** (100 MHz, $CDCl_3$) δ 157.8, 145.6, 137.4, 128.8, 128.4, 127.8, 127.8, 125.9, 114.2, 113.7, 77.4, 77.1, 76.7, 55.2, 52.4, 28.8, 12.8. **MS (EI)** $[M]^+$ *m/z* 226.

1-Methoxy-4-(1-phenylpentyl) benzene (36)

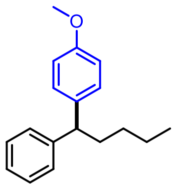

The title compound was prepared following the general procedure, purification by column chromatography on silica gel (petroleum ether/EtOAc = 100:1) yielded (59 mg, 78%) as a colorless oil. **^{1}H NMR** (400 MHz, $CDCl_3$) δ 7.31 – 7.20 (m, 4H), 7.17 – 7.12 (m, 3H), 6.88 – 6.78 (m, 2H), 3.83 (t, J = 8.0 Hz, 1H), 3.76 (s, 3H), 2.09 – 1.93 (m, 2H), 1.39 – 1.28 (m, 2H), 1.28 – 1.19 (m, 2H),

0.86 (t, *J* = 7.6 Hz, 3H). **^{13}C NMR** (101 MHz, $CDCl_3$) δ 157.8, 145.8, 137.6, 128.8, 128.4, 127.8, 125.9, 113.8, 55.2, 50.5, 35.7, 30.3, 22.8, 14.1. **MS (EI)** $[M]^+$ *m/z* 254.

1-Methoxy-4-(1-phenylhexyl) benzene (37)

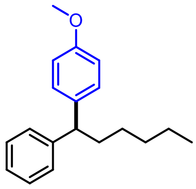

The title compound was prepared following the general procedure, purification by column chromatography on silica gel (petroleum ether/EtOAc = 100:1) yielded (59 mg, 73%) as a colorless oil. **^{1}H NMR** (400 MHz, $CDCl_3$) δ 7.30 – 7.19 (m, 4H), 7.17 – 7.12 (m, 3H), 6.86 – 6.77 (m, 2H), 3.83 (t, *J* = 7.8 Hz, 1H), 3.76 (s, 3H), 2.04 – 1.94 (m, 2H), 1.37 – 1.18 (m, 6H), 0.84 (t, *J* = 6.8 Hz, 3H). **^{13}C NMR** (100 MHz, $CDCl_3$) δ 157.8, 145.8, 137.6, 128.8, 128.4, 127.8, 125.9, 113.8, 55.2, 50.5, 35.9, 31.9, 27.8, 22.6, 14.1. **MS (EI)** $[M]^+$ *m/z* 268.

(4-(1-Phenylpropyl) phenyl) methanol (38)

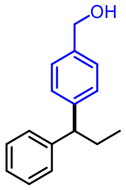

The title compound was prepared following the gram scale reaction, purification by column chromatography on silica gel (petroleum ether/EtOAc = 5:1) yielded (1.1 g, 80%) as a colorless oil. **^{1}H NMR** (400 MHz, $CDCl_3$) δ 7.34 – 7.20 (m, 8H), 7.18 – 7.12 (m, 1H), 4.62 (s, 2H), 3.79 (t, *J* = 8.0 Hz, 1H), 2.07 (p, *J* = 7.2 Hz, 2H), 0.89 (t, *J* = 7.2 Hz, 3H). **^{13}C NMR** (100 MHz, $CDCl_3$) δ 145.1, 144.8, 138.6, 128.4, 128.2, 127.9, 127.3, 126.1, 65.3, 53.0, 28.6, 12.8. **MS (EI)** $[M]^+$ *m/z* 226.

17-Hydroxy-13-methyl-17-(1-(4-(-1-phenylpropyl)benzyl)-1*H*-1,2,3-triazol-4-yl)-1,2,6,7,8,9,10,11,12,13,14,15,16,17-tetradecahydro-3*H*-cyclopenta[*a*]phenanthren-3-one (40)

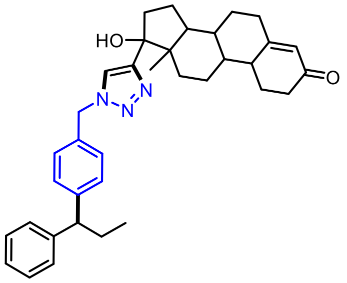

The title compound was prepared following the general procedure, purification by column chromatography on silica gel (petroleum ether/EtOAc = 3:1) yielded (110 mg, 80%) as a white solid. **^{1}H NMR** (400 MHz, $CDCl_3$) δ 7.36 – 7.12 (m, 10H), 5.79 (s, 1H), 5.56 – 5.36 (m, 2H), 3.78 (t, *J* = 7.8 Hz, 1H), 2.89 (s, 1H), 2.49 – 2.41 (m, 1H), 2.40 – 2.23 (m, 3H), 2.23 – 2.15 (m, 2H), 2.11 – 1.98 (m, 4H), 1.94 – 1.78 (m, 2H), 1.74 – 1.64 (m, 1H), 1.54 – 1.35 (m, 5H), 1.31 – 1.12 (m, 2H), 1.05 (s, 4H), 0.87 (t, *J* = 7.3 Hz, 3H), 0.72 – 0.58 (m, 1H), 0.48 (td, *J* = 13.1, 4.3 Hz, 1H). **^{13}C NMR** (100 MHz, $CDCl_3$) δ 199.9, 166.8, 153.9, 145.9, 144.6, 132.4, 128.6, 128.5, 128.0, 127.8, 126.3, 124.5, 121.1, 82.2, 77.3, 53.8, 52.9, 48.9, 48.2, 47.1, 42.5, 41.1, 37.7, 36.5, 35.5, 32.6, 30.7, 28.5, 26.5, 26.1, 23.5, 14.3, 12.7. HRMS (EI) calculated for $C_{24}H_{23}N_3$ $[M]^+$ 549.3355, found 549.3354.

4-Phenyl-1-(4-(1-phenylpropyl) benzyl)-1*H*-1,2,3-triazole (41)

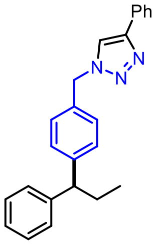

The title compound was prepared following the general procedure, purification by column chromatography on silica gel (petroleum ether/EtOAc = 4:1) yielded (62 mg, 88%) as a white solid. **^{1}H NMR** (400 MHz, $CDCl_3$) δ 7.80 – 7.76 (m, 2H), 7.62 (s, 1H), 7.45 – 7.34 (m, 2H), 7.31 – 7.11 (m, 9H), 5.48 (s, 2H), 3.78 (t, *J* = 8.0 Hz, 1H), 2.22 – 1.76 (m, 2H), 0.88 (t, *J* = 7.6 Hz, 3H). **^{13}C NMR** (100 MHz, $CDCl_3$) δ 148.1, 146.0, 144.6, 132.3, 130.6, 128.8, 128.6, 128.5, 128.2, 128.1, 127.9, 126.2, 125.7, 119.5, 53.9, 53.0, 28.5, 12.7. HRMS (EI) calculated for $C_{24}H_{23}N_3$ $[M]^+$ 353.4690, found 353.4694.

Methyl-3-(4-methoxyphenyl)-3-phenylpropanoate (42)

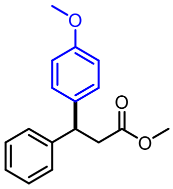

The title compound was prepared following the general procedure, purification by column chromatography on silica gel (petroleum ether/EtOAc = 30:1) yielded (36 mg, 45%) as a colorless oil. **^{1}H NMR** (400 MHz, $CDCl_3$) δ 7.26 (d, *J* = 7.4 Hz, 1H), 7.23 – 7.08 (m, 2H), 6.87 – 6.79 (m, 1H), 4.51 (t, *J* = 8.0 Hz, 0H), 3.76 (s, 1H), 3.58 (s, 1H), 3.03 (d, *J* = 8.0 Hz, 1H). **^{13}C NMR** (100 MHz, $CDCl_3$) δ 172.4, 158.2, 143.9, 135.6, 128.6, 128.6, 127.6, 126.5, 114.0, 55.2, 51.7, 46.2, 40.8. **MS (EI)** $[M]^+$ *m*/*z* 270.

3-(4-Methoxyphenyl)-3-phenylpropan-1-ol (43)

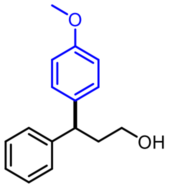

The title compound was prepared following the general procedure, purification by column chromatography on silica gel (petroleum ether/EtOAc = 5:1) yielded (43 mg, 60%) as a white solid. **^{1}H NMR** (400 MHz, $CDCl_3$) δ 7.36 – 7.22 (m, 4H), 7.19 – 7.14 (m, 3H), 6.82 (d, *J* = 8.0 Hz, 2H), 4.08 (t, *J* = 8.0 Hz, 1H), 3.76 (s, 3H), 3.60 (t, *J* = 6.4 Hz, 2H), 2.28 (q, *J* = 6.8 Hz, 2H). **^{13}C NMR** (100 MHz, $CDCl_3$) δ 158.0, 144.9, 136.6, 128.8, 128.5, 127.8, 126.2, 113.9, 61.2, 55.3, 46.5, 38.4. HRMS (EI) calculated for $C_{30}H_{34}O_5$ $[M]^+$ 242.3180, found 242.3181.

1-(4-(1-Phenylpropyl) phenyl) ethan-1-one (45)

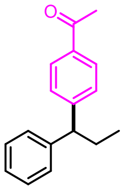

The title compound was prepared following the general procedure, purification by column chromatography on silica gel (petroleum ether/EtOAc = 100:1) yielded (53 mg, 75%) as a colorless oil. **^{1}H NMR (**400 MHz, $CDCl_3$) δ 7.95 – 7.83 (m, 1H), 7.37 – 7.16 (m, 4H), 3.85 (d, *J* = 8.0 Hz, 1H), 2.55 (s, 1H), 2.19 – 2.00 (m, 1H), 0.90 (t, *J* = 7.2 Hz, 2H). **^{13}C NMR** (100 MHz, $CDCl_3$) δ 197.8, 150.9, 144.1, 135.2, 128.6, 128.6, 128.1, 127.9, 126.4, 53.2, 28.3, 26.6, 12.7. **MS (EI)** $[M]^+$

m/*z* 238.

2-(4-Methoxyphenyl)-1-phenylpyrrolidine (46)

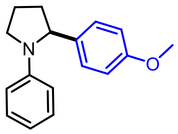

The title compound was prepared following the general procedure, purification by column chromatography on silica gel (petroleum ether/EtOAc = 50:1) yielded (57 mg, 75%) as a colorless oil. **^{1}H NMR** (400 MHz, $CDCl_3$) δ 7.18 – 7.09 (m, 4H), 6.91 – 6.79 (m, 2H), 6.62 (dd, *J* = 7.2, 1.2 Hz, 1H), 6.54 – 6.43 (m, 2H), 4.67 (dd, *J* = 8.4, 2.4 Hz, 1H), 3.76 (s, 3H), 3.73 – 3.62 (m, 1H), 3.37 (td, *J* = 8.8, 6.8 Hz, 1H), 2.41 – 2.27 (m, 1H), 2.08 – 1.81 (m, 2H). **^{13}C NMR** (100 MHz, $CDCl_3$) δ 158.3, 147.2, 136.6, 129.0, 126.9, 115.7, 113.8, 112.3, 62.3, 55.2, 49.0, 36.2, 23.1. **MS (EI)** $[M]^+$ *m*/*z* 253.

1-(4-(Tetrahydrofuran-2-yl) phenyl) ethan-1-one (47)

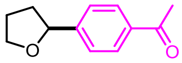

The title compound was prepared following the general procedure, purification by column chromatography on silica gel (petroleum ether/EtOAc = 50:1) yielded (28 mg, 49%) as a colorless oil. **^{1}H NMR** (400 MHz, $CDCl_3$) δ 7.93 (d, *J* = 8.4 Hz, 2H), 7.42 (d, *J* = 8.4 Hz, 2H), 4.95 (t, *J* = 7.2 Hz, 1H), 4.19 – 4.07 (m, 1H), 4.01 – 3.92 (m, 1H), 2.60 (s, 3H), 2.44 – 2.25 (m, 1H), 2.13 – 1.91 (m, 2H), 1.87 – 1.72 (m, 1H). ^{13}C NMR (100 MHz, $CDCl_3$) δ 197.9, 149.2, 136.1, 128.5, 125.6, 80.2, 68.9, 34.7, 26.7, 26.0. **MS (EI)** $[M]^+$ *m*/*z* 190.

9. Cyclic voltammetry[5]

Cyclic voltammograms were recorded with Admiral Squidsta Plus workstation potentiostat at room temperature in DMAc. $^{n}Bu_4NBr$ (0.1 M) was used as the supporting electrolyte, and a standard platinum electrode was used as the working electrode. The auxiliary electrode was a glassn carbon electrode. All potentials are referenced against the Ag/AgCl redox couple. The scan rate was 100 mV/s.

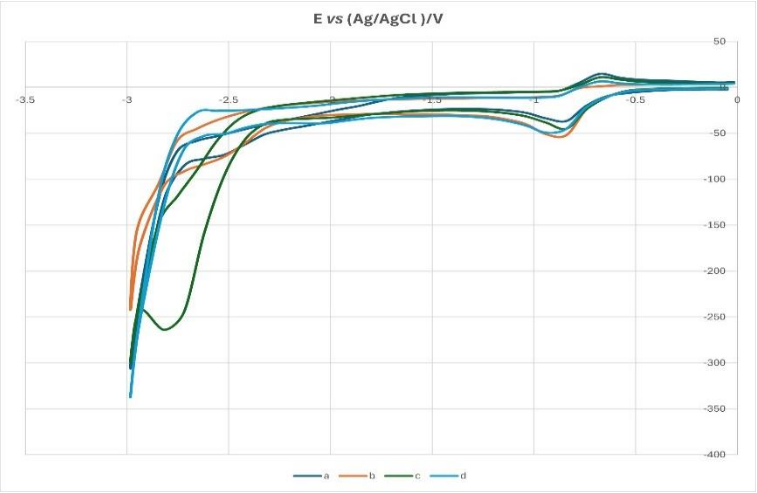

Figure S3: Cyclic voltammograms recorded on a Pt electrode at 100 mV/s in: (a) DMA containing 0.1 M of $^{n}Bu_4NBr$; (b) solution (a) with 5.0 mM of 1-bromopropane added; c) solution (a) with 5.0 mM of 4-bromoanisole added; d) solution (a) with 5.0 mM of 4-phenylbutene added.

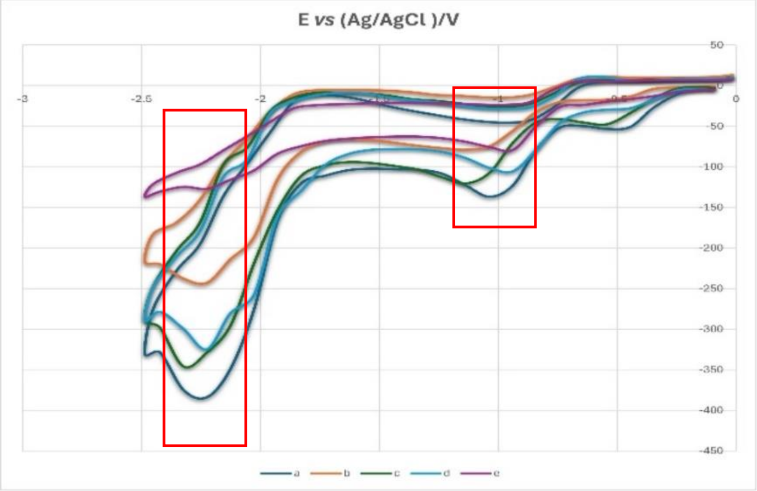

Figure S4: Cyclic voltammograms recorded on DMA containing 0.1 M of $^{n}Bu_4NBr$, a Pt electrode at 100 mV/s in: (a) with 7.5 mM of $Ni(ClO_4)_2 \cdot 6H_2O$ and **L1** added; (b) with 7.5 mM of $NiCl_2$ and **L1** added; (c) with 7.5 mM of $NiBr_2 \cdot DME$ and **L1** added; (d) with 7.5 mM of $Ni(OTf)_2$ and **L1** added; (e)with 7.5 mM of NiI_2 and **L1** added.

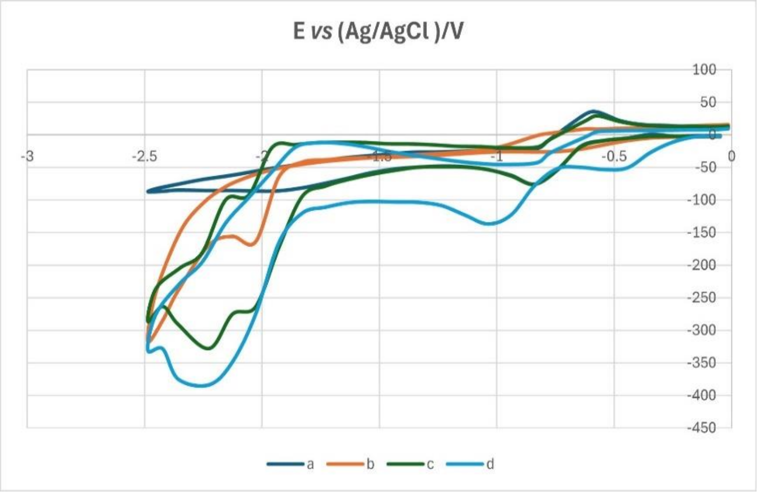

Figure S5: Cyclic voltammograms recorded on a Pt electrode at 100 mV/s in: (a) DMA containing 0.1 M of ^{n}Bu$_4$NBr; (b) solution (a) with 7.5 mM of Ni(ClO$_4$)$_2$·6H$_2$O added; c) solution (a) with 7.5 mM of **L1** added; d) solution (a) with 7.5 mM of Ni(ClO$_4$)$_2$·6H$_2$O and 7.5 mM of **L1** added.

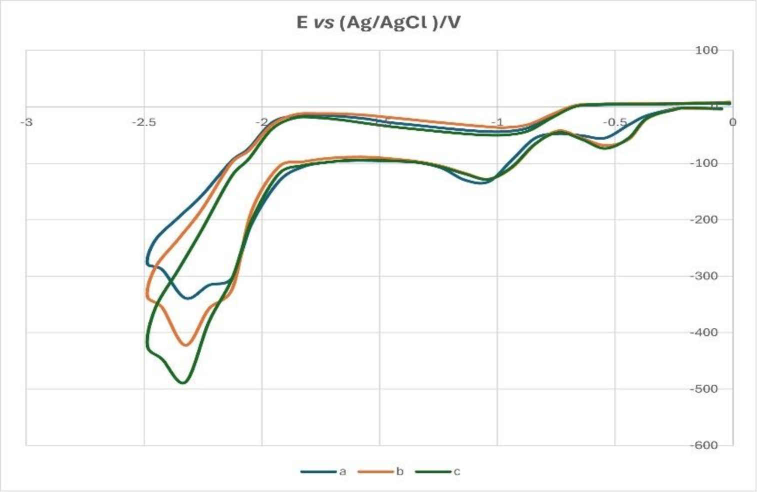

Figure S6: Cyclic voltammograms recorded on DMA containing 0.1 M of ^{n}Bu$_4$NBr, a Pt electrode at 100 mV/s in: (a) with 5.0 mM of Ni(ClO$_4$)$_2$·6H$_2$O and **L1** added; (b) solution (a) with 5.0 mM of 4-bromoanisole added; c) solution (a) with 10.0 mM of 4-bromoanisole added.

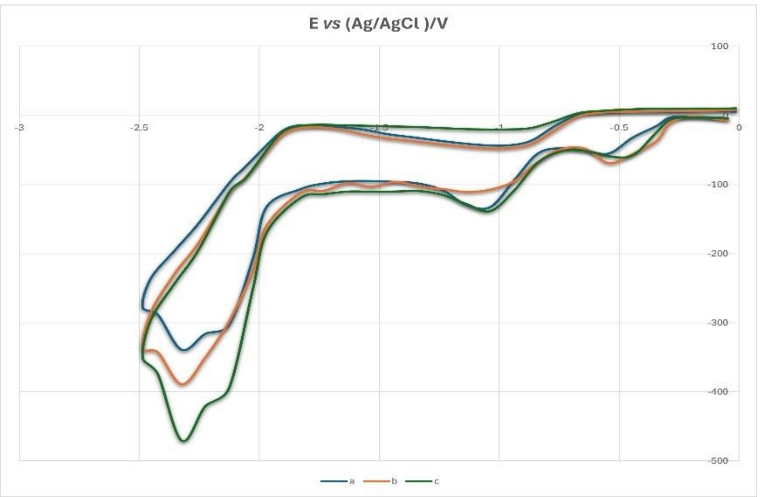

Figure S7: Cyclic voltammograms recorded on DMA containing 0.1 M of $^{n}Bu_4NBr$, a Pt electrode at 100 mV/s in: (a) with 5.0 mM of $Ni(ClO_4)_2 \cdot 6H_2O$ and **L1** added; (b) solution (a) with 5.0 mM of 1-bromopropane added; (c) solution (a) with 10.0 mM of 1-bromopropane added.

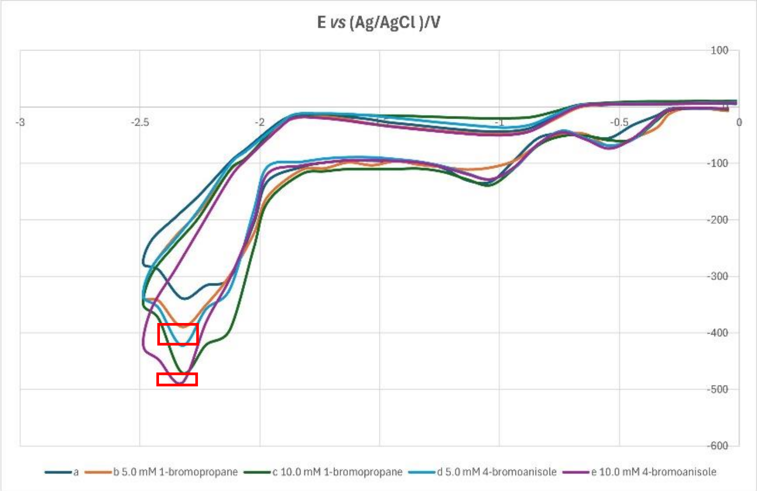

Figure S8: Cyclic voltammograms recorded on DMA containing 0.1 M of $^{n}Bu_4NBr$, a Pt electrode at 100 mV/s in: (a) with 5.0 mM of $Ni(ClO_4)_2 \cdot 6H_2O$ and **L1** added; (b) solution (a) with 5.0 mM of 1-bromopropane added; (c) solution (a) with 10.0 mM of 1-bromopropane added; (d)solution (a) with 5.0 mM of 4-bromoanisole added; (e) solution (a) with 10.0 mM of 4-bromoanisole added

10. Reference

[1] F. Q. Zhao, X. W. Gu, R. Franke, X. F. Wu, *Angew. Chem. Int. Ed.*, 2022, 61, e202214812.

[2] N. I. Saper, A. Ohgi, D. W. Small, K. Semba, Y. Nakao, J. F. Hartwig, *Nat. Chem.*, 2020, 12, 276.

[3] Ligand chose: (a) T. Morgas, J. Cornella, R. Martin, *J. Am. Chem. Soc.*, 2014, 136, 17702. (b) Y. Liu, J. Cornella, R. Martin, *J. Am. Chem. Soc.*, 2014, 136, 11212. (c) X. Qiang, M. Nakajima, R. Martin, *J. Am. Chem. Soc.*, 2015, 137, 8924. (d) X. Wang, M. Nakajima, E. Serrano, R. Martin, *J. Am. Chem. Soc.*, 2016, 138, 15531. (e) F. Chen, K. Chen, Y. Zhang, Y. He, Y. M. Wang, S. L. Zhu, *J. Am. Chem. Soc.*, 2017, 139, 13929.

[4] Y. Y. Weng, X. B. Xu. H. T. Chen. Y. Y. Zhang, X. F. Zhuo, *Angew. Chem. Int. Ed.*, 2022, 61, e202206308.

[5] (a) K. J. Jiao, D. Liu, H. X. Ma, H. Qiu, P. Fang, T. S. Mei, *Angew. Chem. Int. Ed.*, 2020, 59, 6520; (b) G. S. Kumar, A. Peshkov, A. Brzozowska, P. Nikolaienko, C. Zhu, M. Rueping, *Angew. Chem. Int. Ed.*, 2020, 59, 6513.

11. Spectra of compounds

^{1}H NMR spectrum of **S-9** ($CDCl_3$)

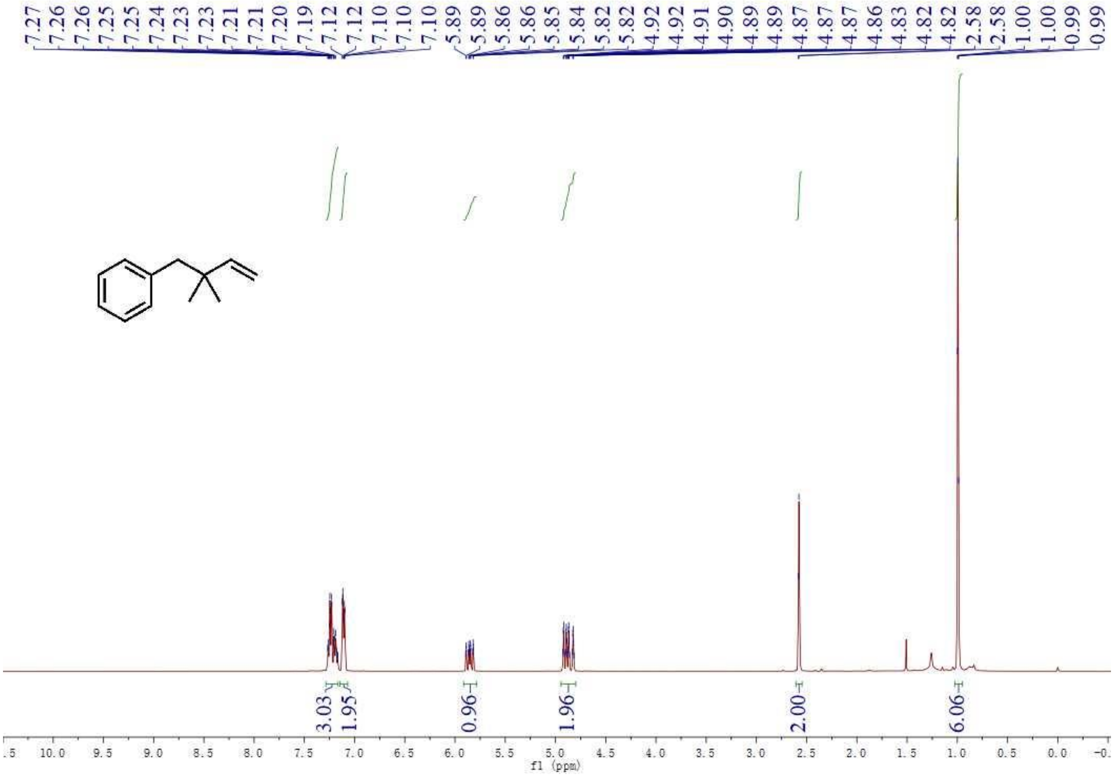

^{13}C NMR spectrum of **S-9** ($CDCl_3$)

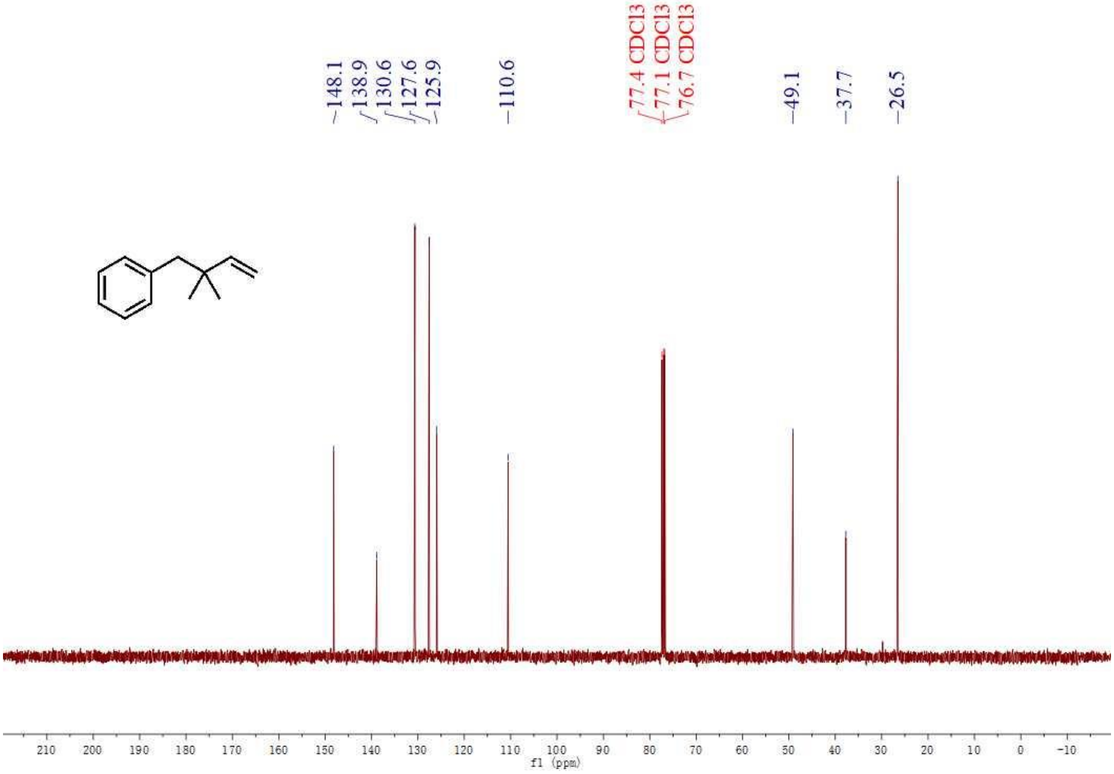

^{1}H NMR spectrum of **3** ($CDCl_3$)

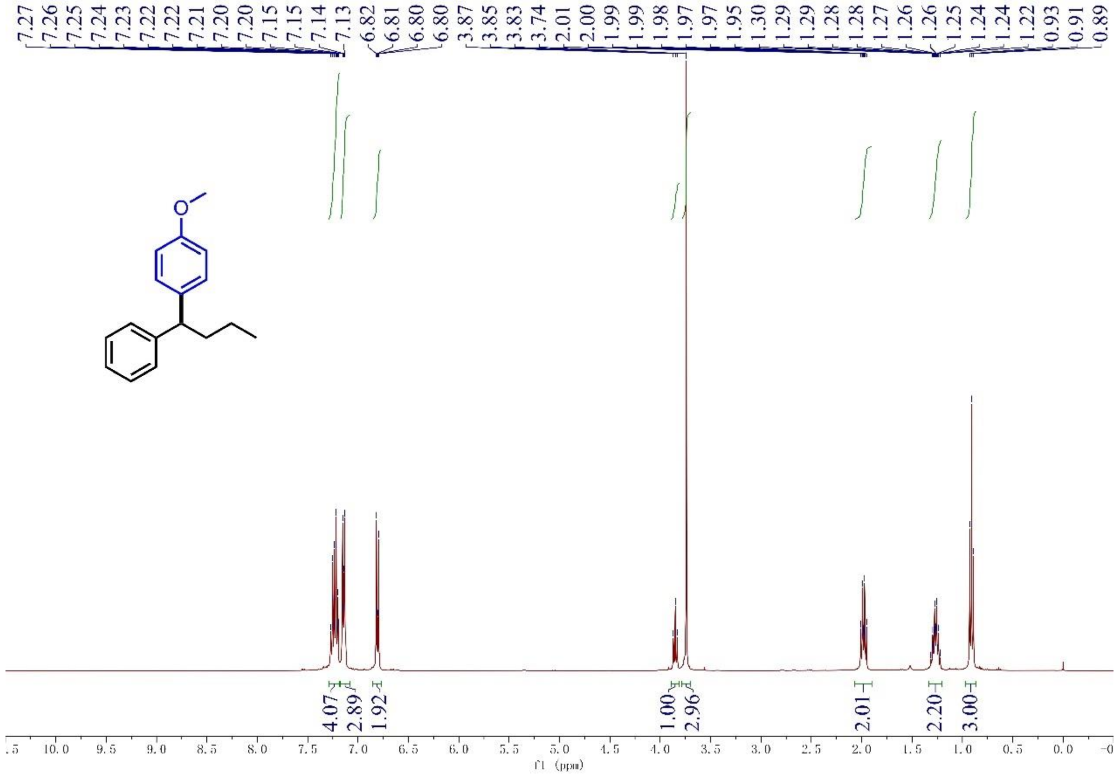

^{13}C NMR spectrum of **3** ($CDCl_3$)

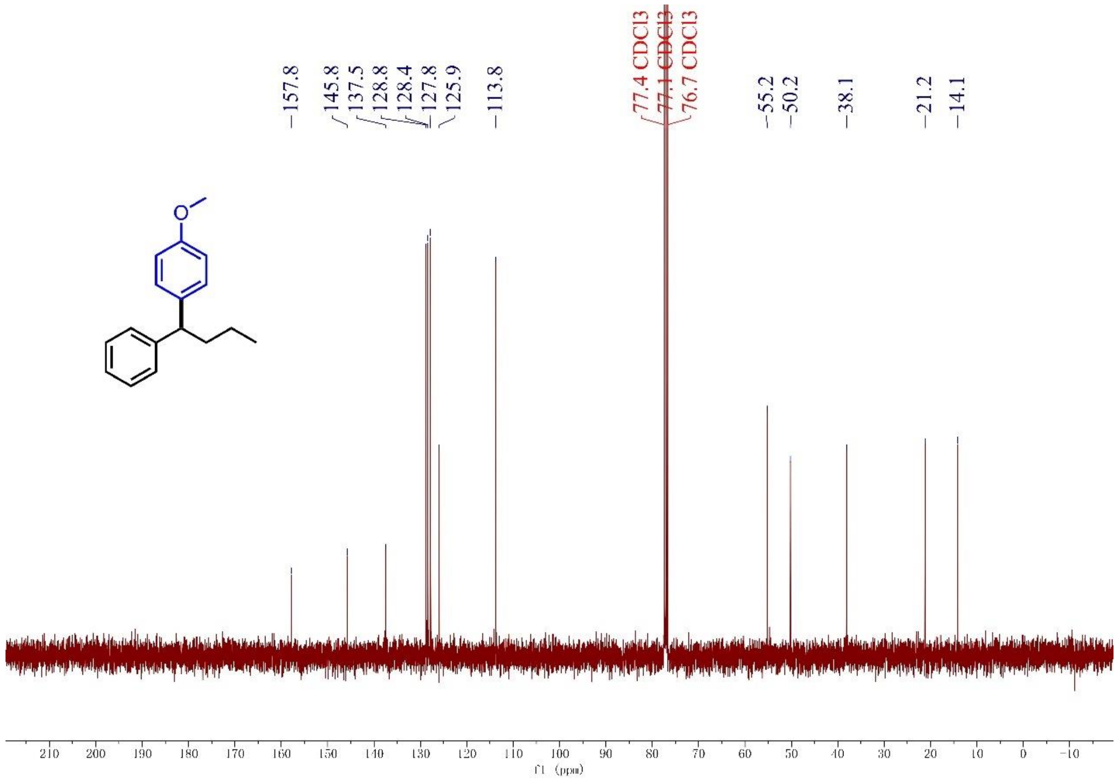

^{1}H NMR spectrum of **4** ($CDCl_3$)

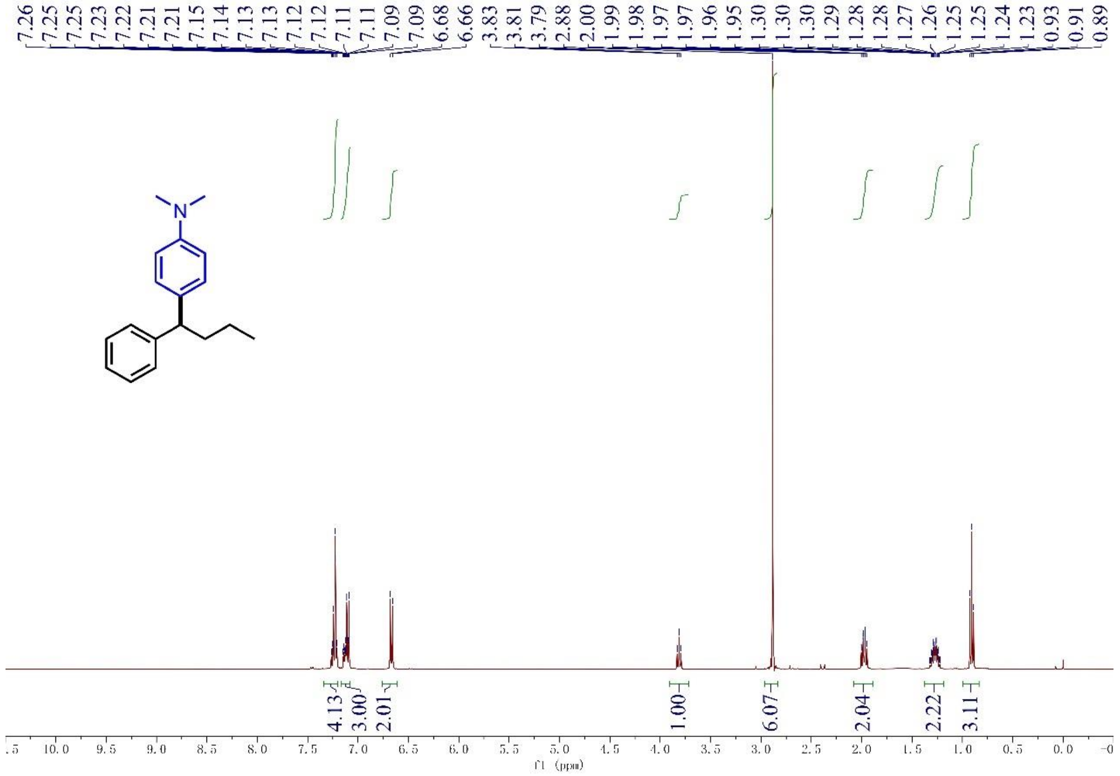

^{13}C NMR spectrum of **4** ($CDCl_3$)

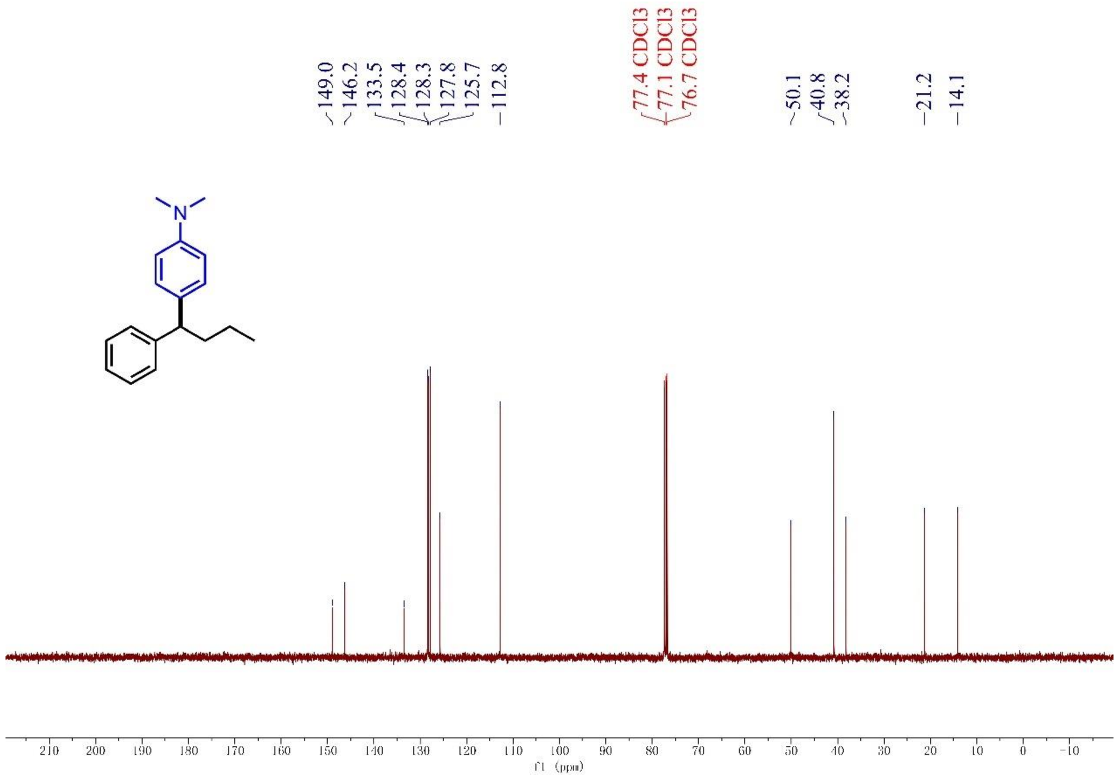

^{1}H NMR spectrum of **5** ($CDCl_3$)

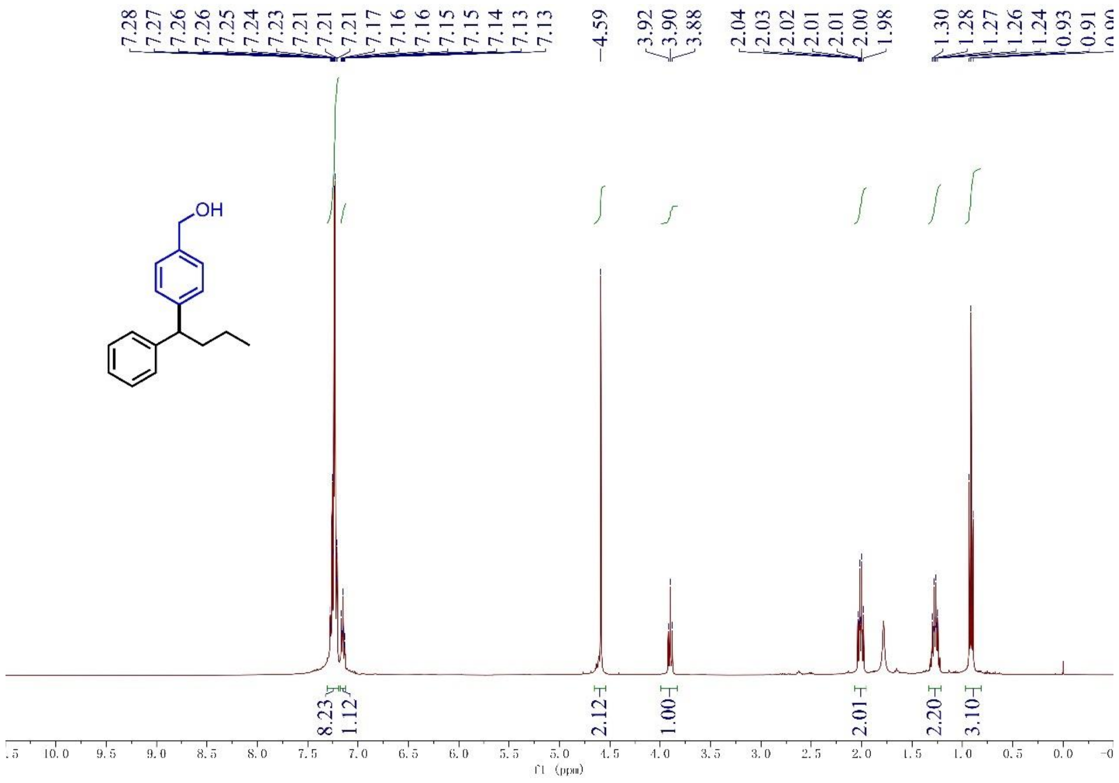

^{13}C NMR spectrum of **5** ($CDCl_3$)

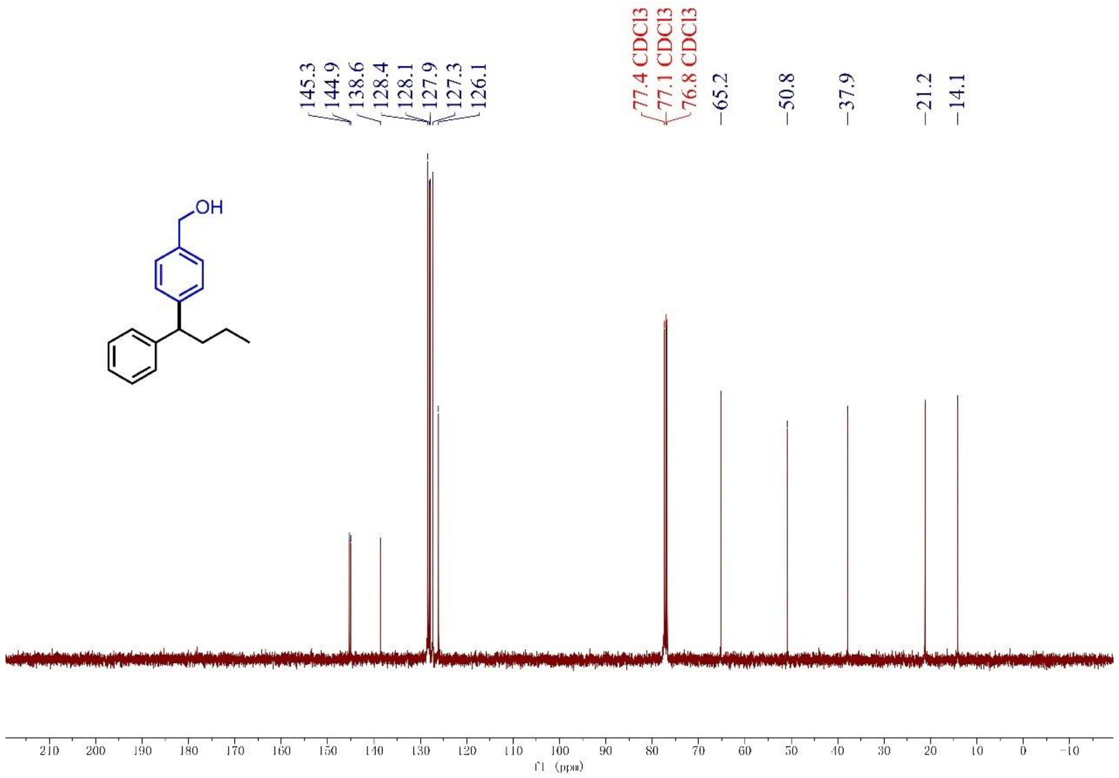

^{1}H NMR spectrum of **6** ($CDCl_3$)

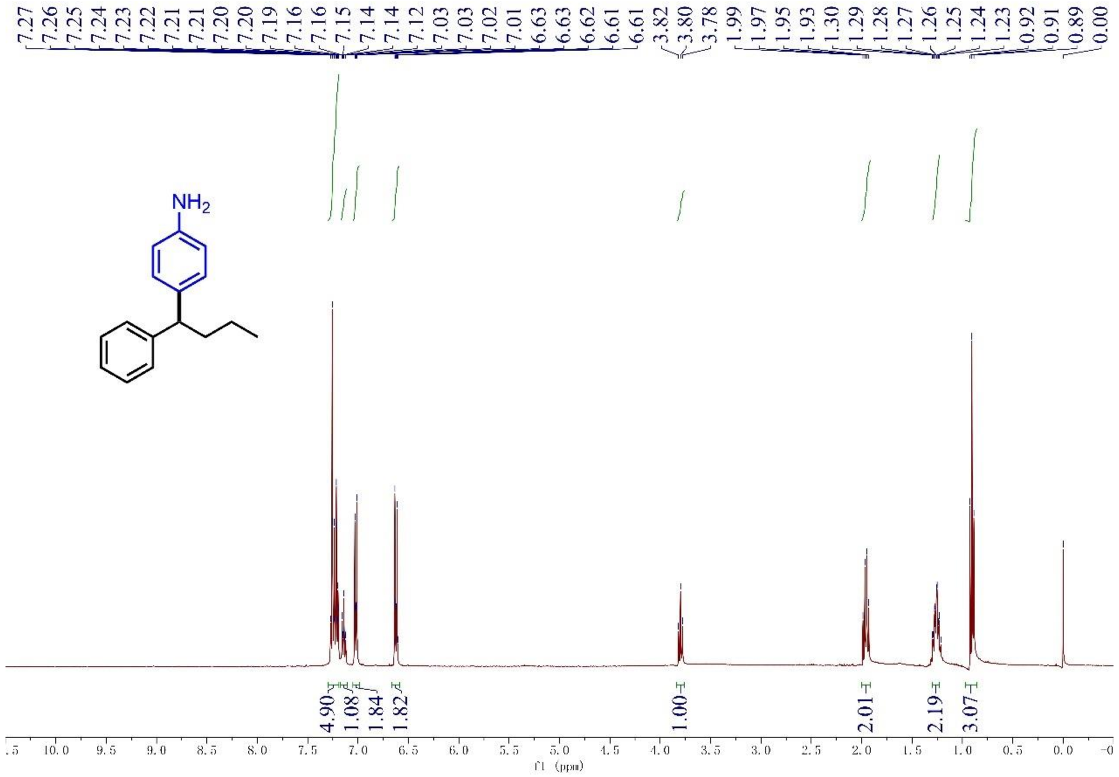

^{13}C NMR spectrum of **6** ($CDCl_3$)

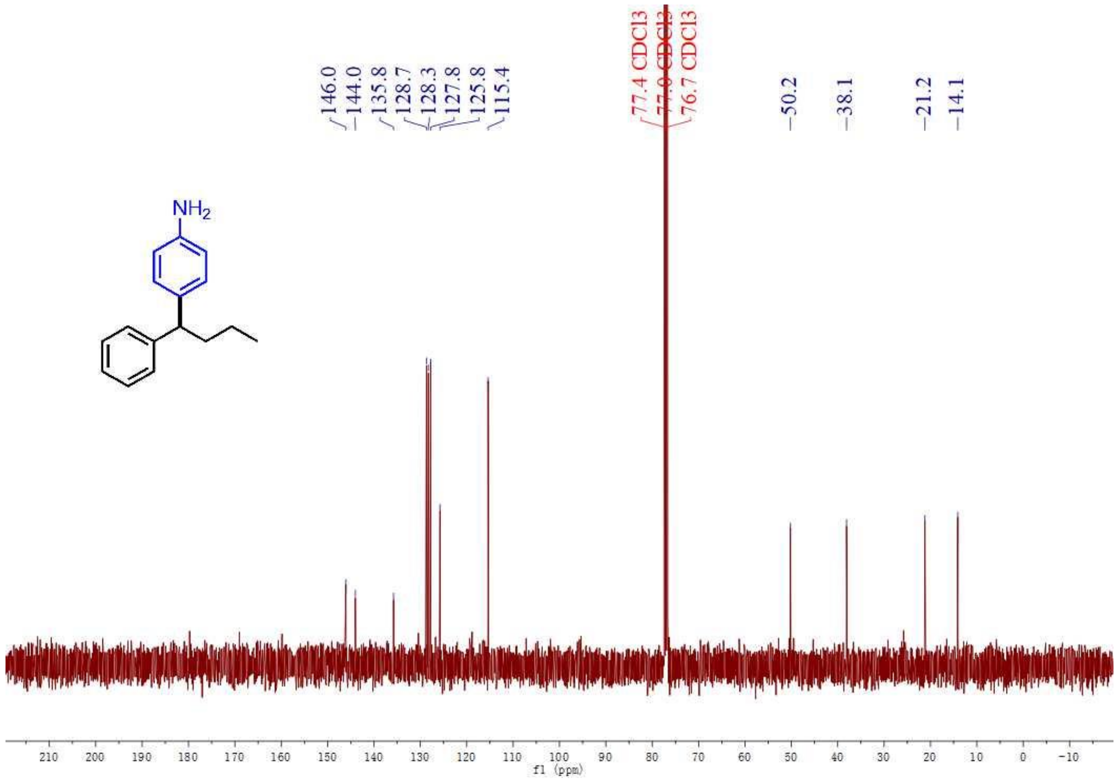

^{1}H NMR spectrum of **7** ($CDCl_3$)

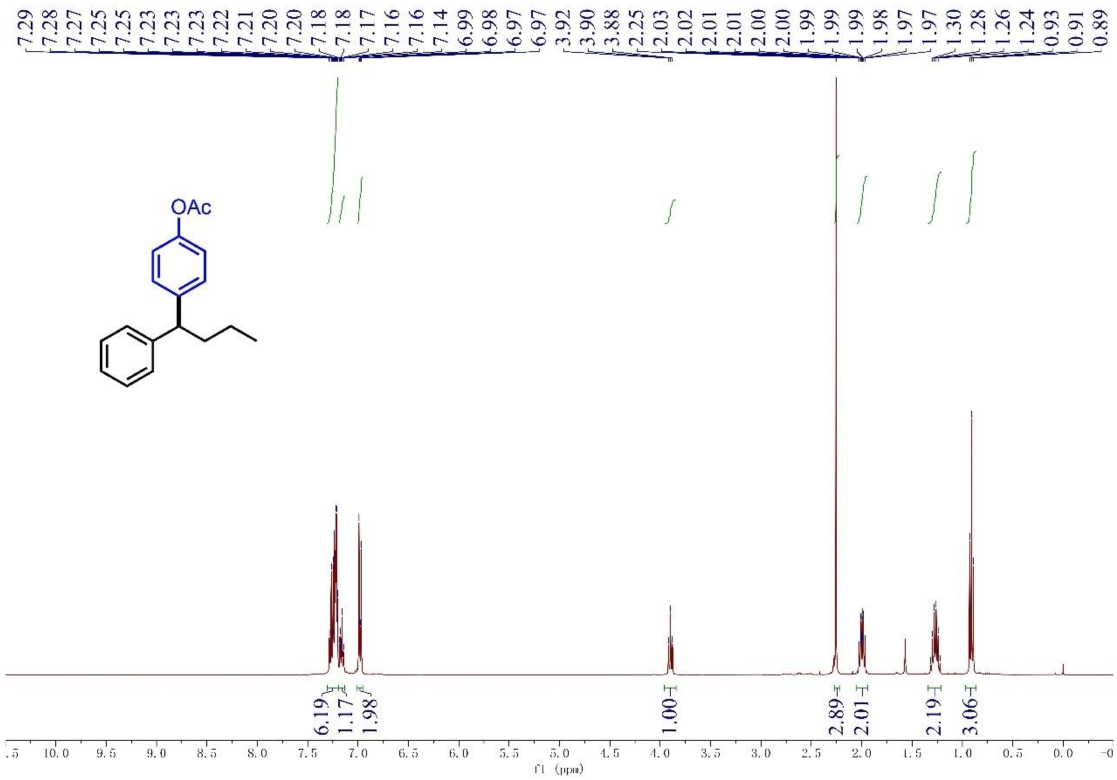

^{13}C NMR spectrum of **7** ($CDCl_3$)

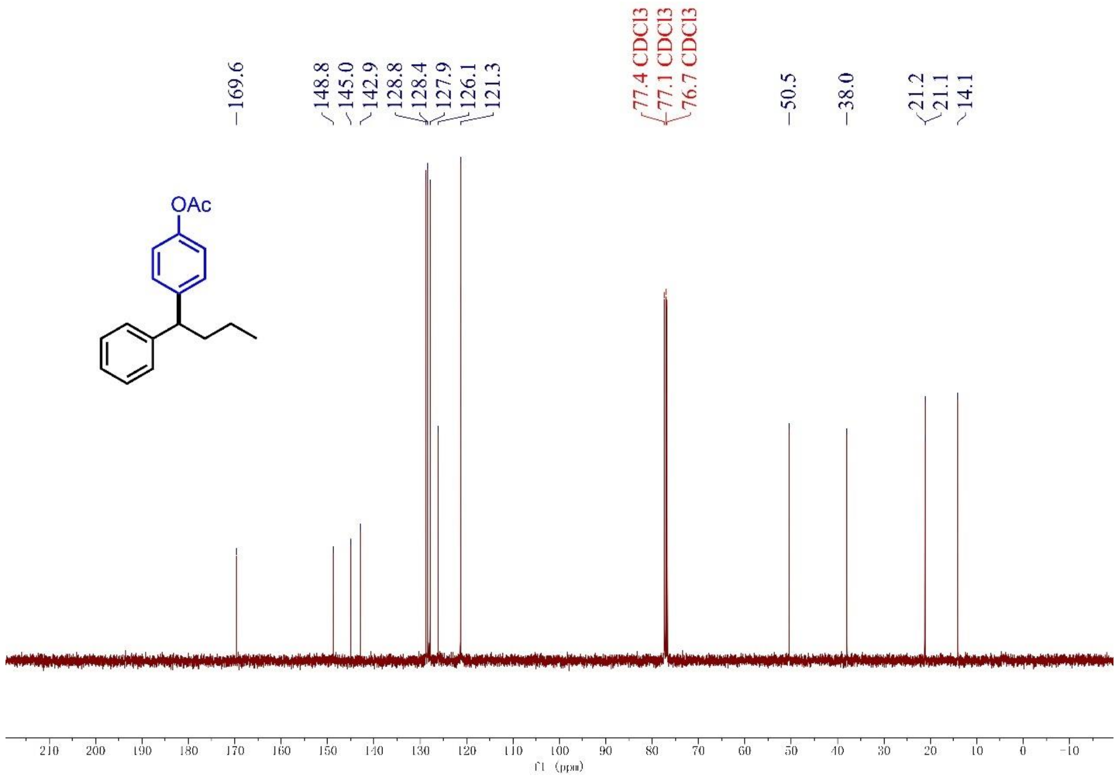

^{1}H NMR spectrum of **8** ($CDCl_3$)

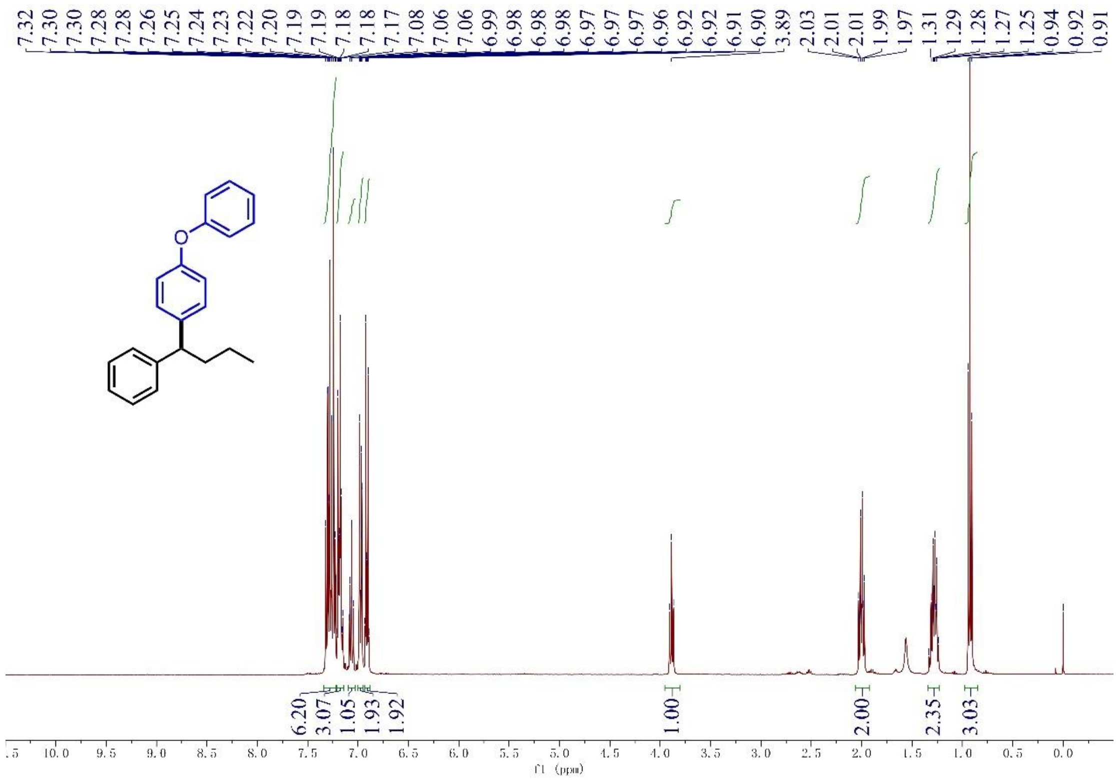

^{13}C NMR spectrum of **8** ($CDCl_3$)

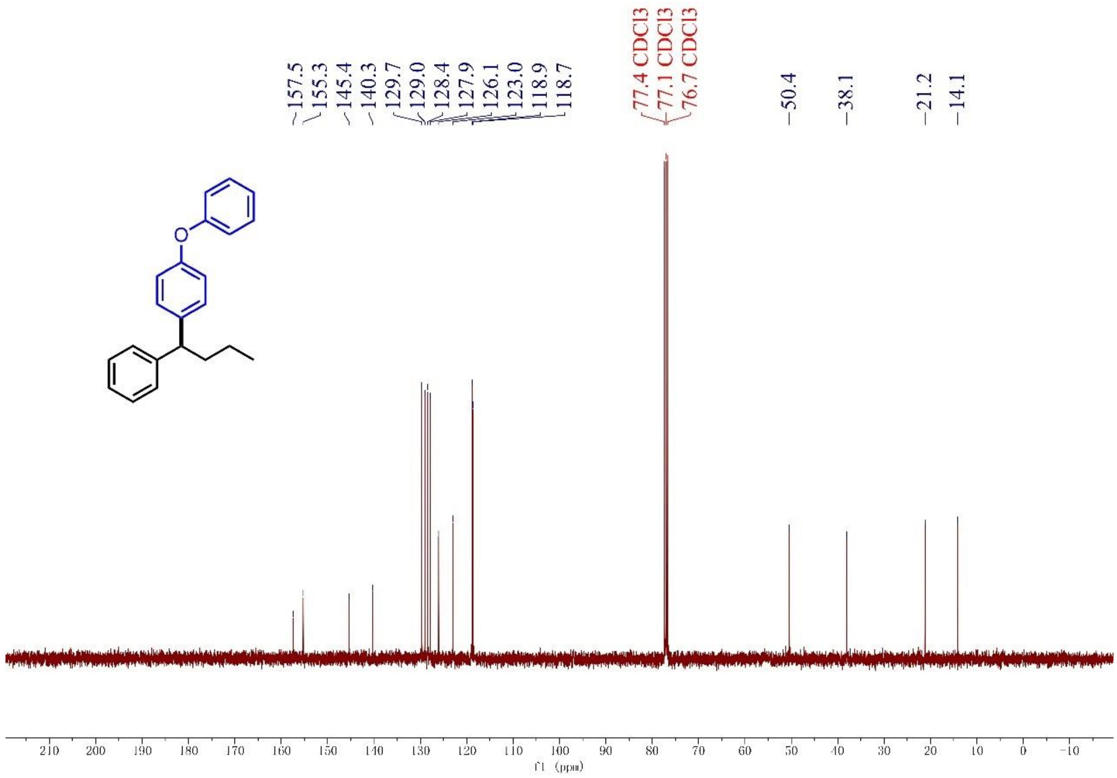

^{1}H NMR spectrum of **9** ($CDCl_3$)

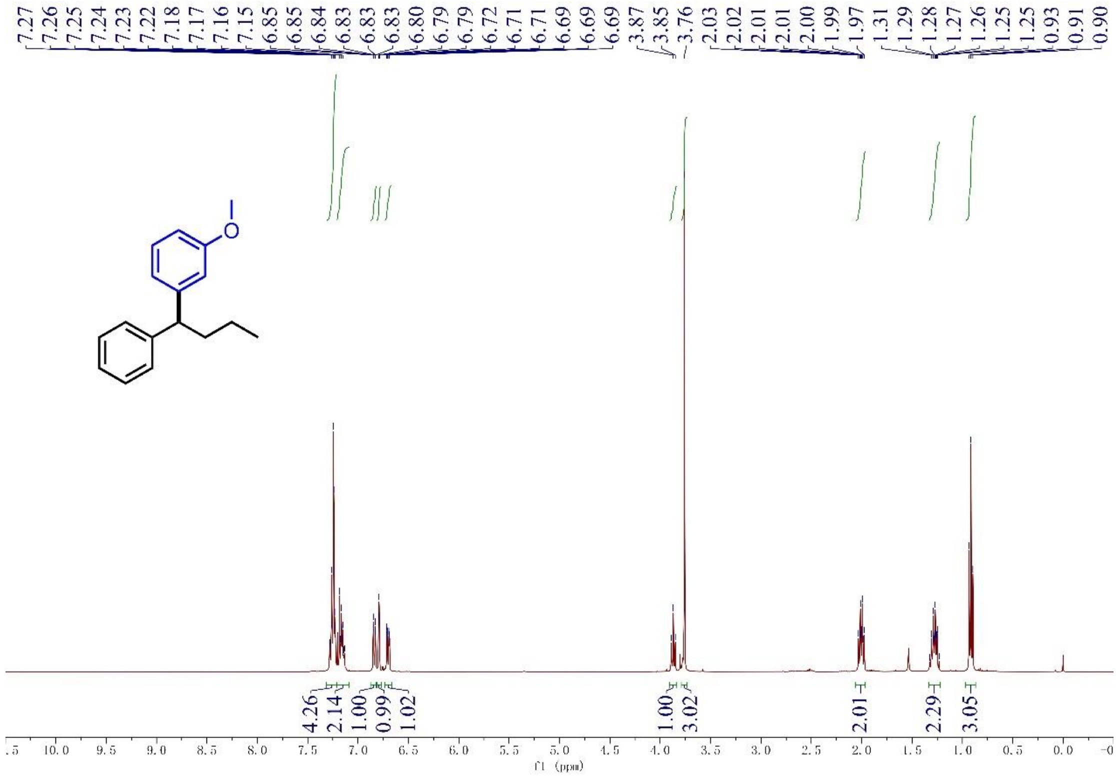

^{13}C NMR spectrum of **9** ($CDCl_3$)

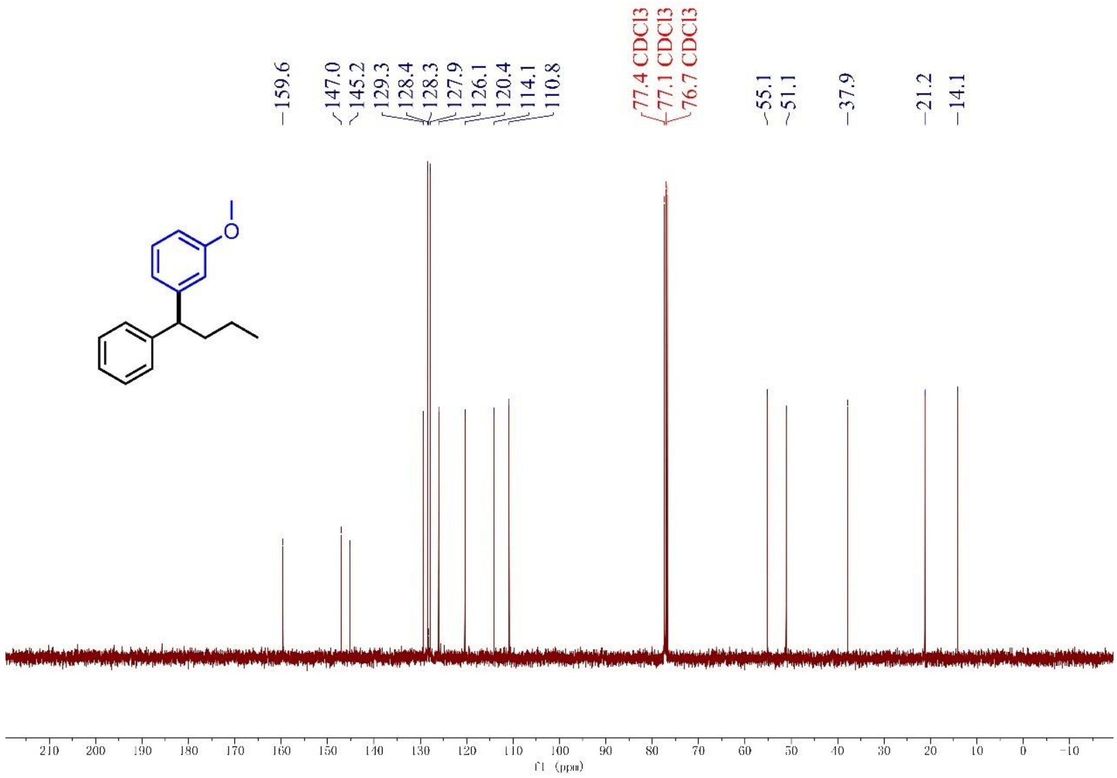

^{1}H NMR spectrum of **10** ($CDCl_3$)

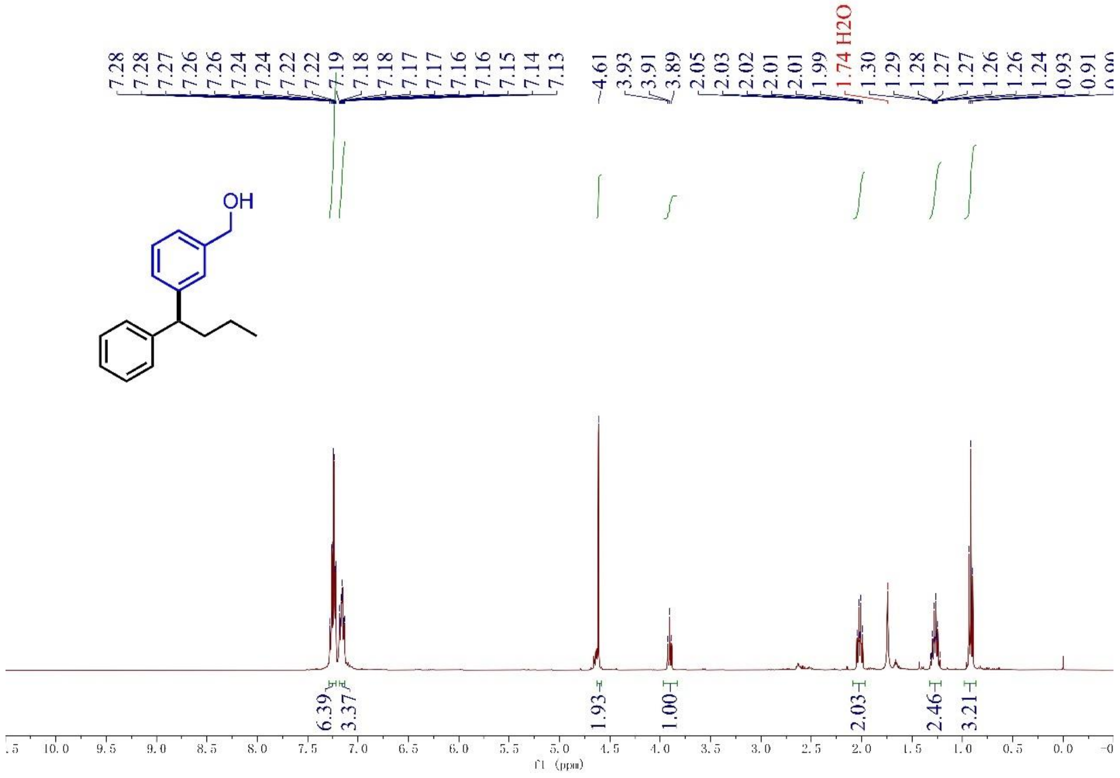

^{13}C NMR spectrum of **10** ($CDCl_3$)

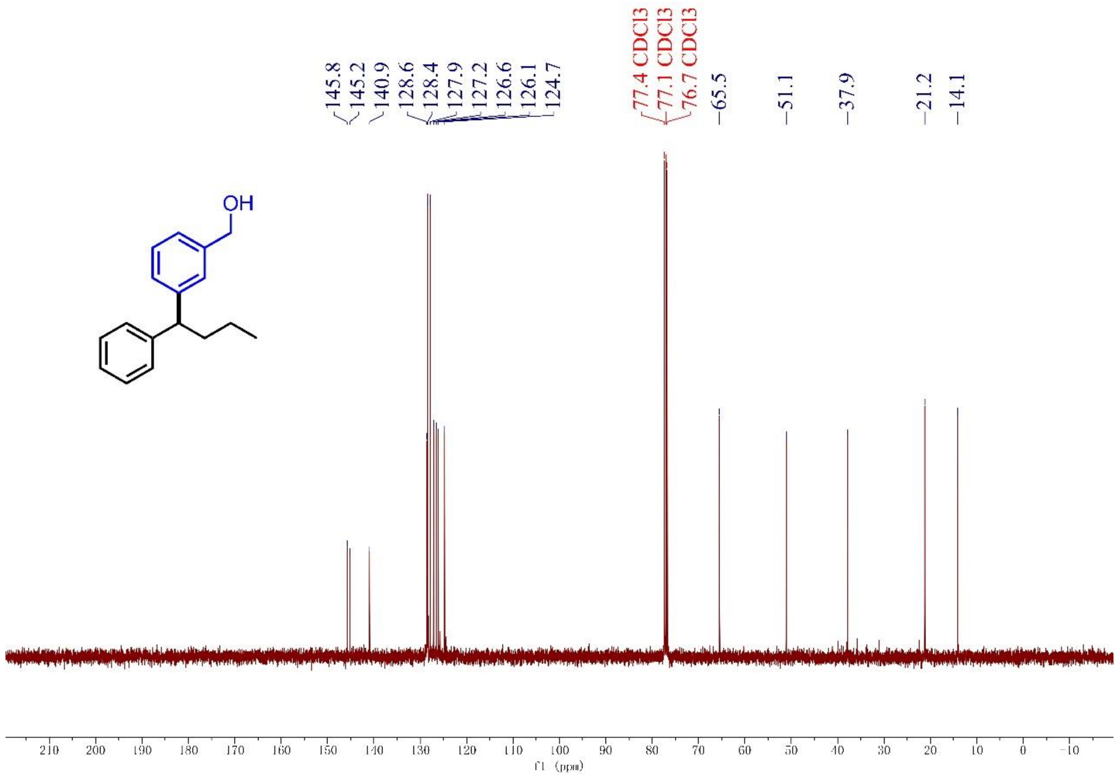

^{1}H NMR spectrum of **11** ($CDCl_3$)

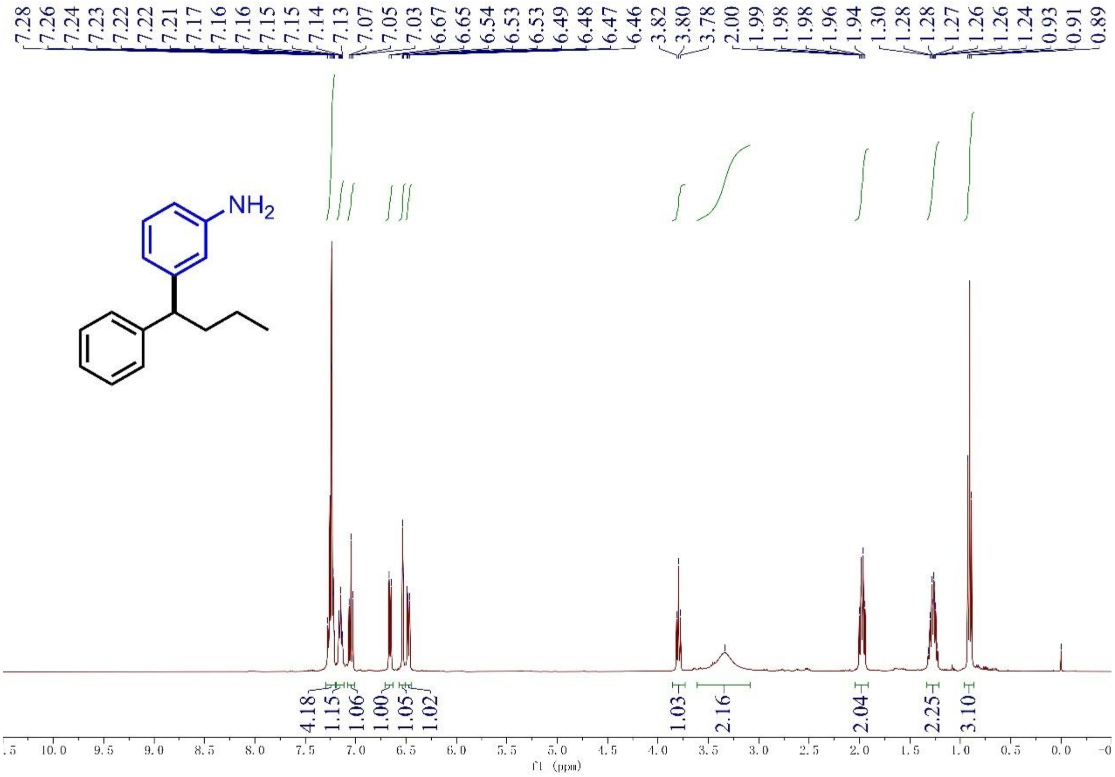

^{13}C NMR spectrum of **11** ($CDCl_3$)

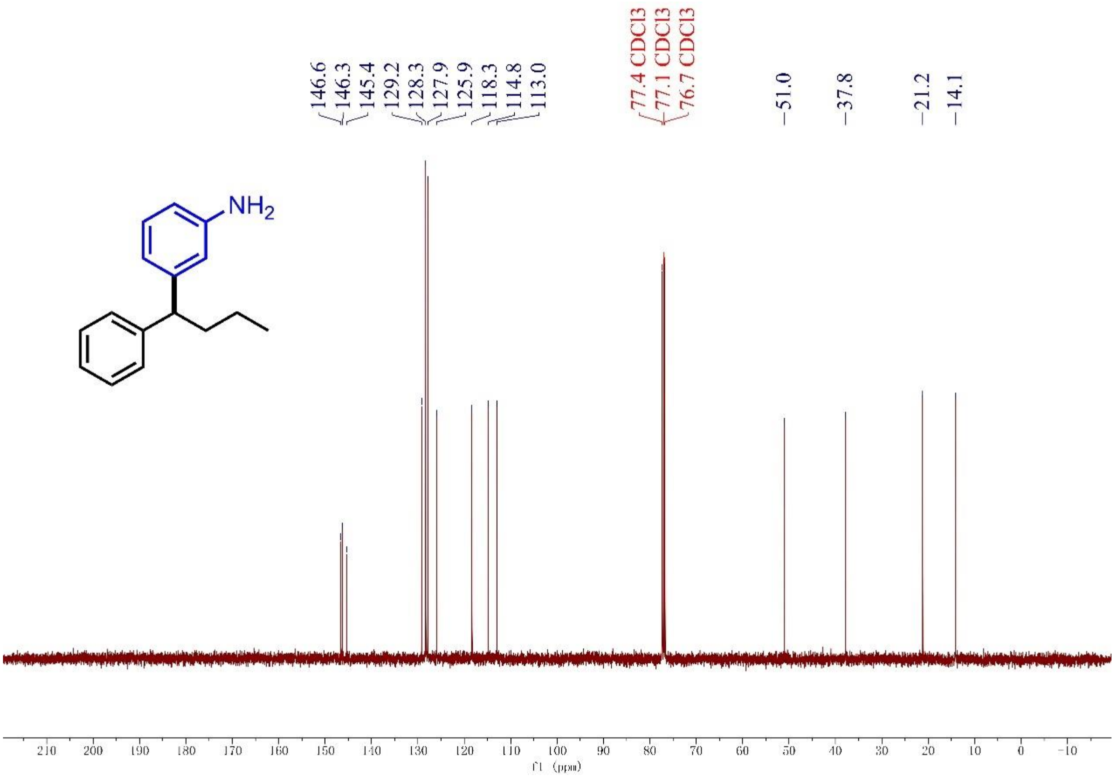

^{1}H NMR spectrum of **12** ($CDCl_3$)

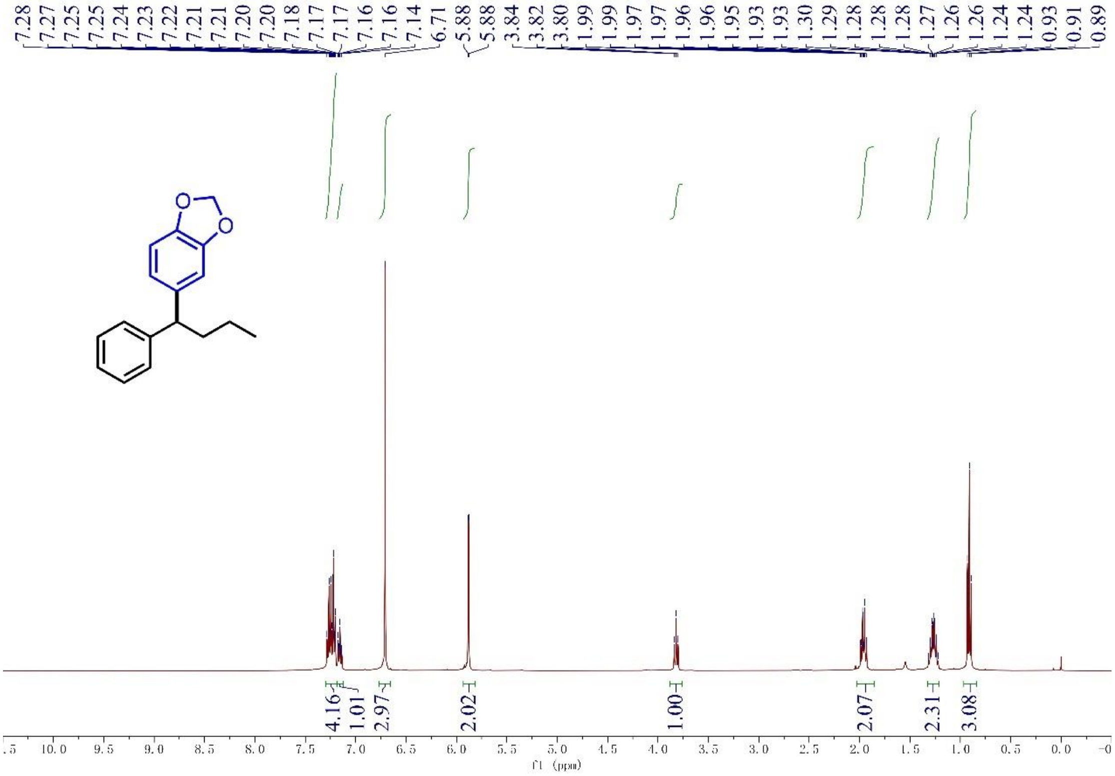

^{13}C NMR spectrum of **12** ($CDCl_3$)

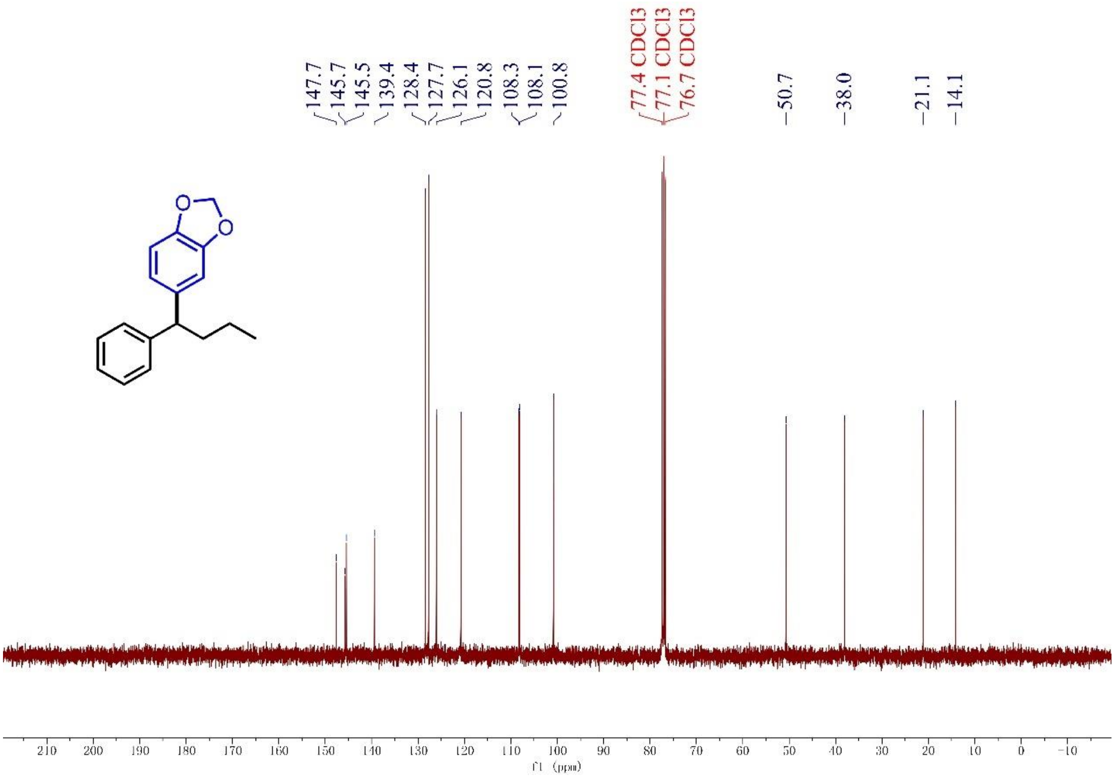

^{1}H NMR spectrum of **13** ($CDCl_3$)

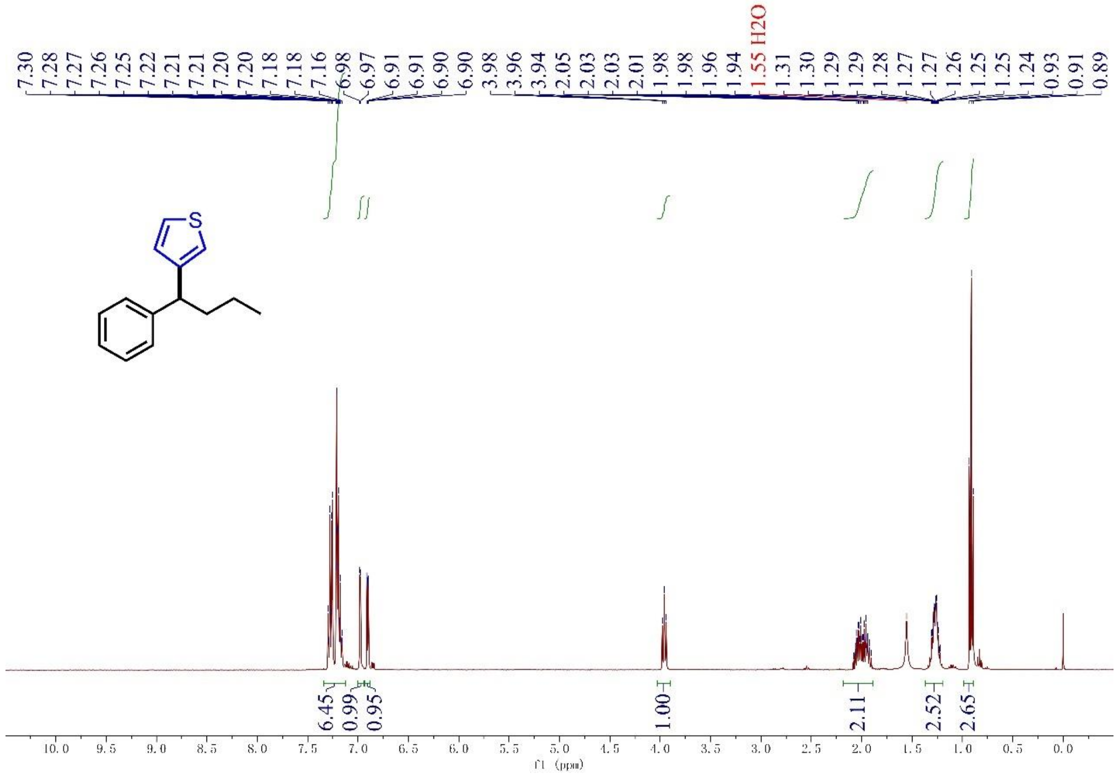

^{13}C NMR spectrum of **13** ($CDCl_3$)

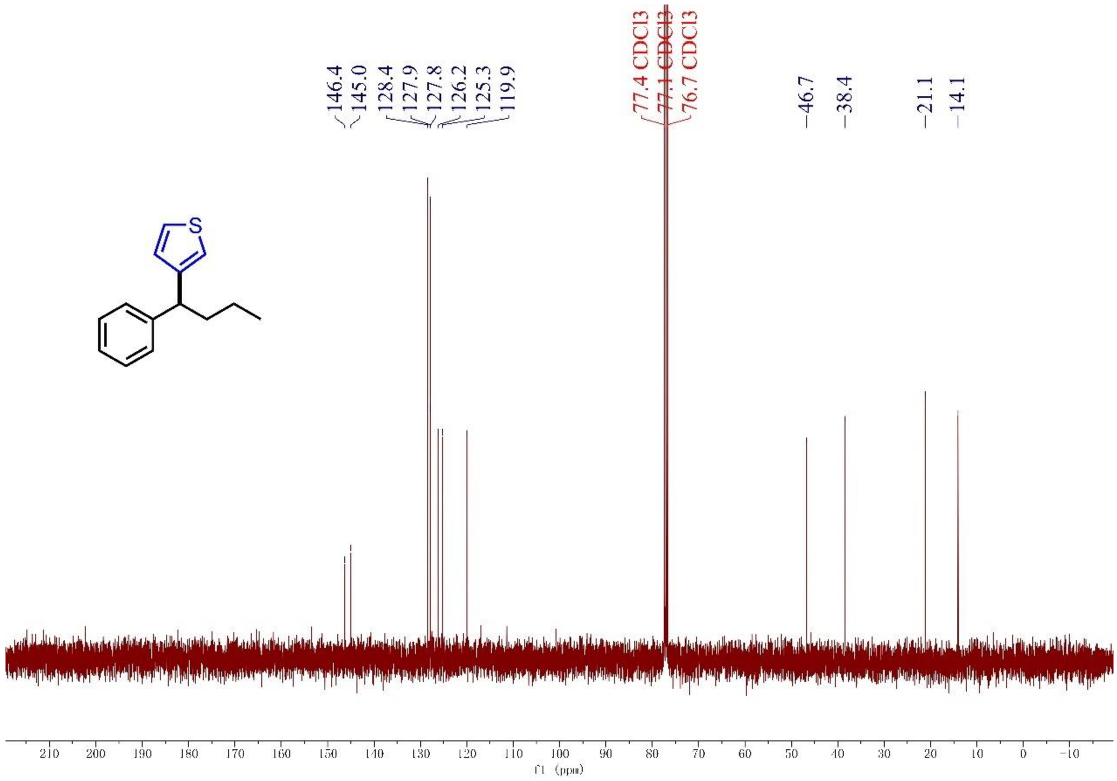

^{1}H NMR spectrum of 14 ($CDCl_3$)

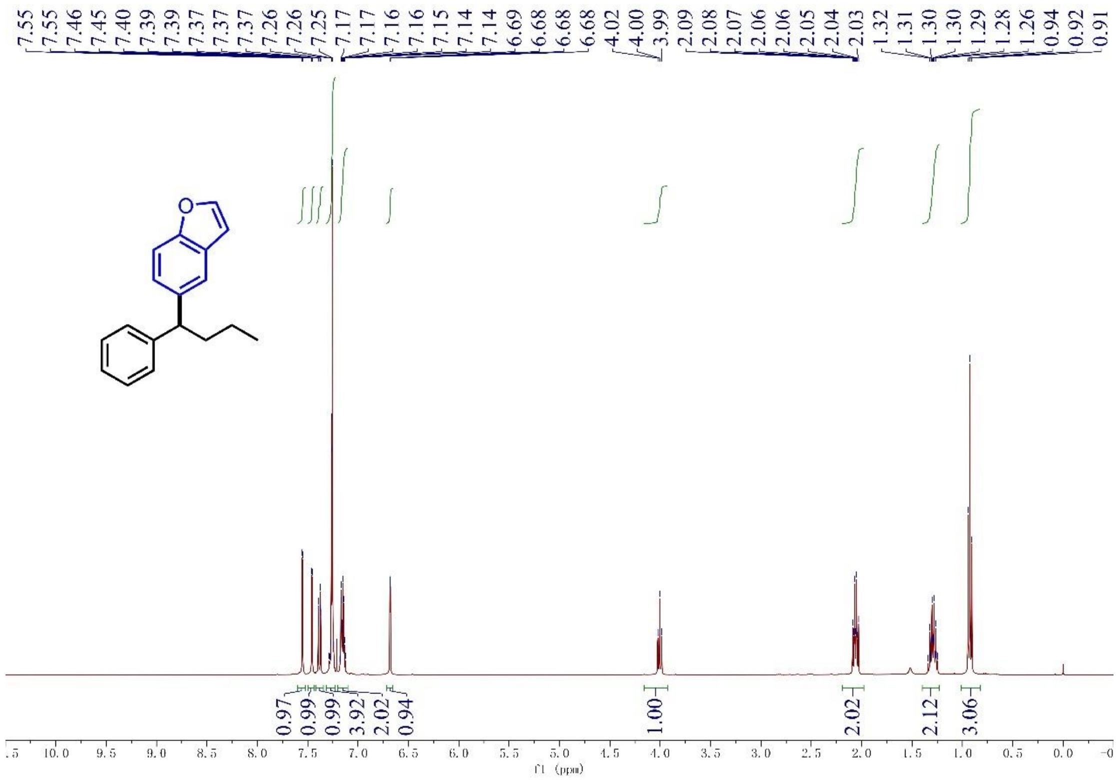

^{13}C NMR spectrum of 14 ($CDCl_3$)

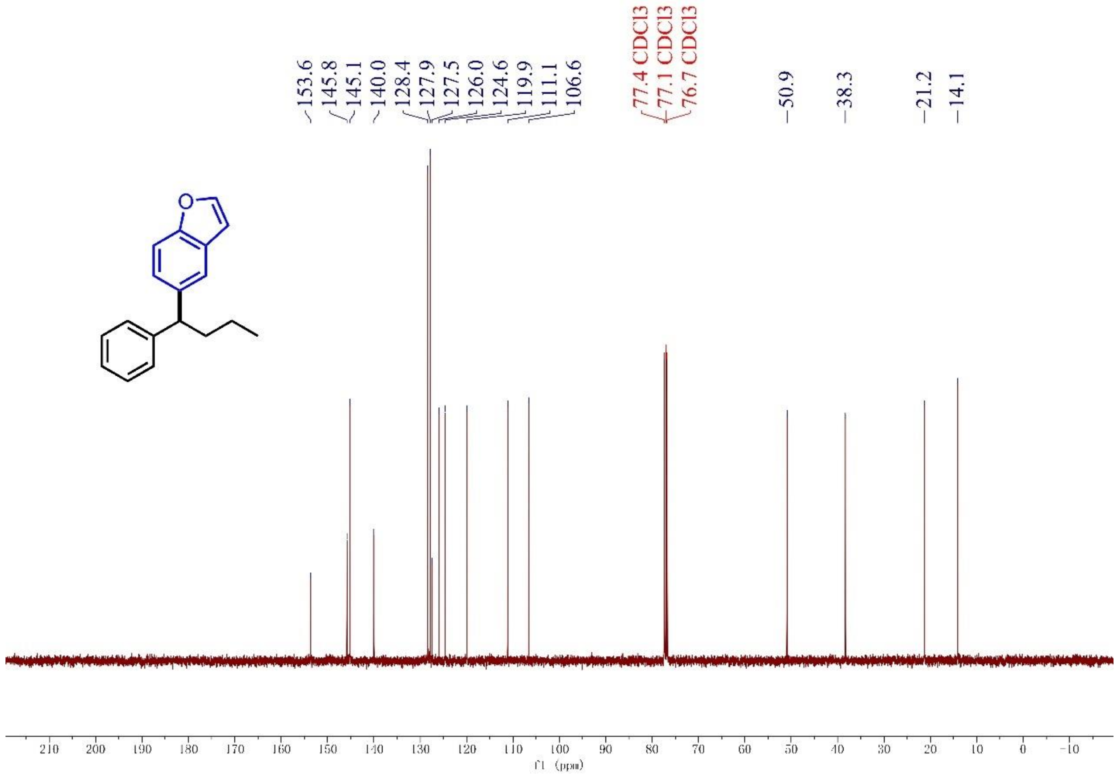

^{1}H NMR spectrum of **15** ($CDCl_3$)

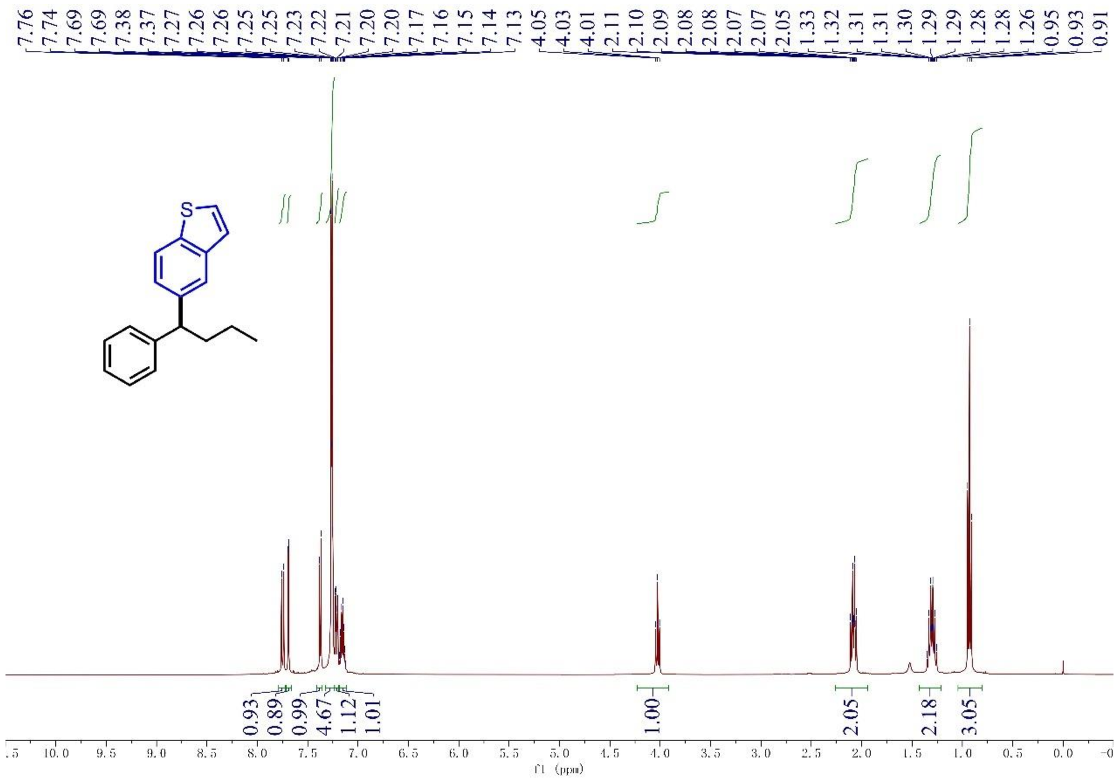

^{13}C NMR spectrum of **15** ($CDCl_3$)

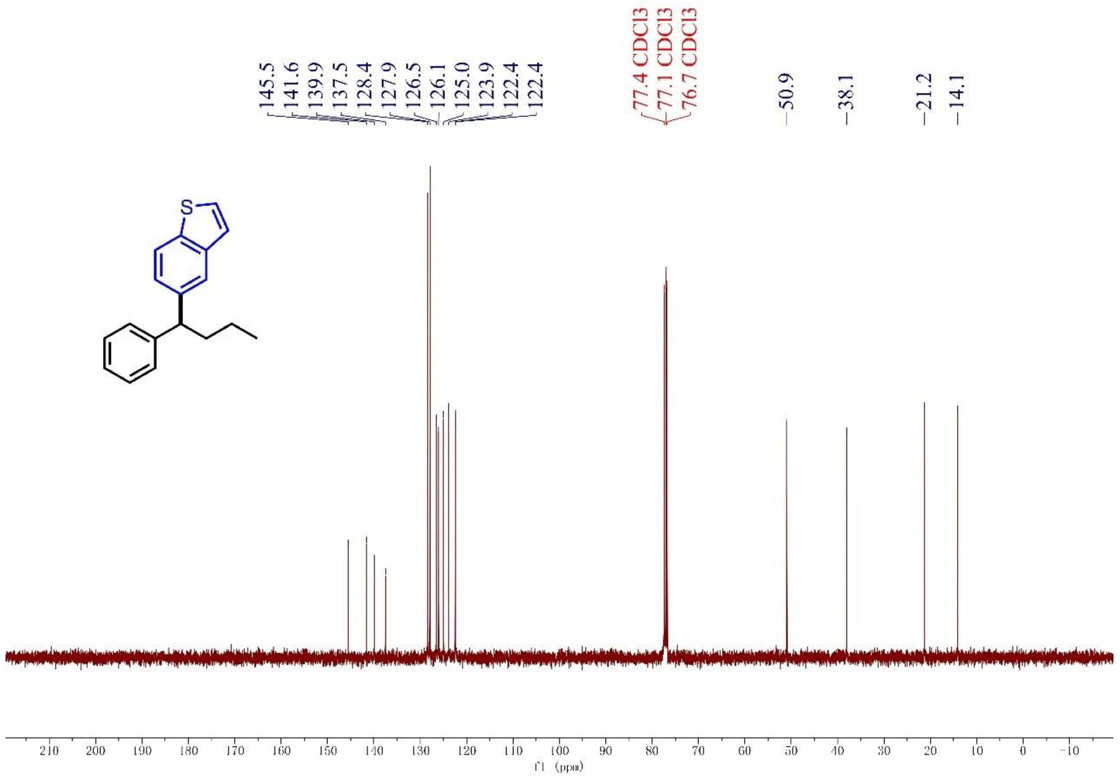

^{1}H NMR spectrum of **16** ($CDCl_3$)

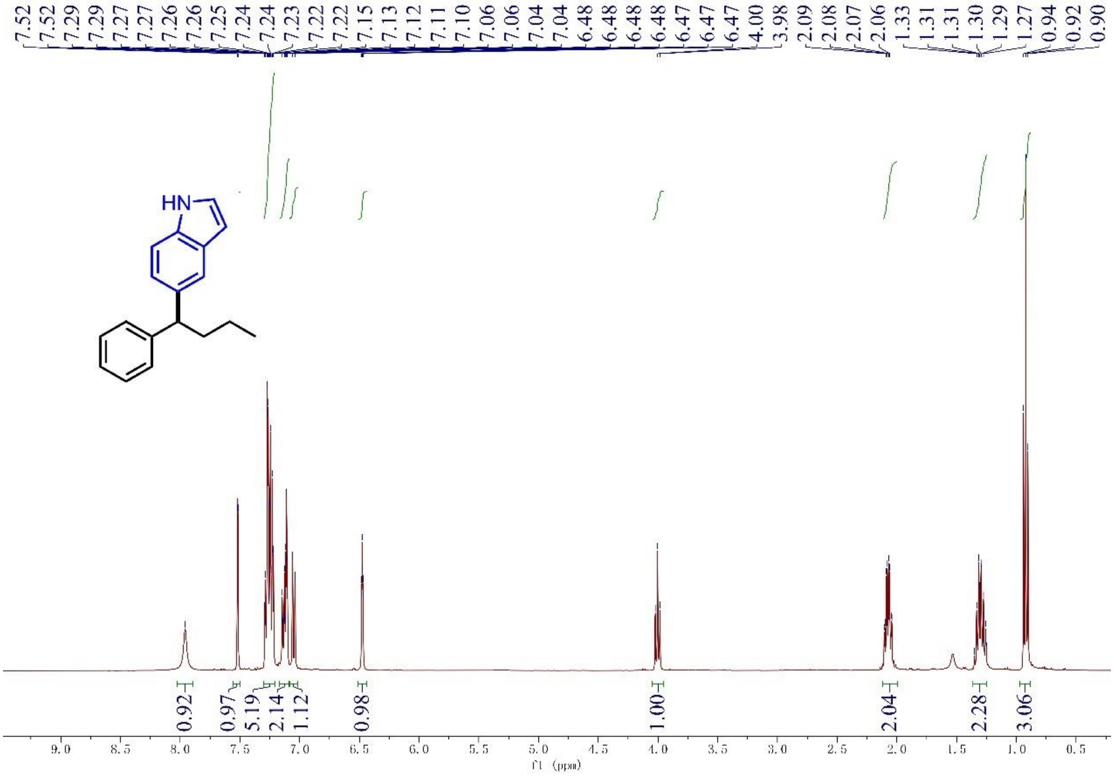

^{13}C NMR spectrum of **16** ($CDCl_3$)

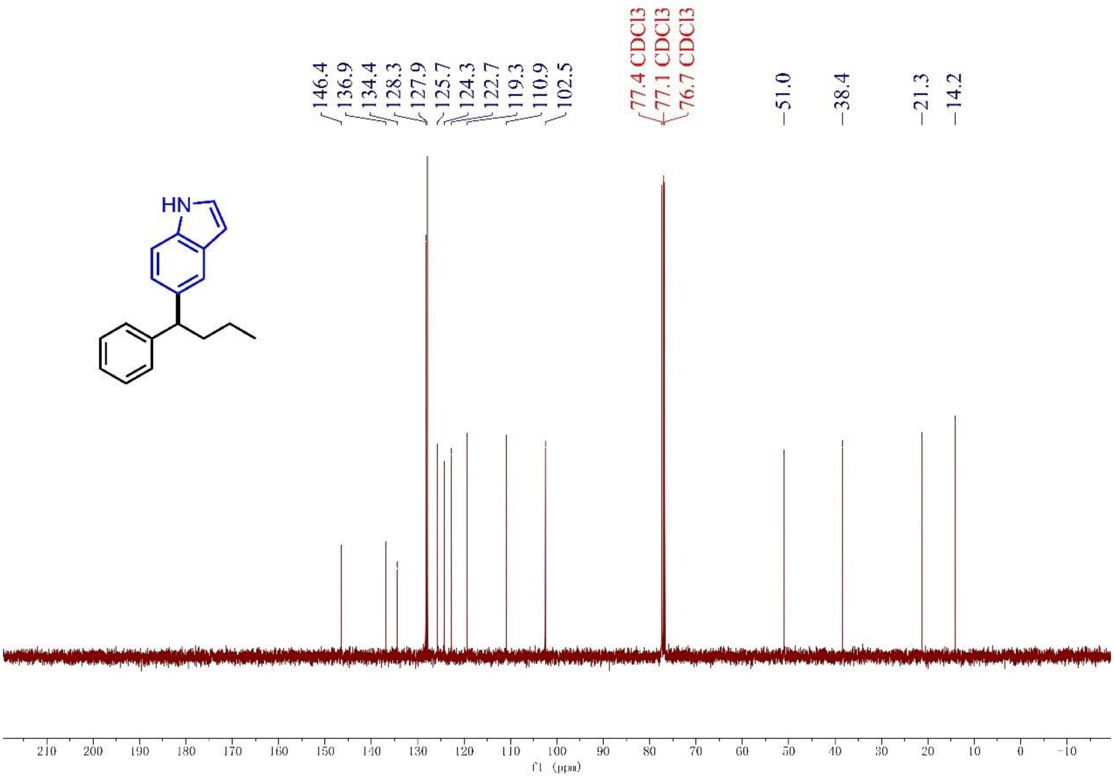

^{1}H NMR spectrum of **17** ($CDCl_3$)

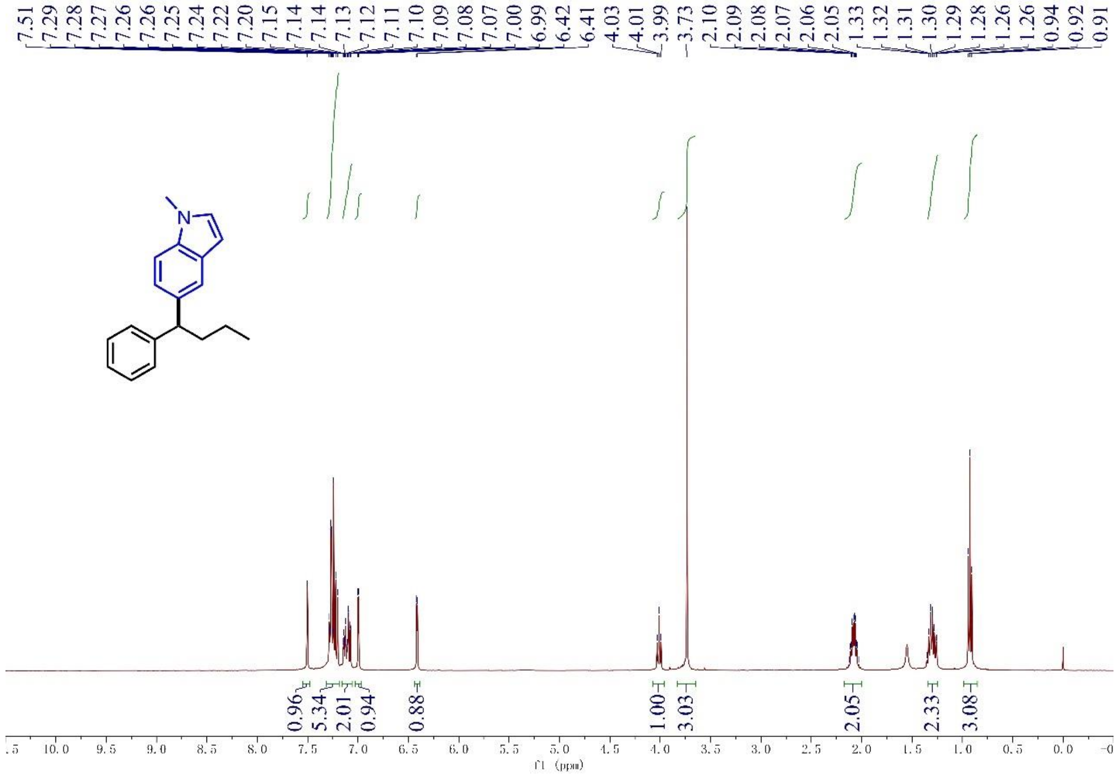

^{13}C NMR spectrum of **17** ($CDCl_3$)

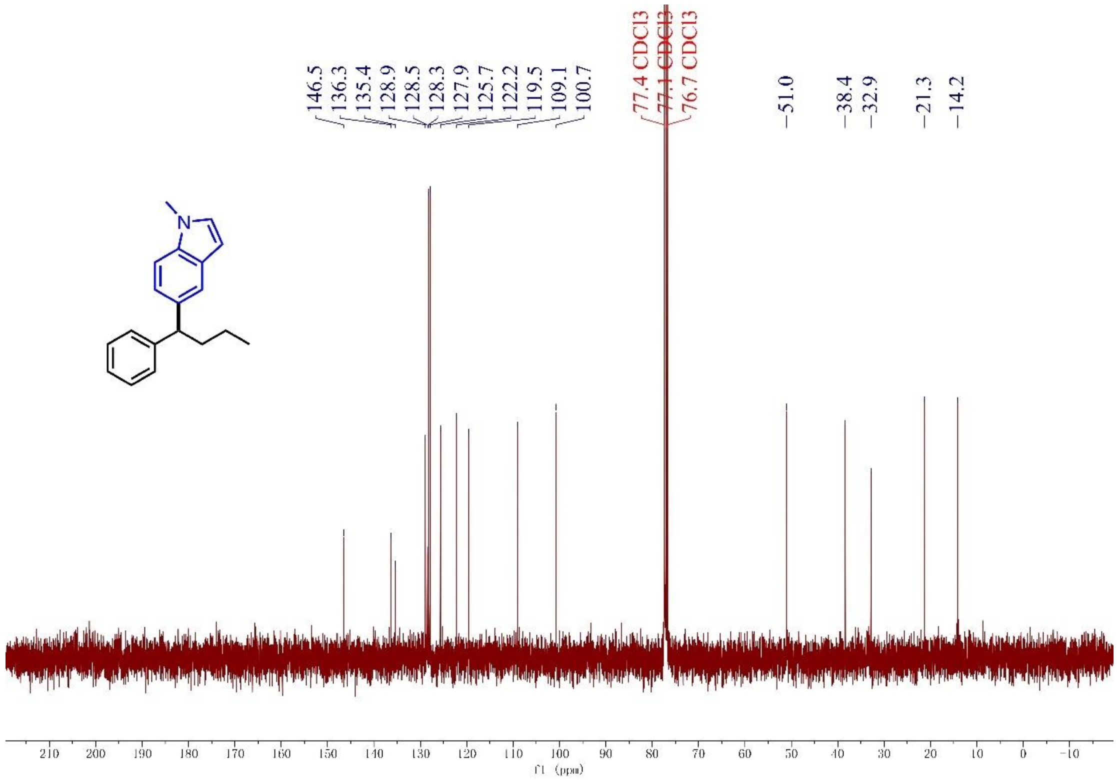

^{1}H NMR spectrum of **18** ($CDCl_3$)

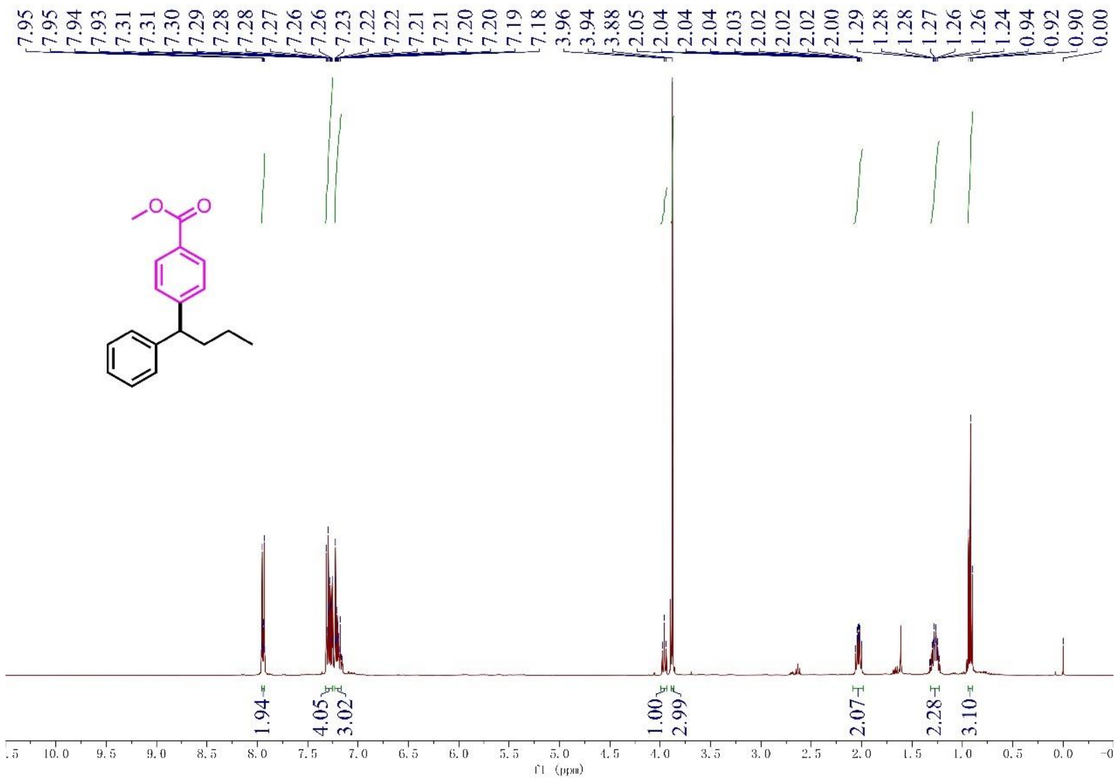

^{13}C NMR spectrum of **18** ($CDCl_3$)

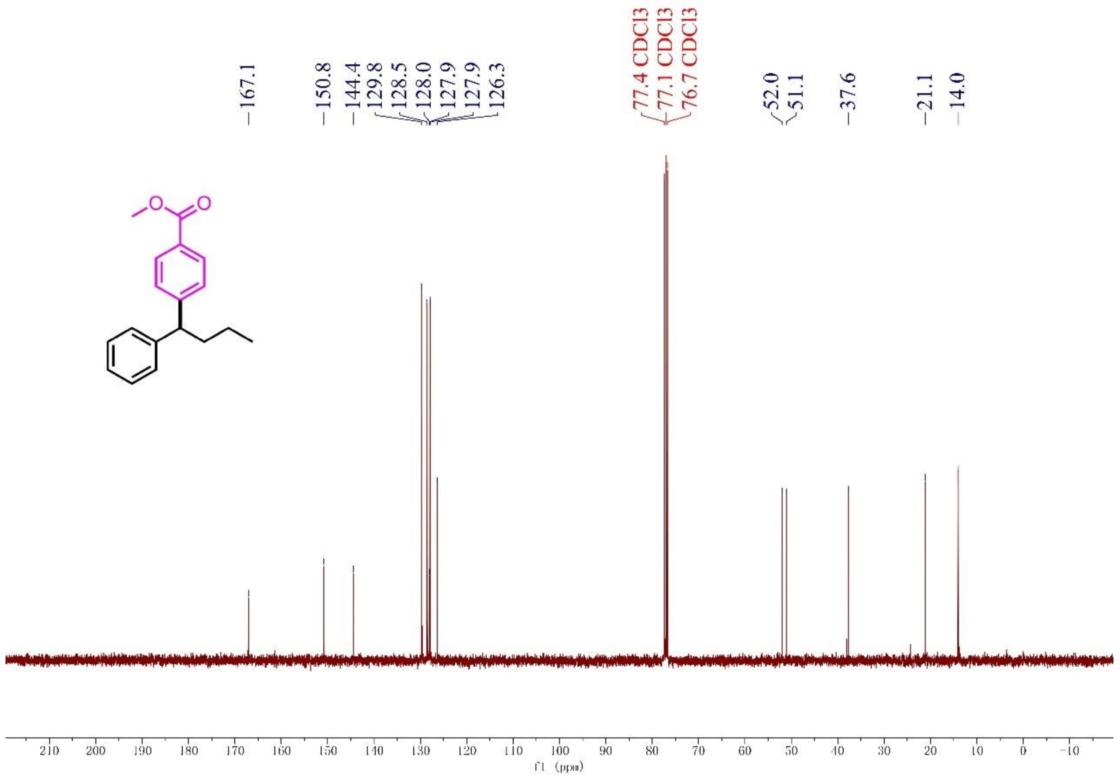

^{1}H NMR spectrum of **19** ($CDCl_3$)

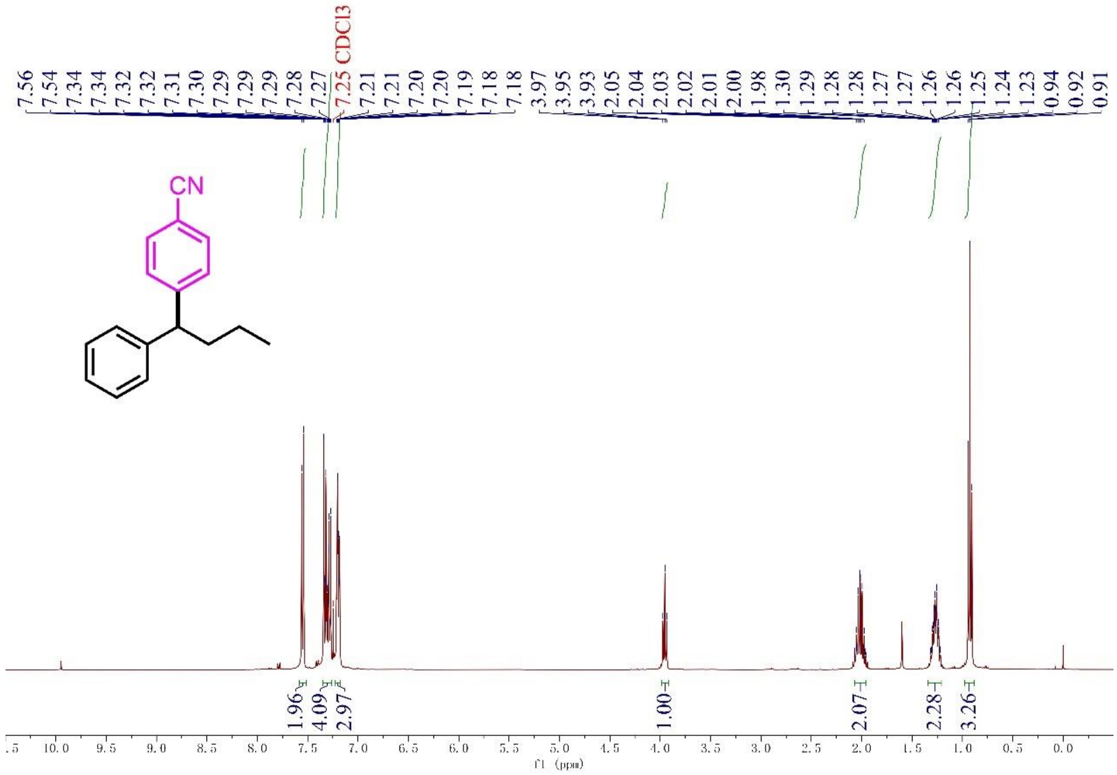

^{13}C NMR spectrum of **19** ($CDCl_3$)

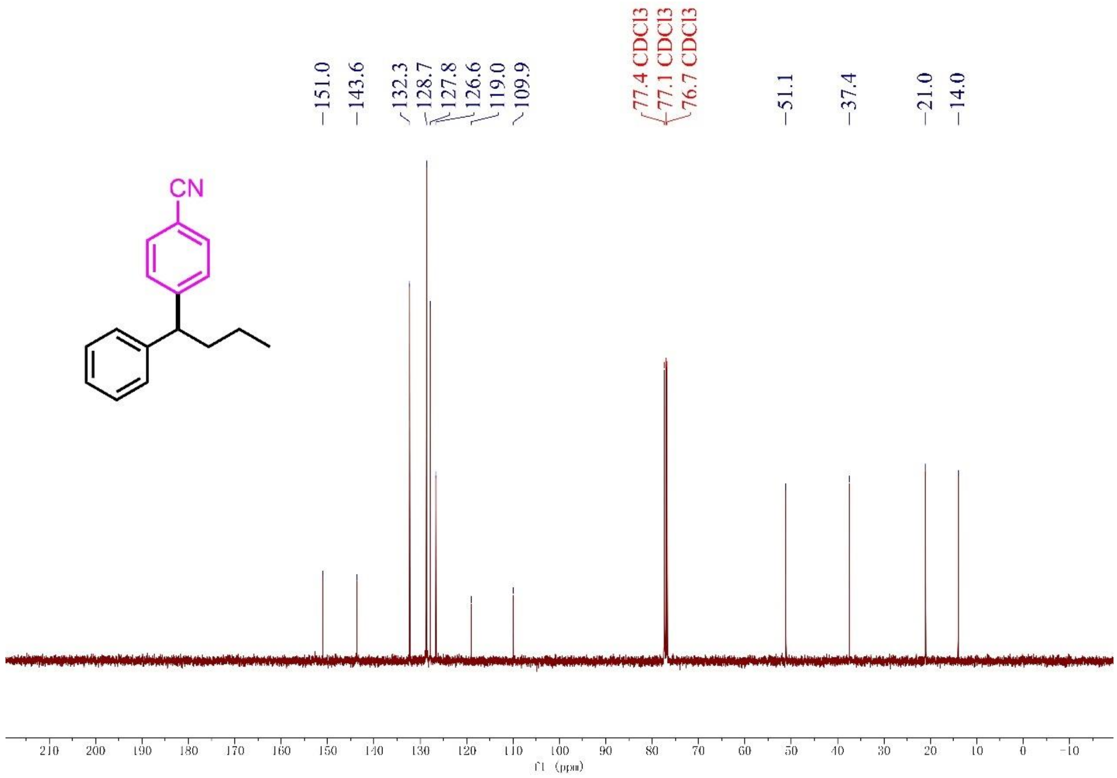

^{1}**H NMR** spectrum of **20** ($CDCl_3$)

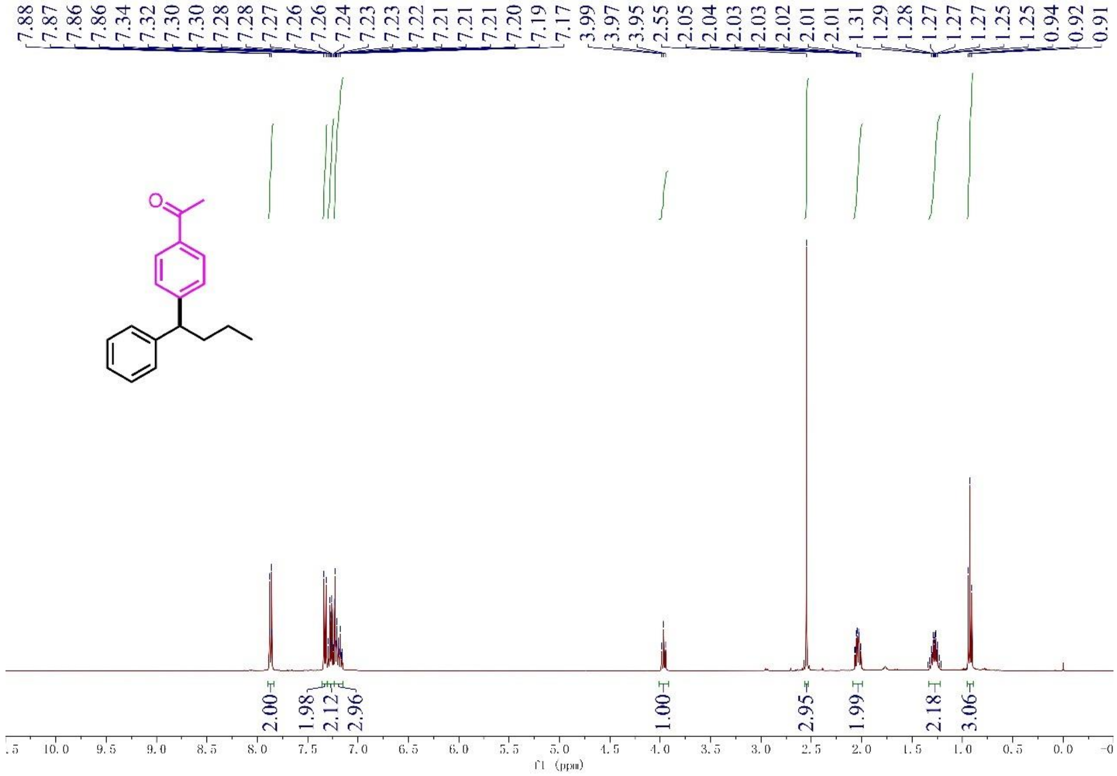

^{13}**C NMR** spectrum of **20** ($CDCl_3$)

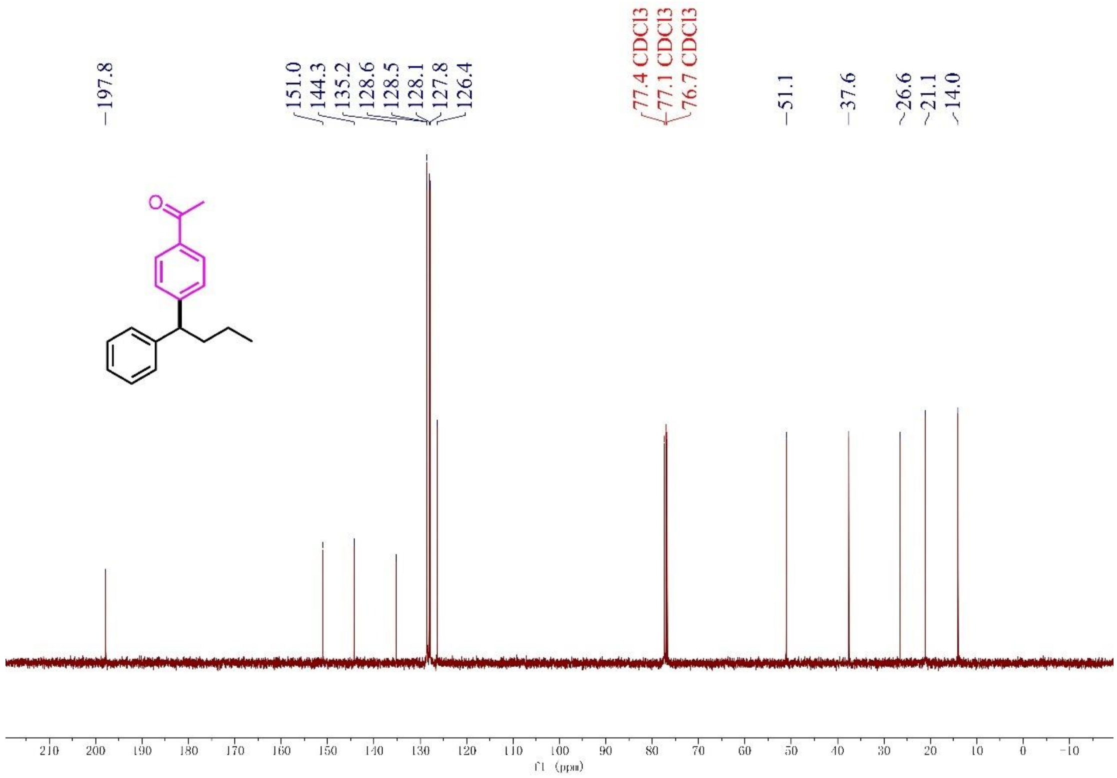

^{1}H NMR spectrum of **21** ($CDCl_3$)

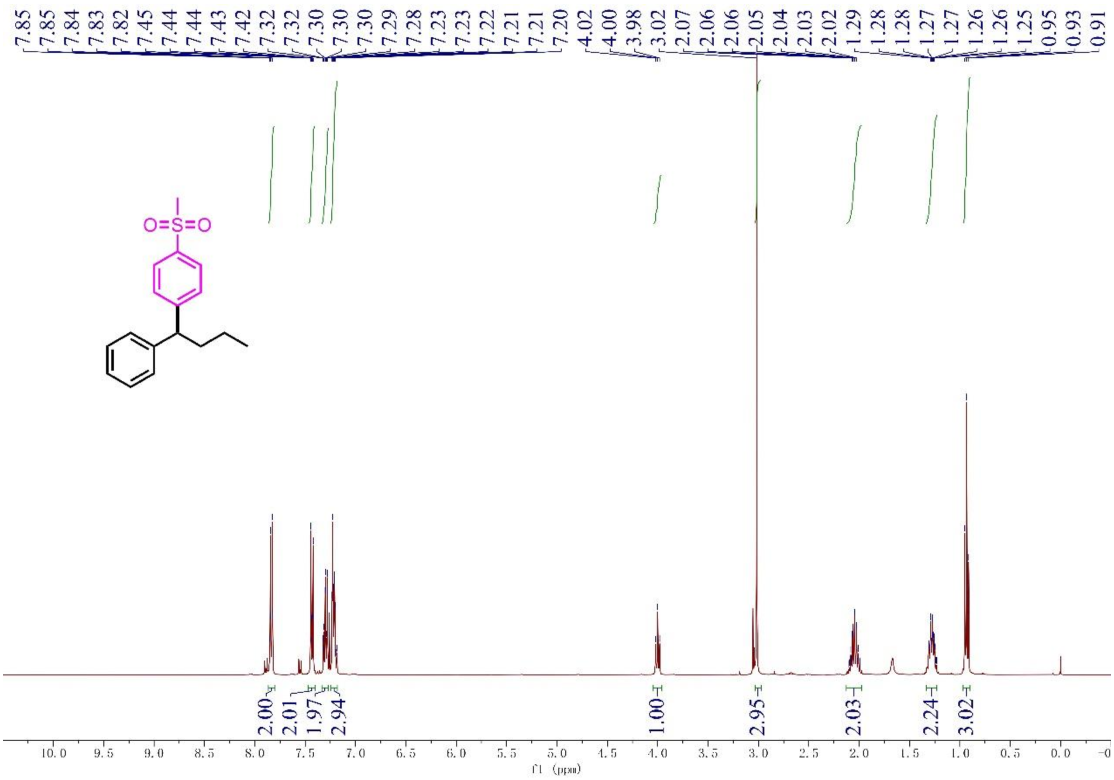

^{13}C NMR spectrum of **21** ($CDCl_3$)

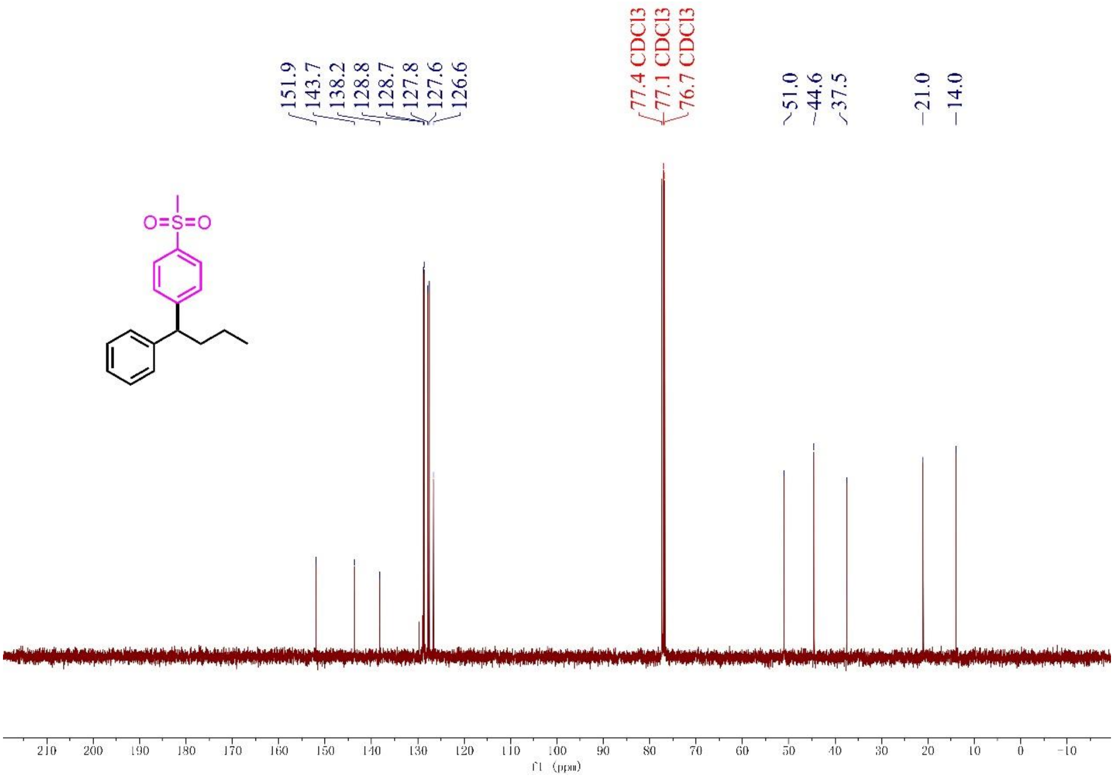

^{1}H NMR spectrum of **22** ($CDCl_3$)

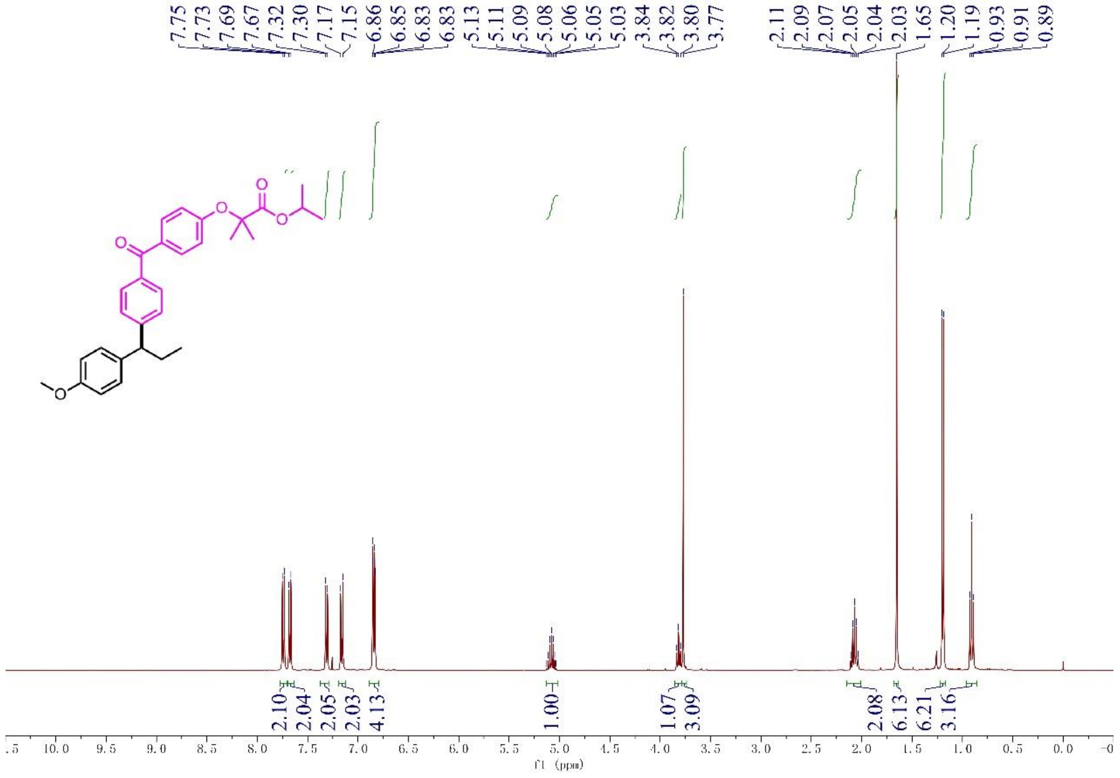

^{13}C NMR spectrum of **22** ($CDCl_3$)

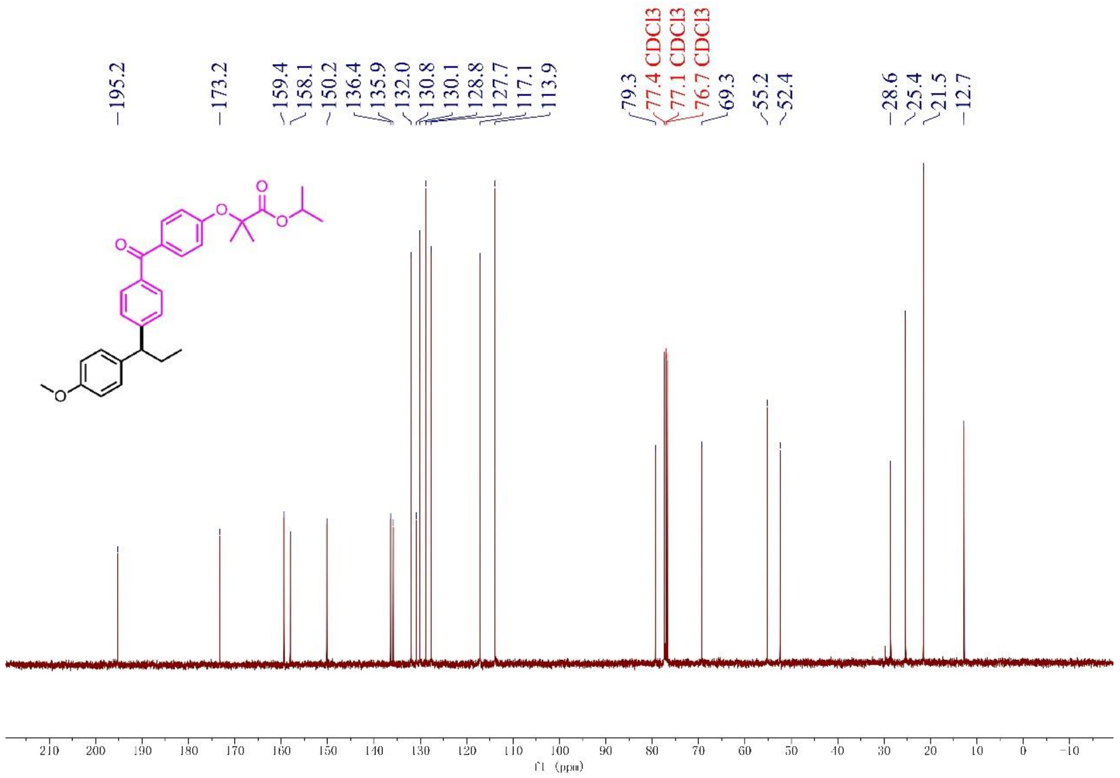

^{1}H NMR spectrum of **23** ($CDCl_3$)

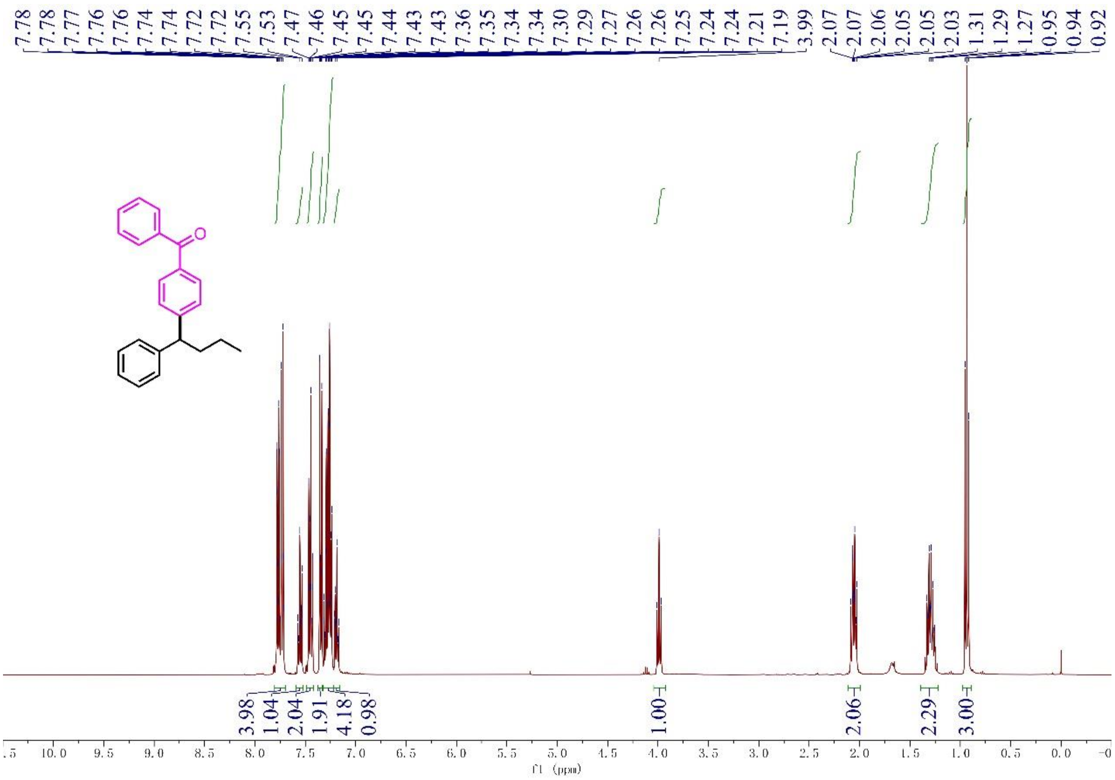

^{13}C NMR spectrum of **23** ($CDCl_3$)

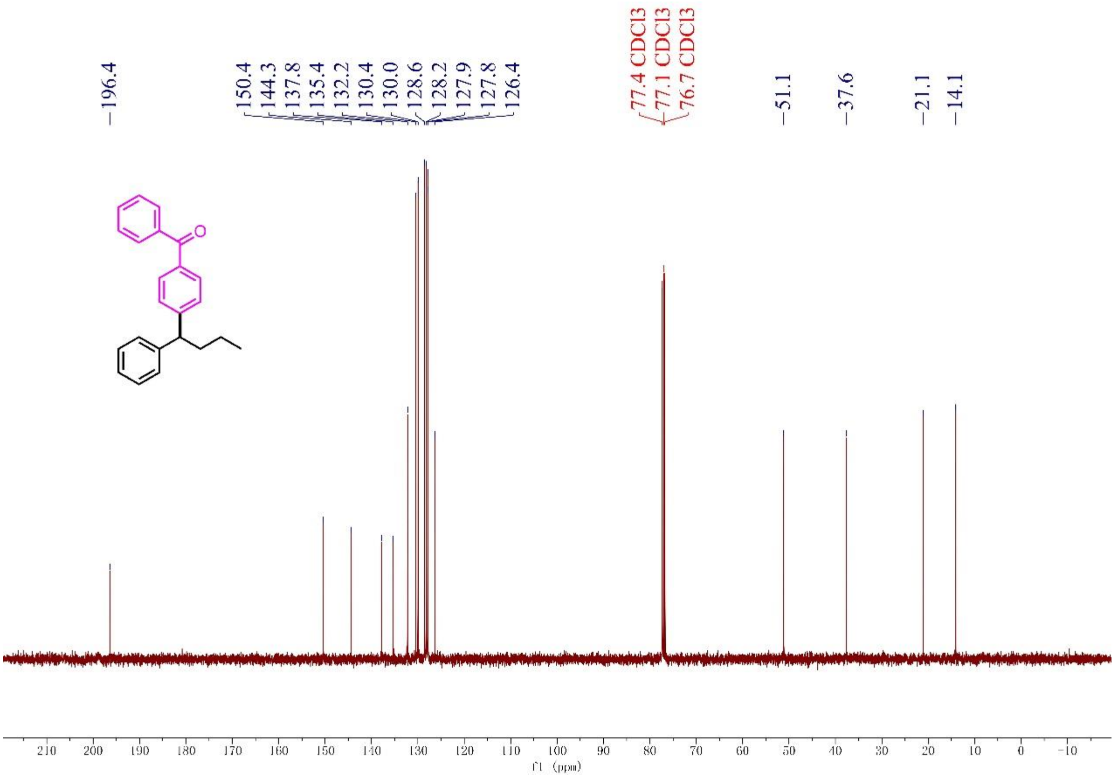

^{1}H NMR spectrum of **24** ($CDCl_3$)

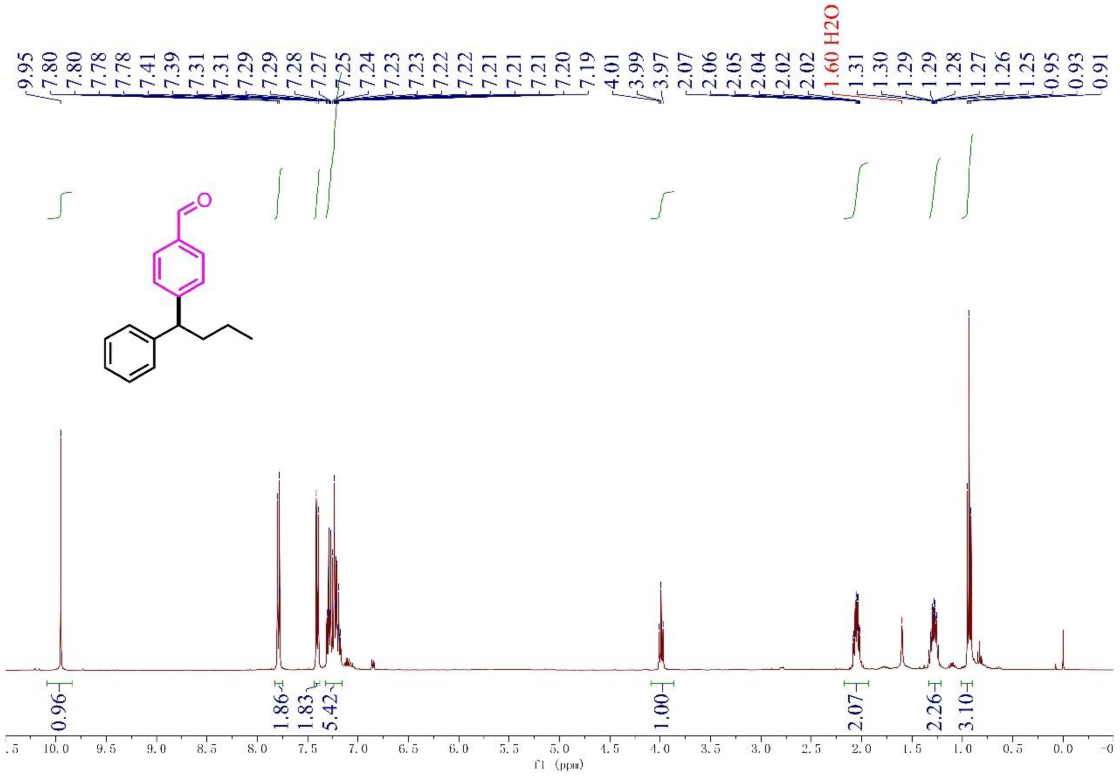

^{13}C NMR spectrum of **24** ($CDCl_3$)

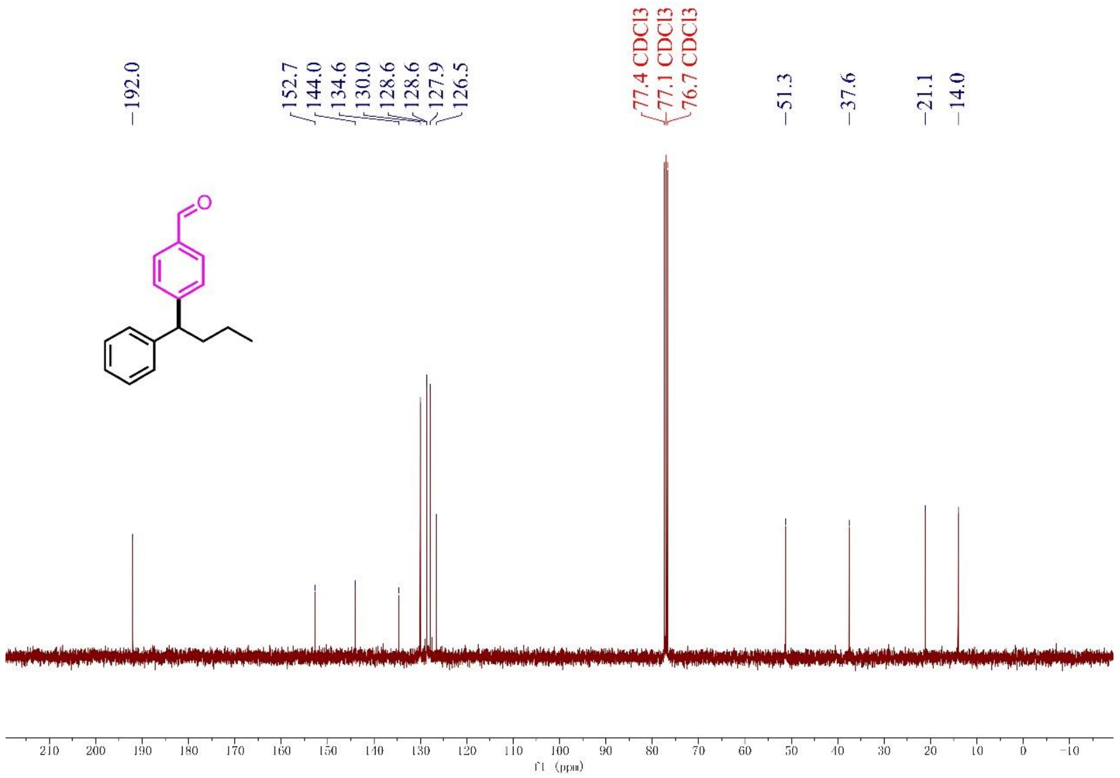

^{1}H NMR spectrum of **25** ($CDCl_3$)

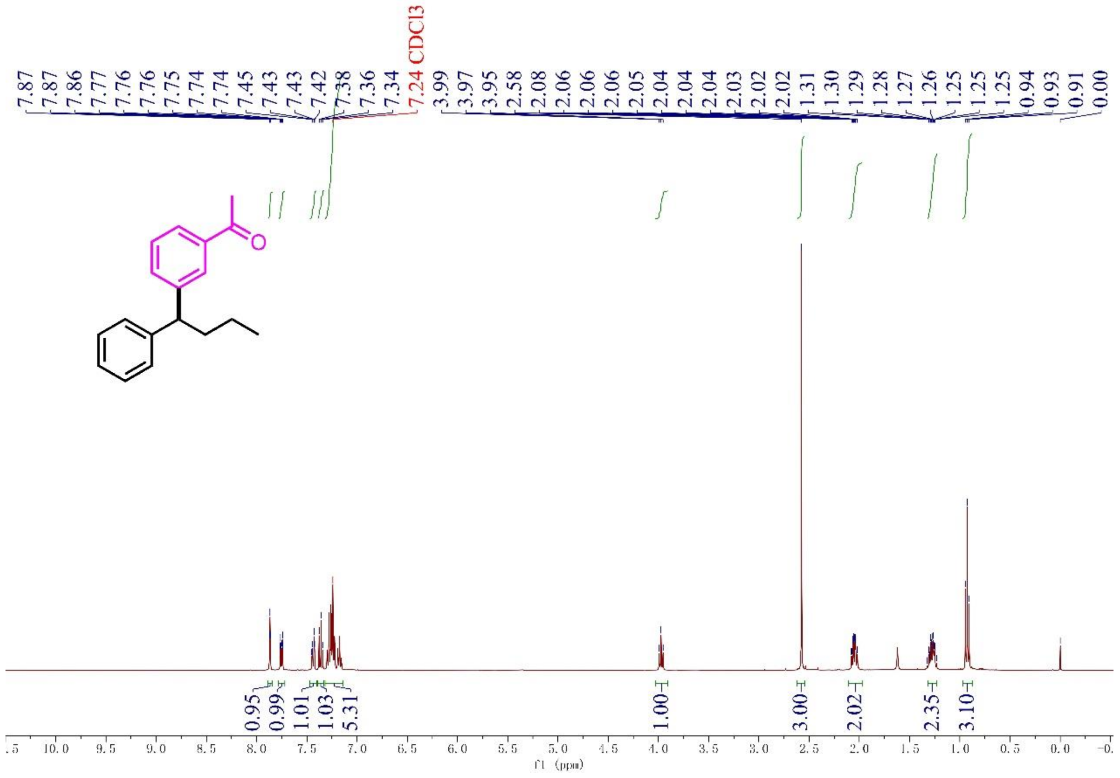

^{13}C NMR spectrum of **25** ($CDCl_3$)

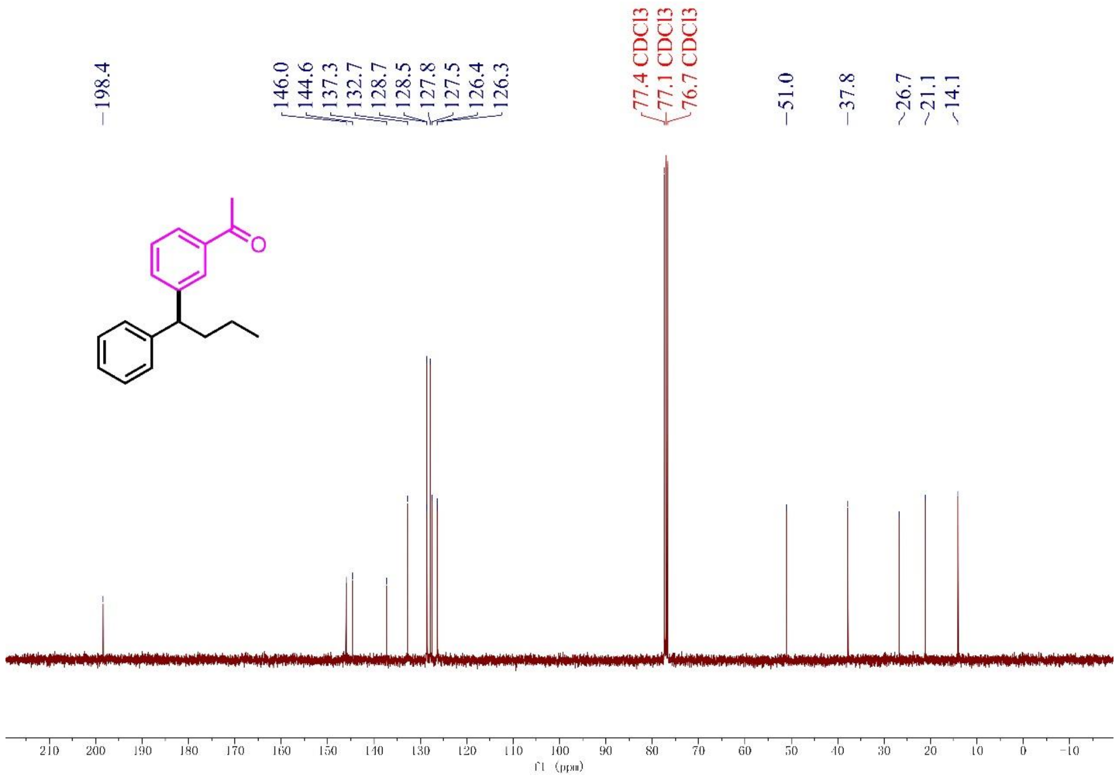

^{1}H NMR spectrum of **26** ($CDCl_3$)

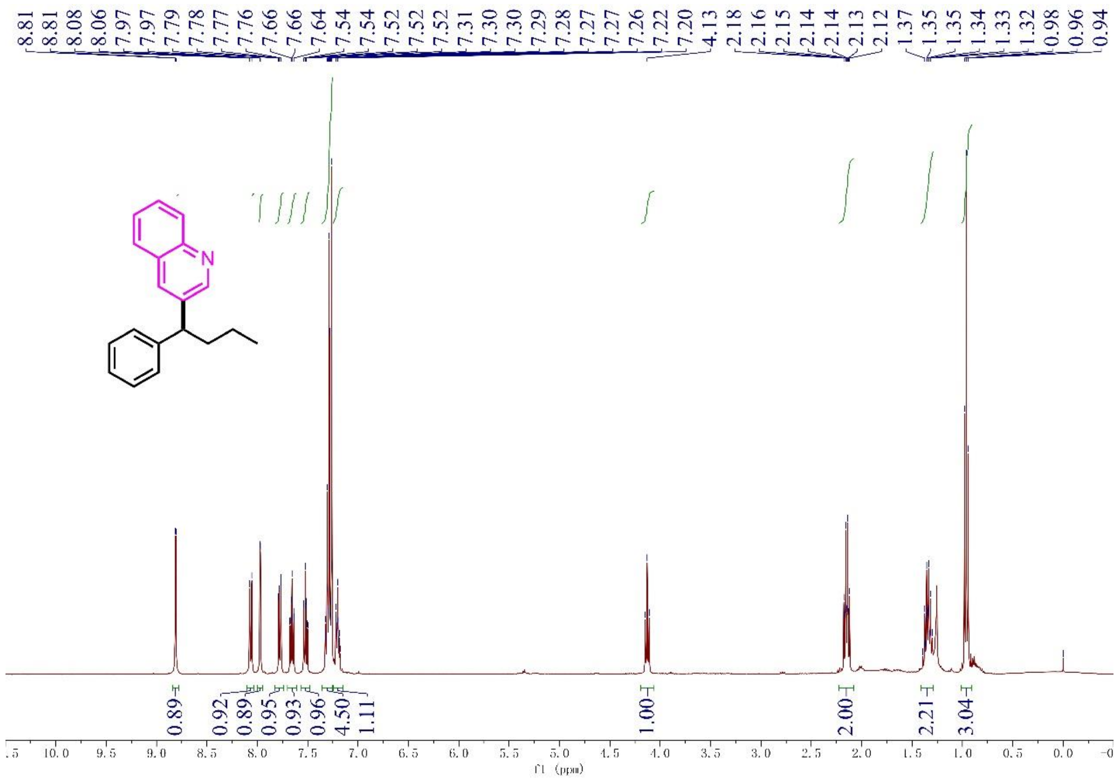

^{13}C NMR spectrum of **26** ($CDCl_3$)

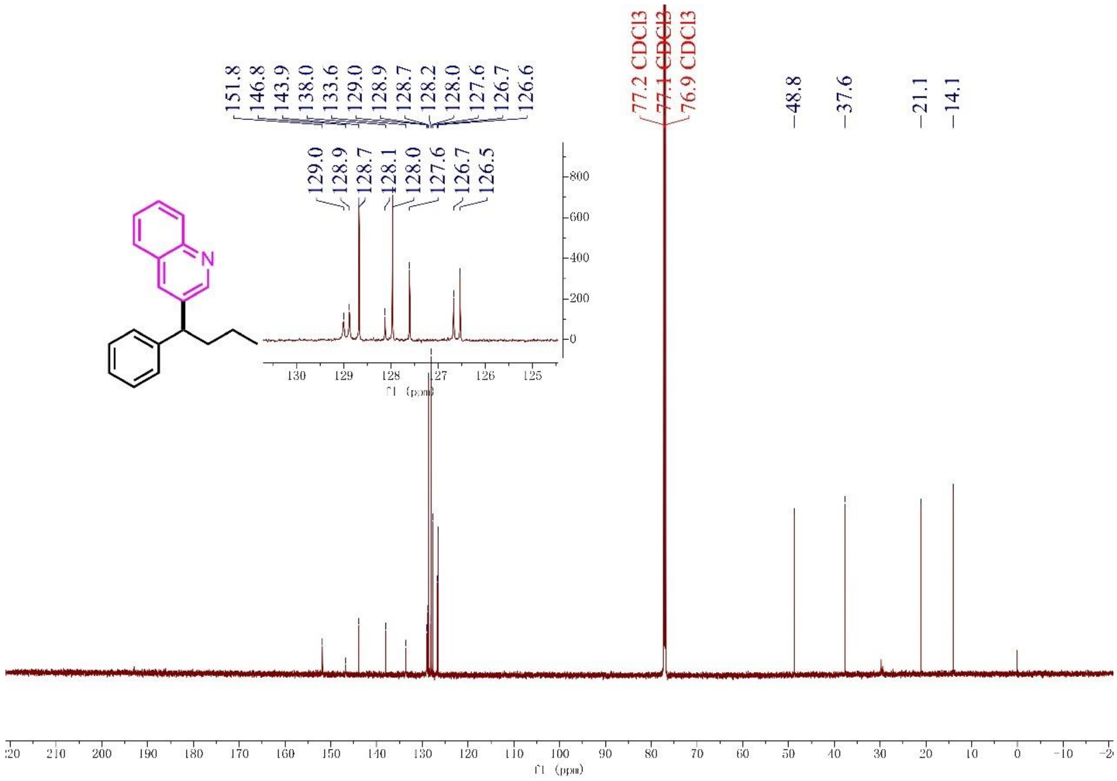

¹H NMR spectrum of **27** ($CDCl_3$)

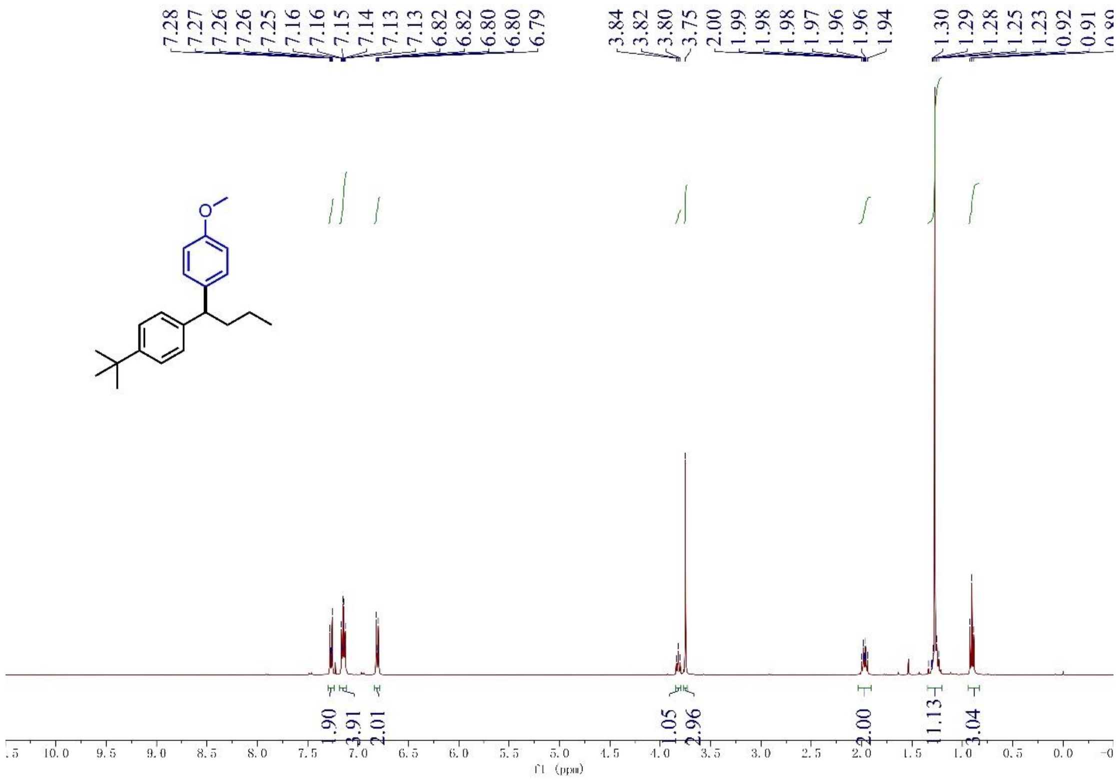

¹³C NMR spectrum of **27** ($CDCl_3$)

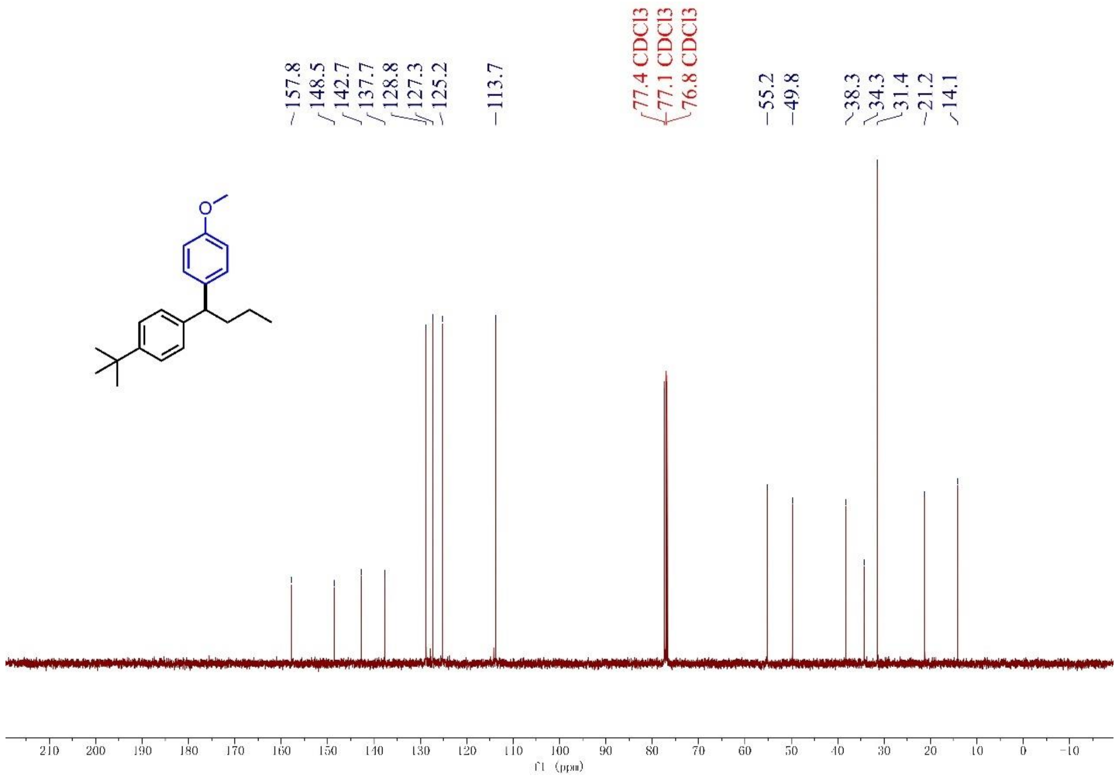

^{1}H NMR spectrum of **28** ($CDCl_3$)

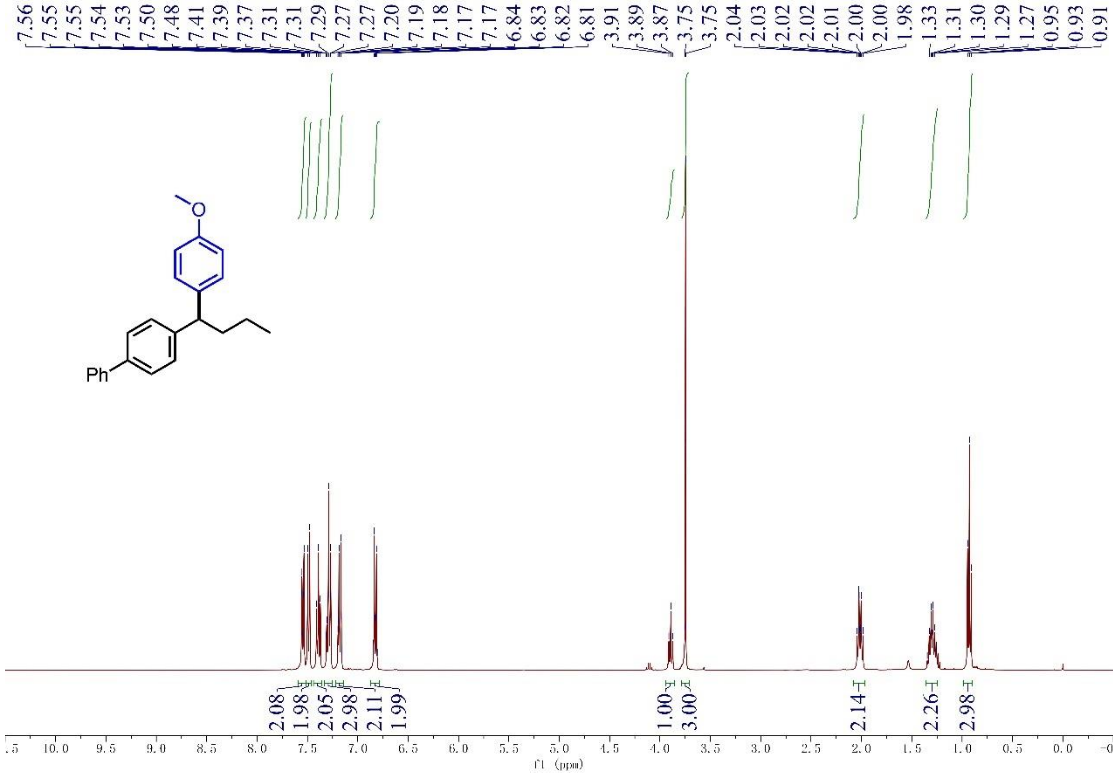

^{13}C NMR spectrum of **28** ($CDCl_3$)

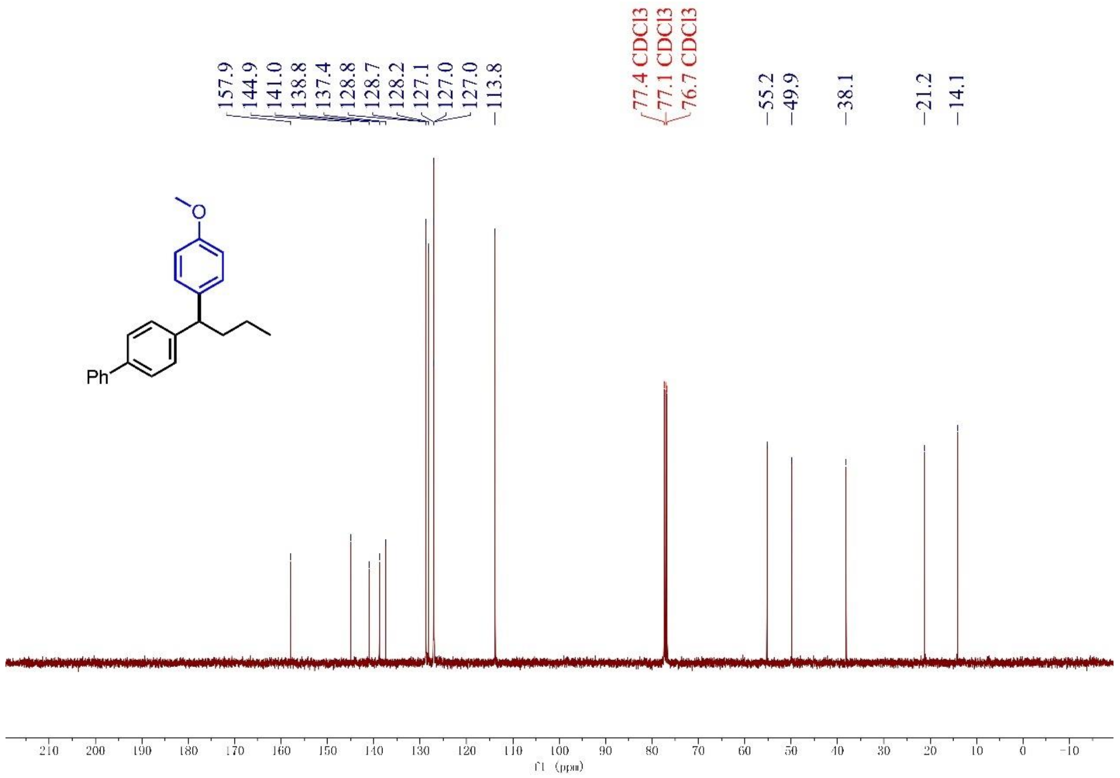

^{1}H NMR spectrum of **29** ($CDCl_3$)

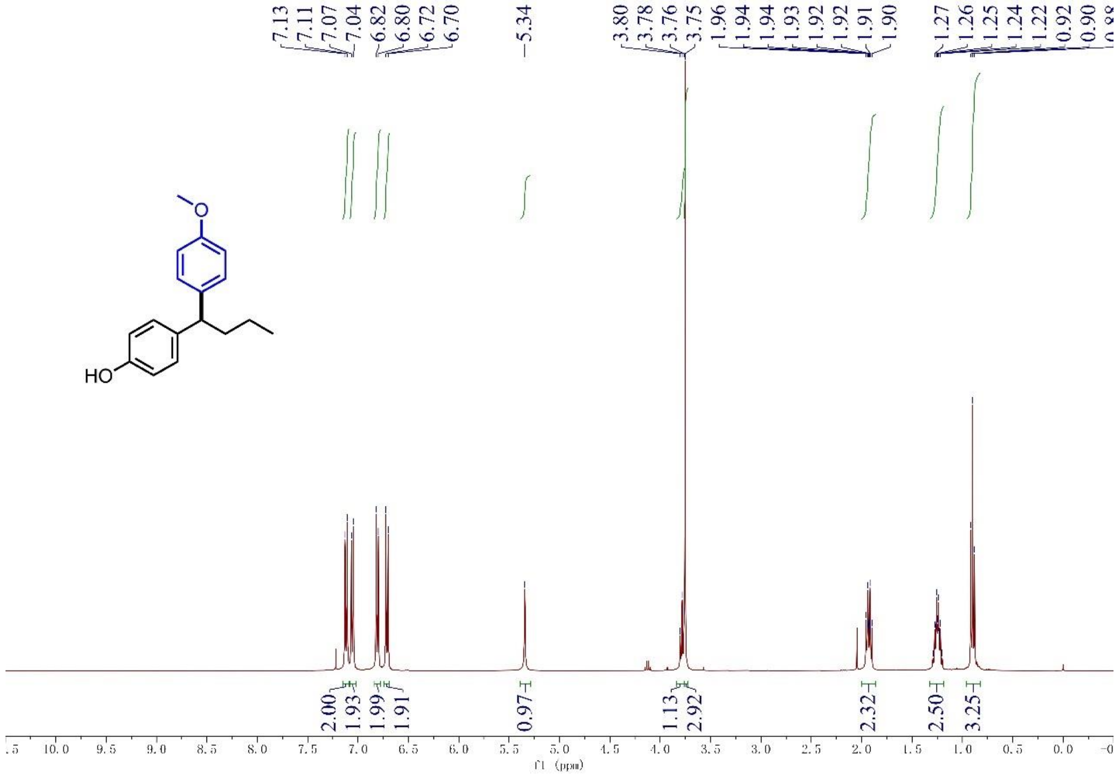

^{13}C NMR spectrum of **29** ($CDCl_3$)

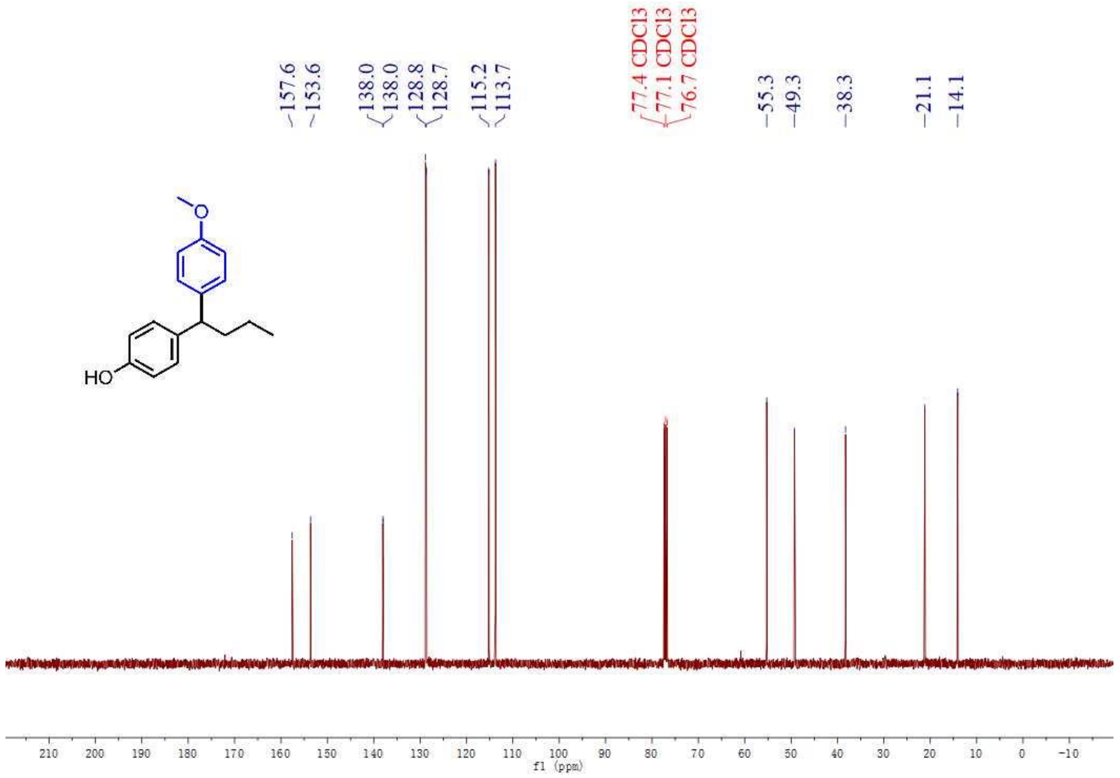

^{1}H NMR spectrum of **30** ($CDCl_3$)

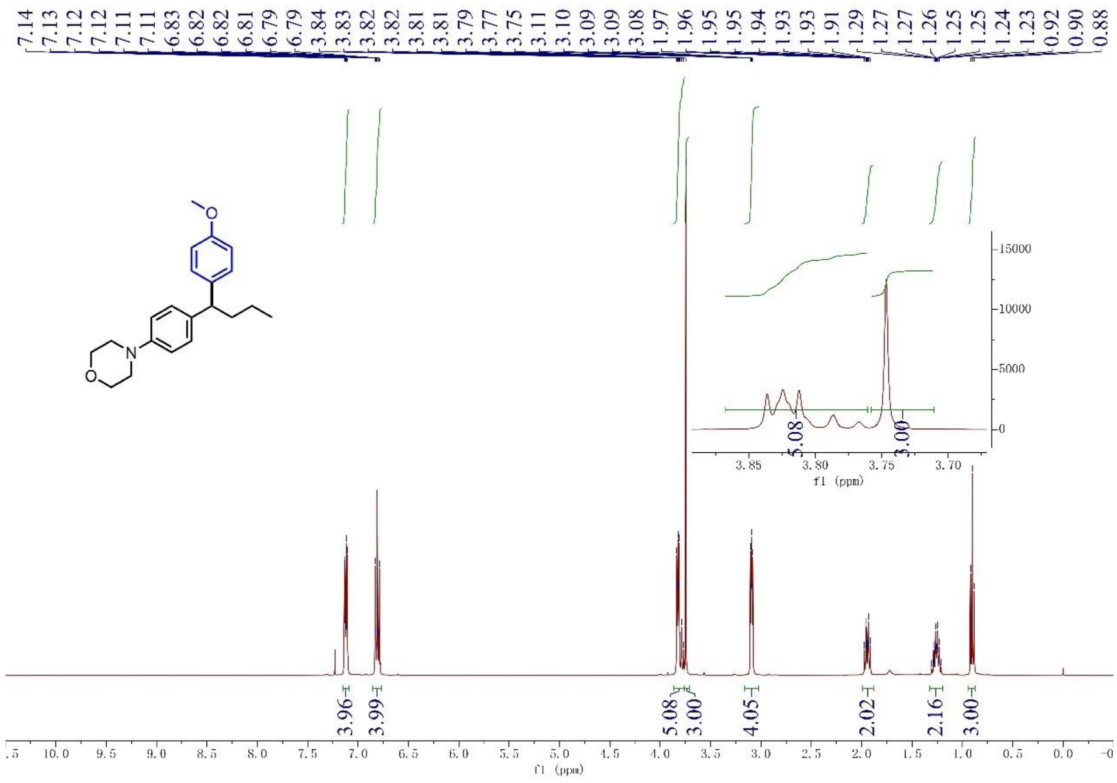

^{13}C NMR spectrum of **30** ($CDCl_3$)

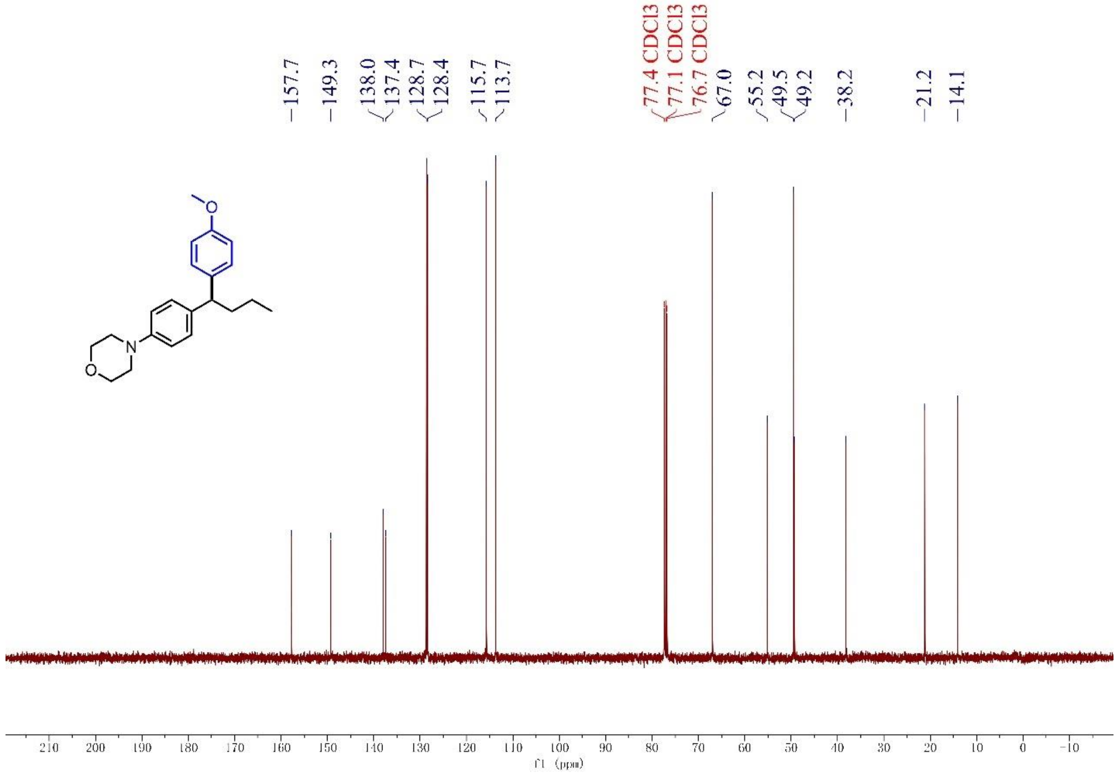

^{1}H NMR spectrum of **31** ($CDCl_3$)

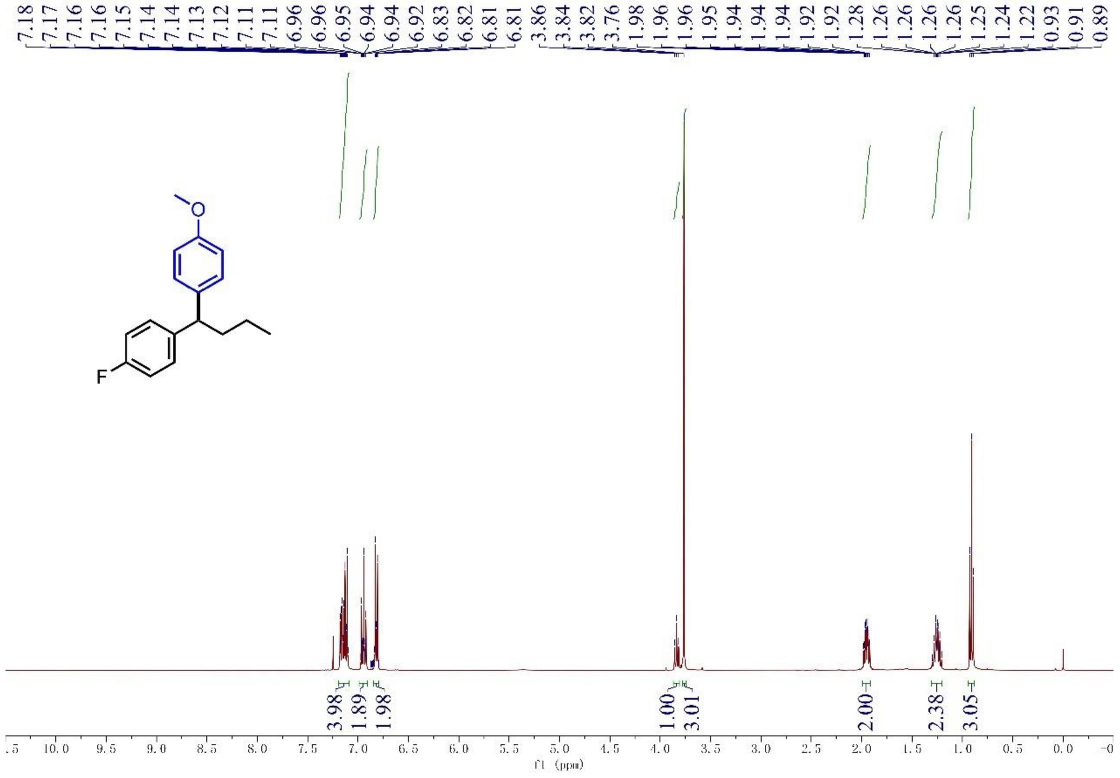

^{13}C NMR spectrum of **31** ($CDCl_3$)

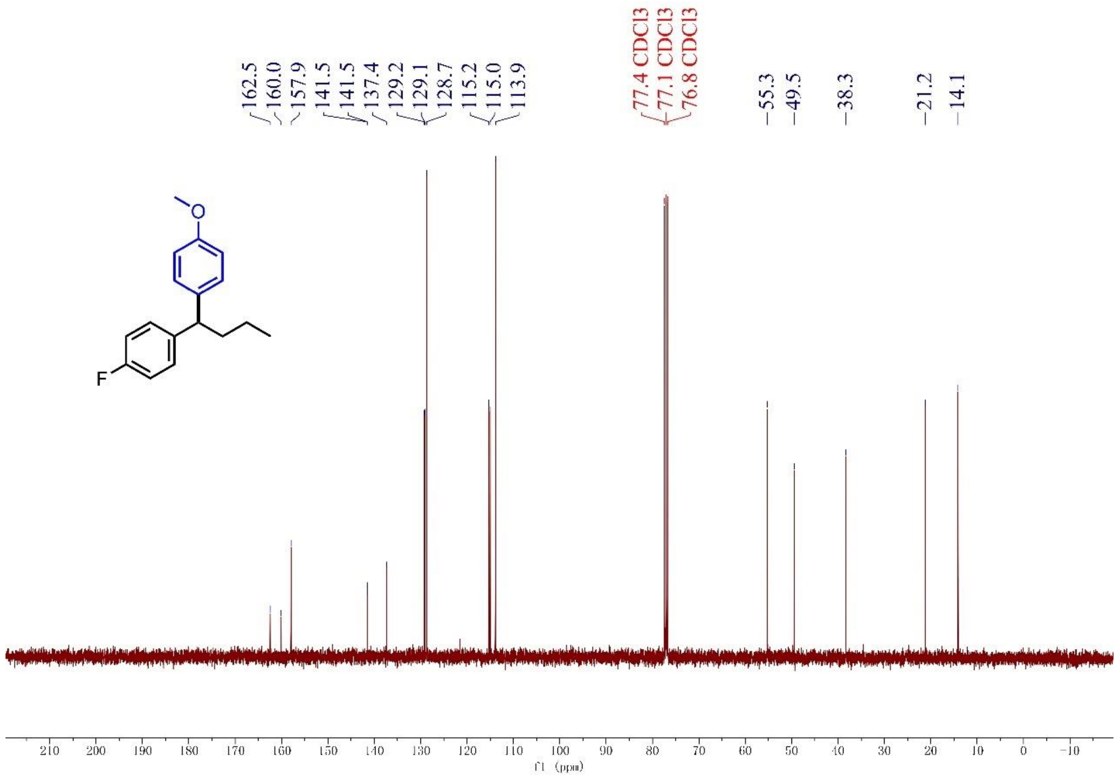

^{19}F NMR spectrum of **31** ($CDCl_3$)

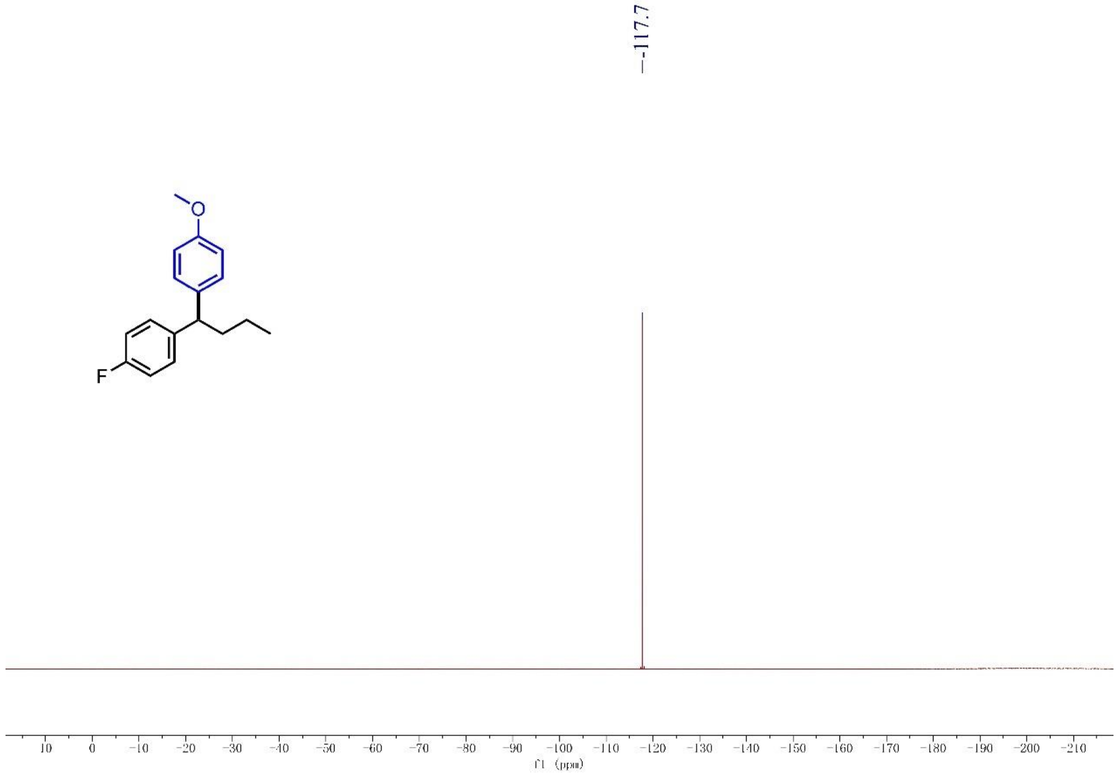

^{1}H NMR spectrum of **32** ($CDCl_3$)

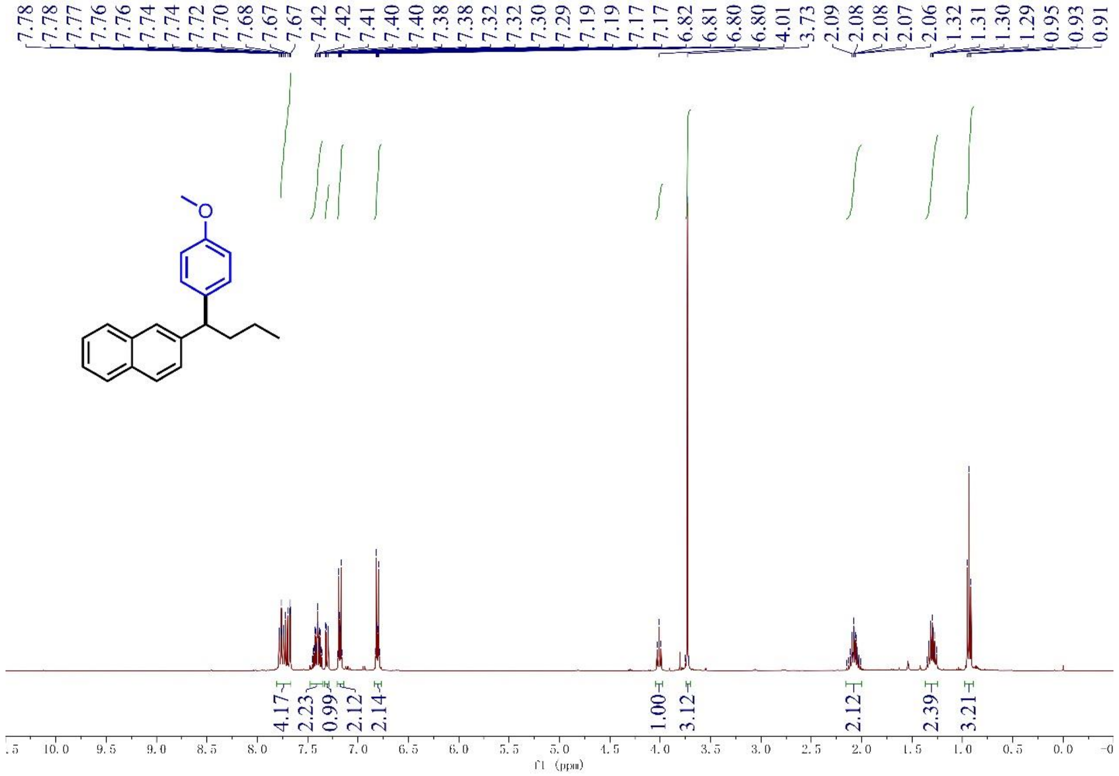

^{13}C NMR spectrum of **32** ($CDCl_3$)

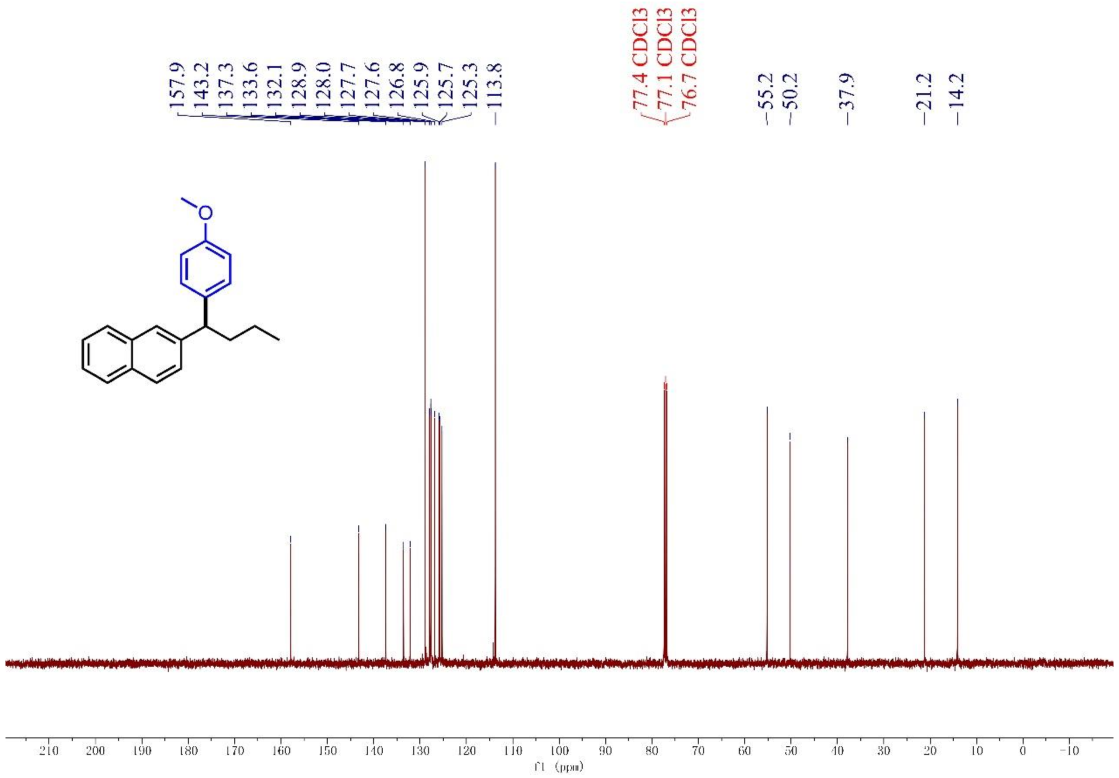

^{1}**H NMR** spectrum of **33** ($CDCl_3$)

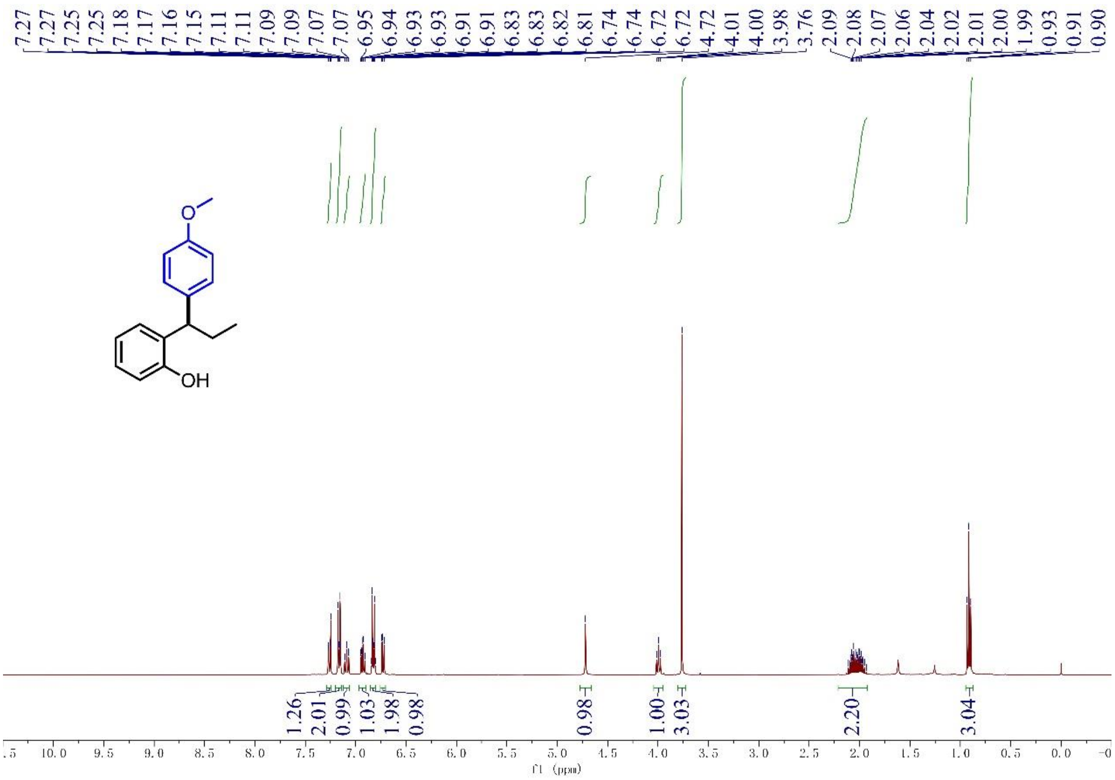

^{13}**C NMR** spectrum of **33** ($CDCl_3$)

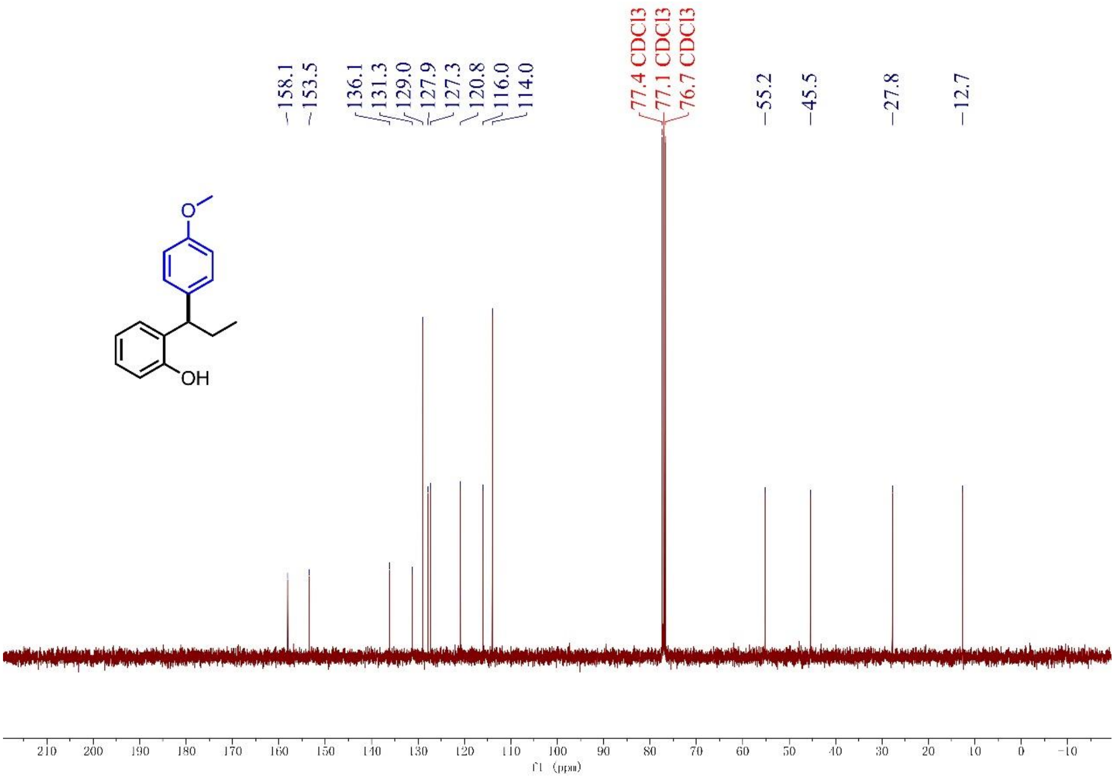

^{1}H NMR spectrum of **34** ($CDCl_3$)

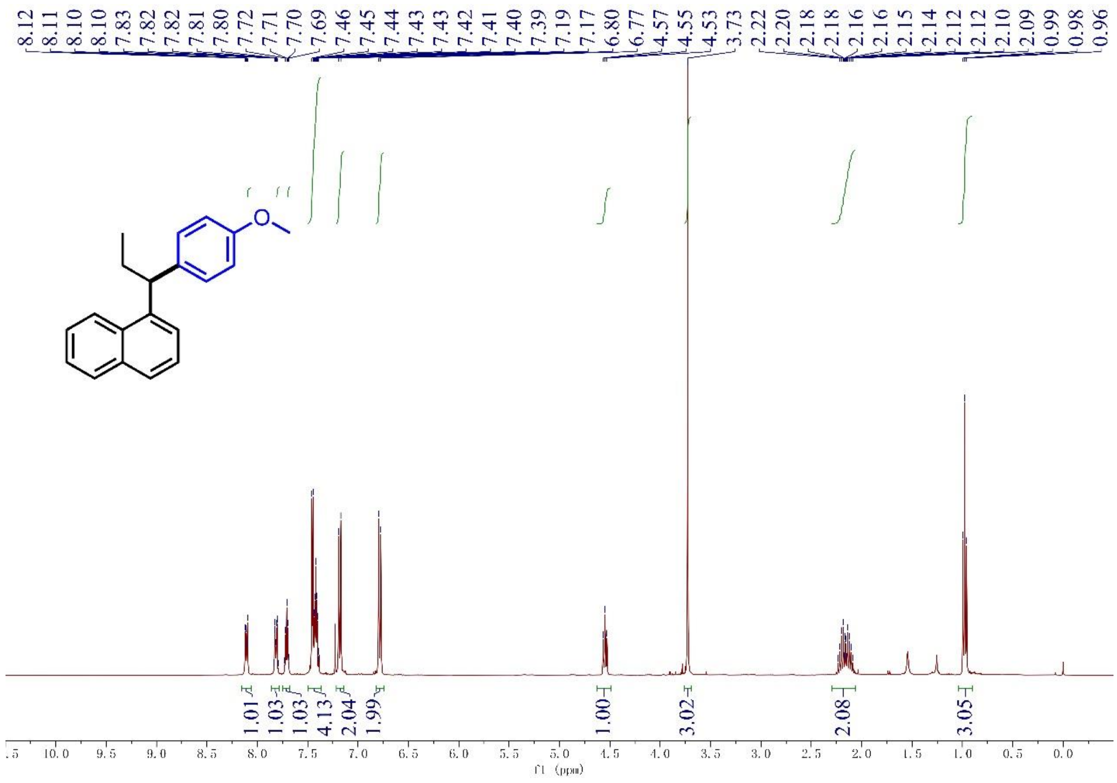

^{13}C NMR spectrum of **34** ($CDCl_3$)

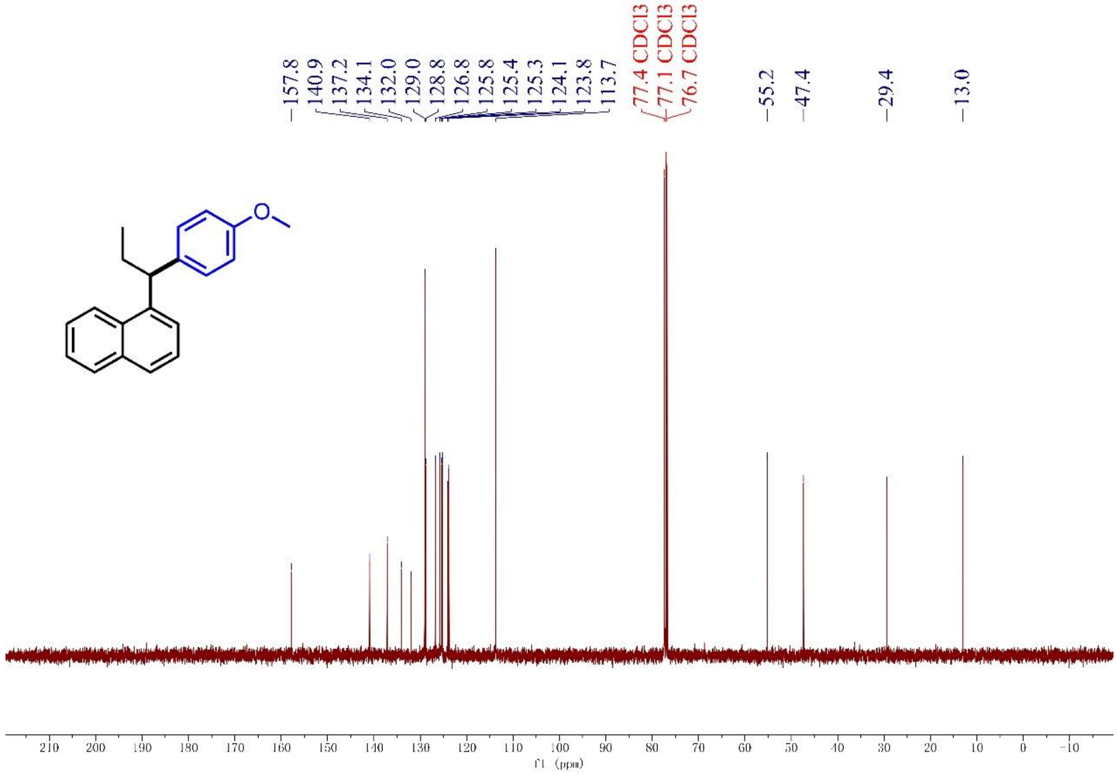

^{1}H NMR spectrum of **35** ($CDCl_3$)

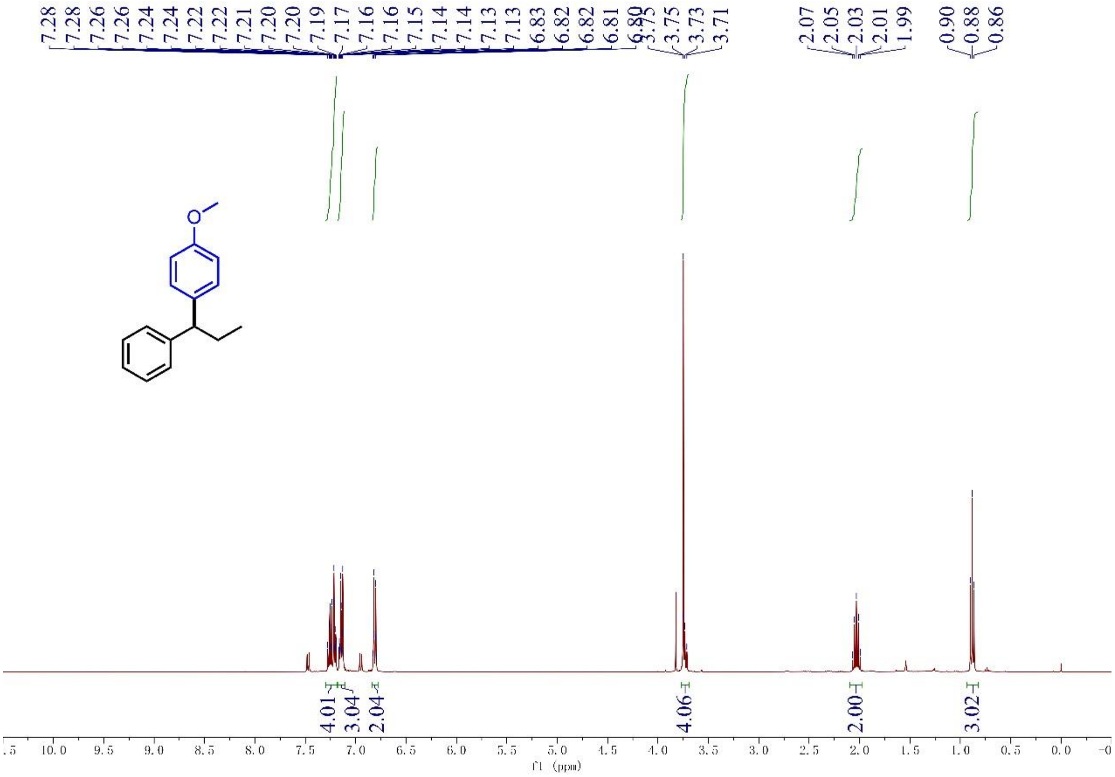

^{13}C NMR spectrum of **35** ($CDCl_3$)

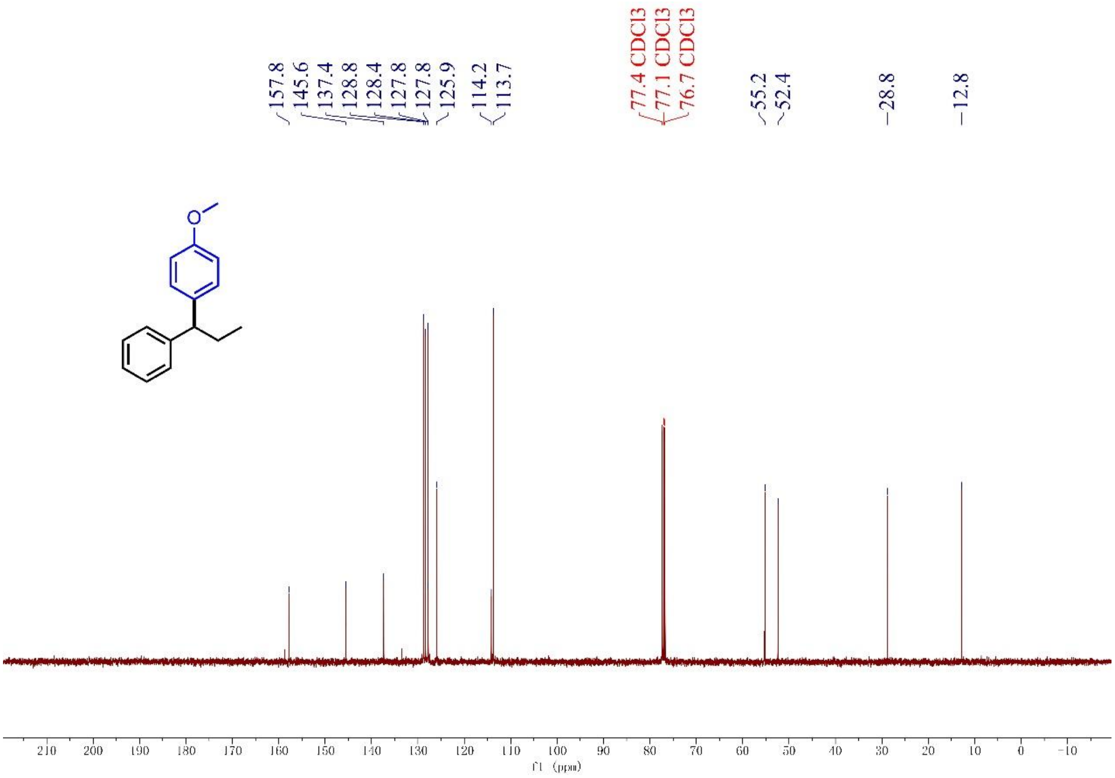

^{1}H NMR spectrum of **36** ($CDCl_3$)

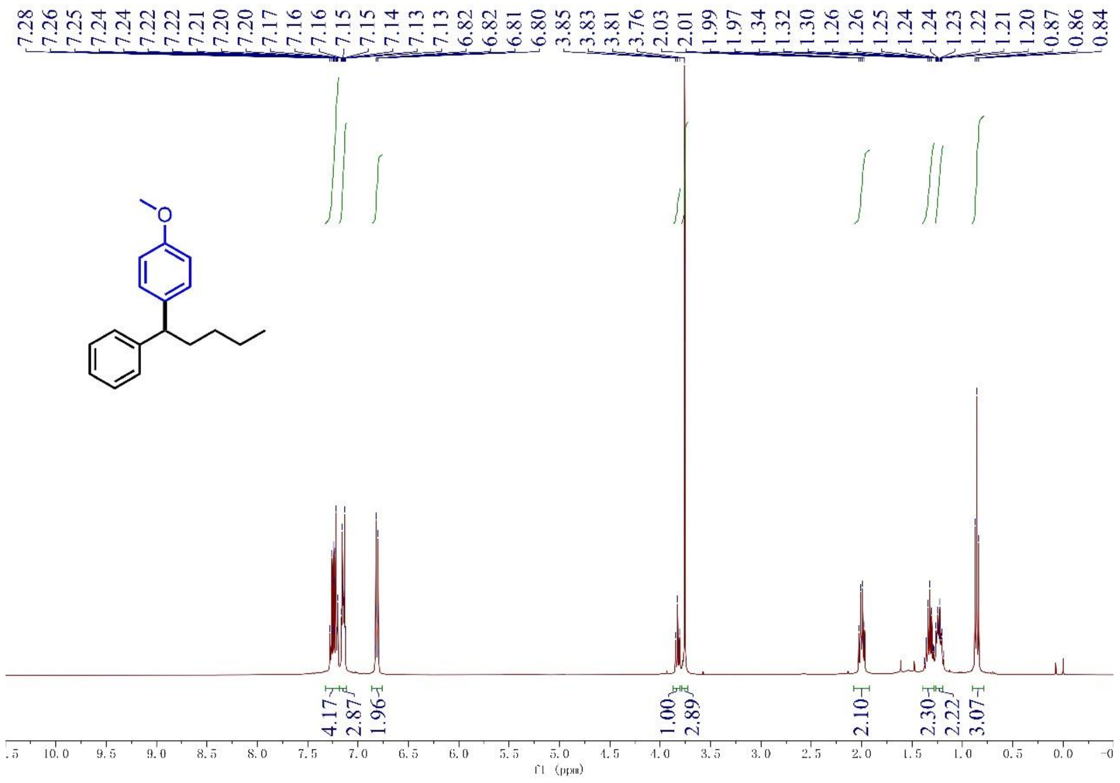

^{13}C NMR spectrum of **36** ($CDCl_3$)

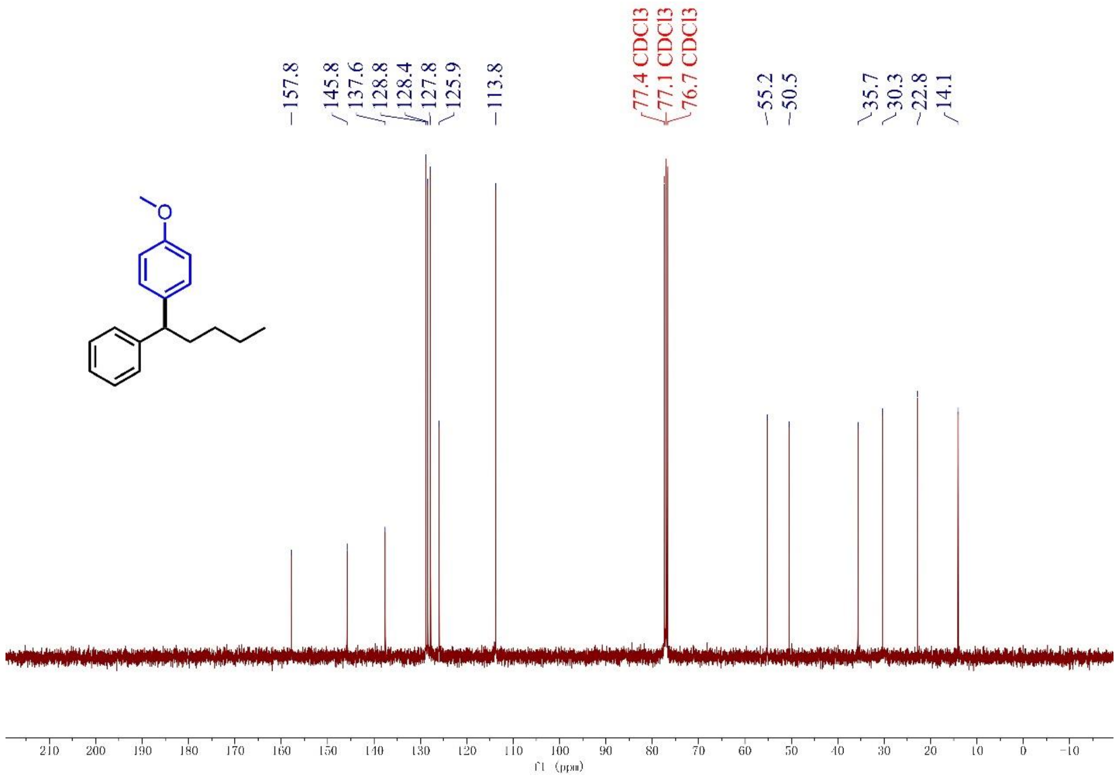

^{1}H NMR spectrum of **37** ($CDCl_3$)

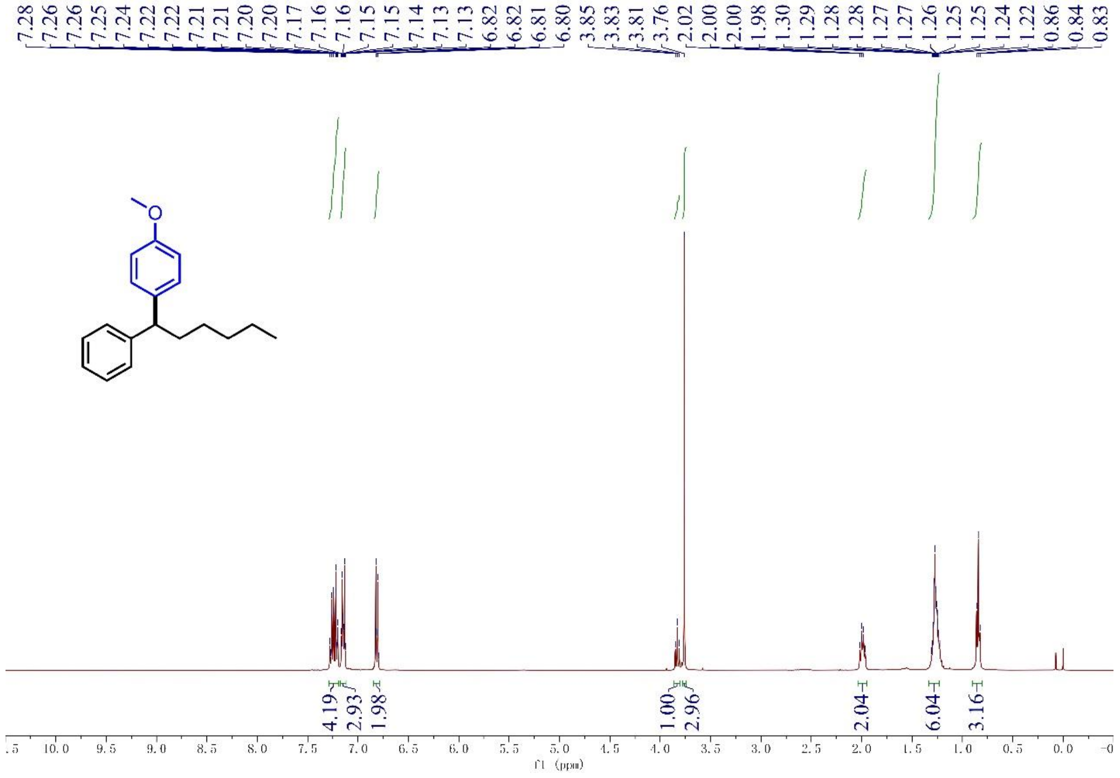

^{13}C NMR spectrum of **37** ($CDCl_3$)

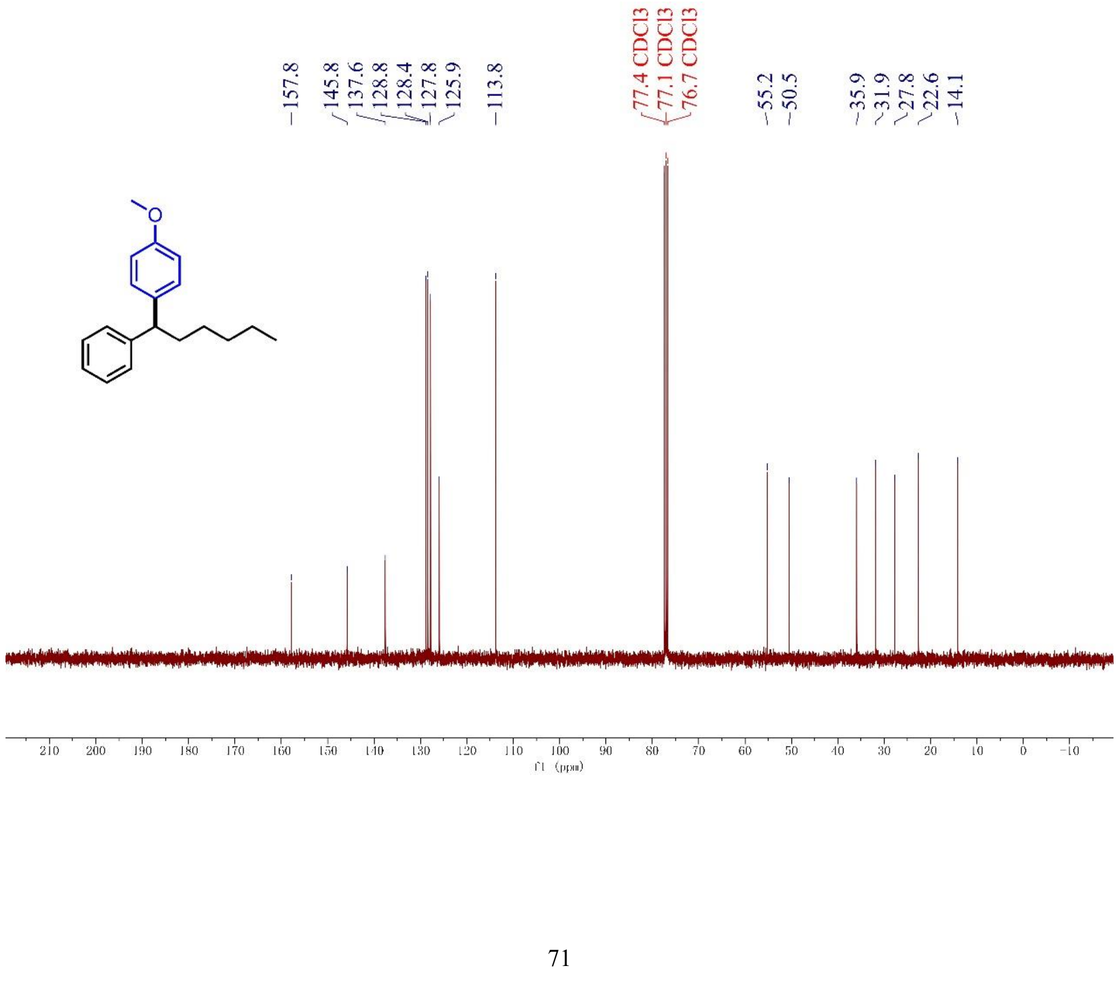

^{1}H NMR spectrum of **38** ($CDCl_3$)

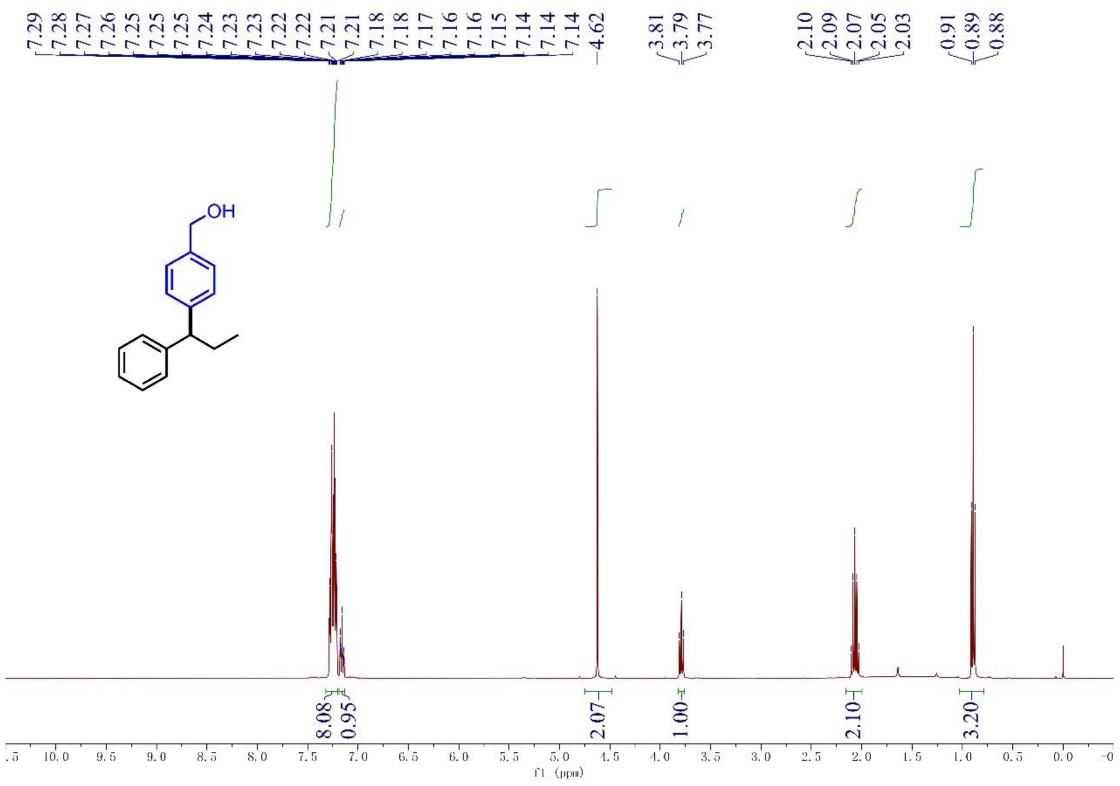

^{13}C NMR spectrum of **38** ($CDCl_3$)

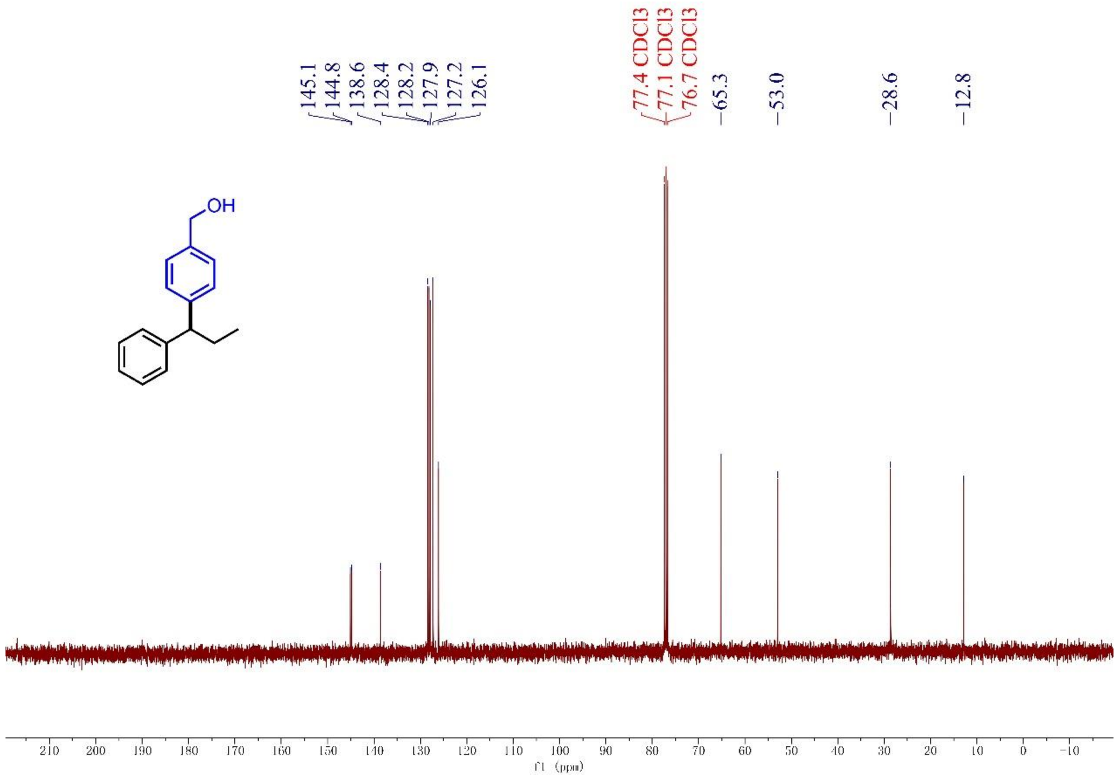

^{1}H NMR spectrum of **40** ($CDCl_3$)

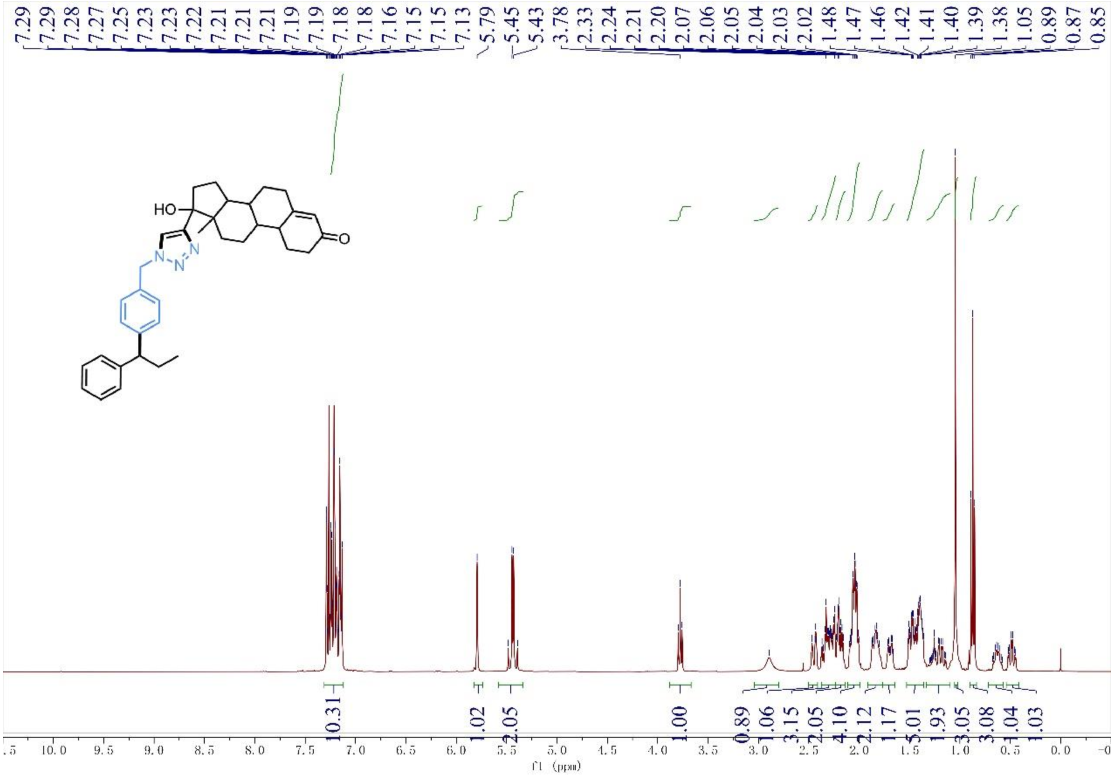

^{13}C NMR spectrum of **40** ($CDCl_3$)

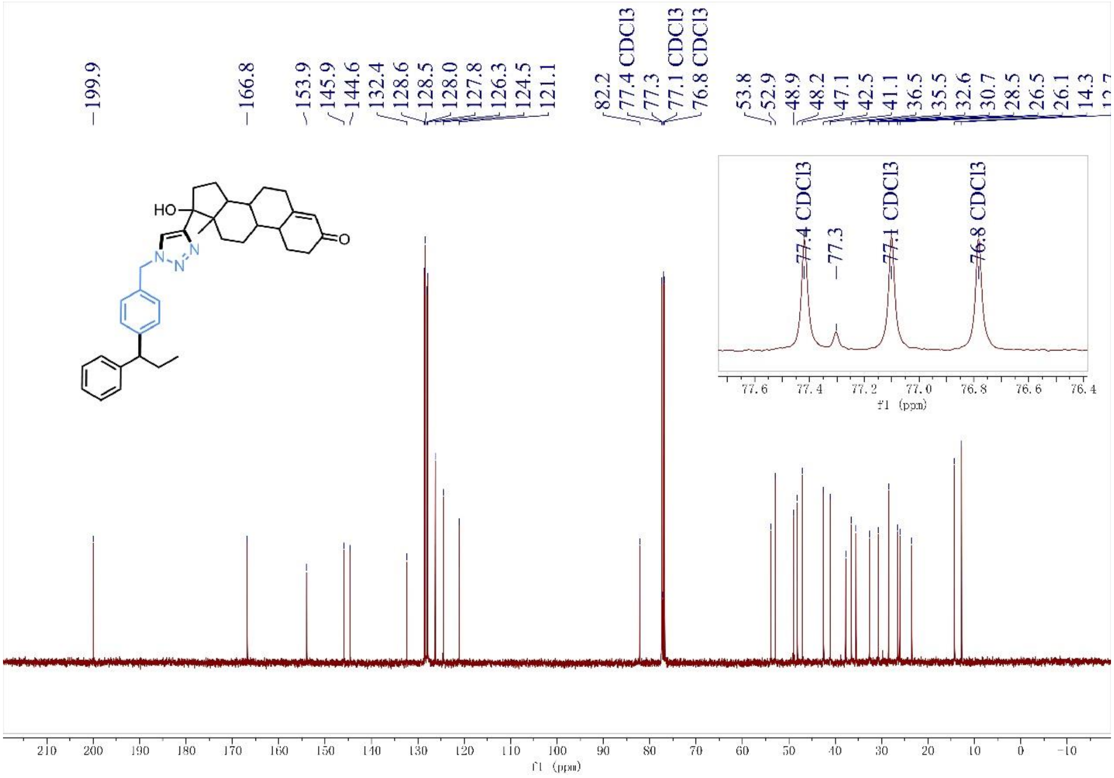

[1]**H NMR** spectrum of **41** ($CDCl_3$)

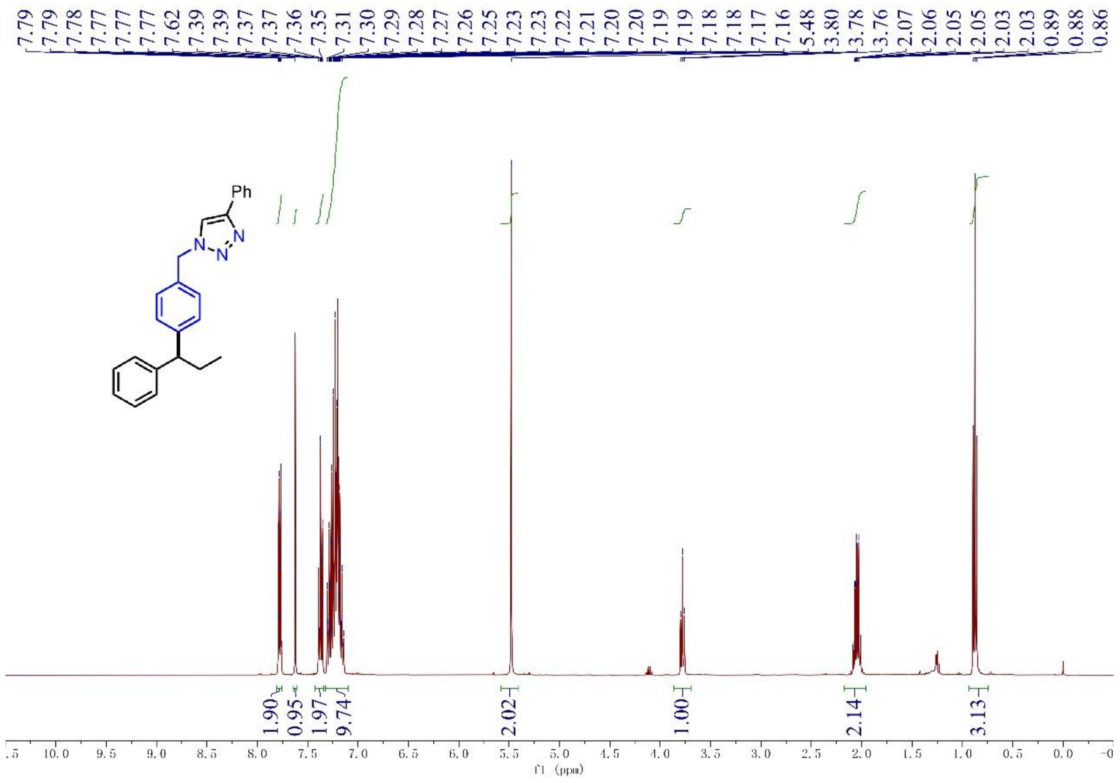

[13]**C NMR** spectrum of **41** ($CDCl_3$)

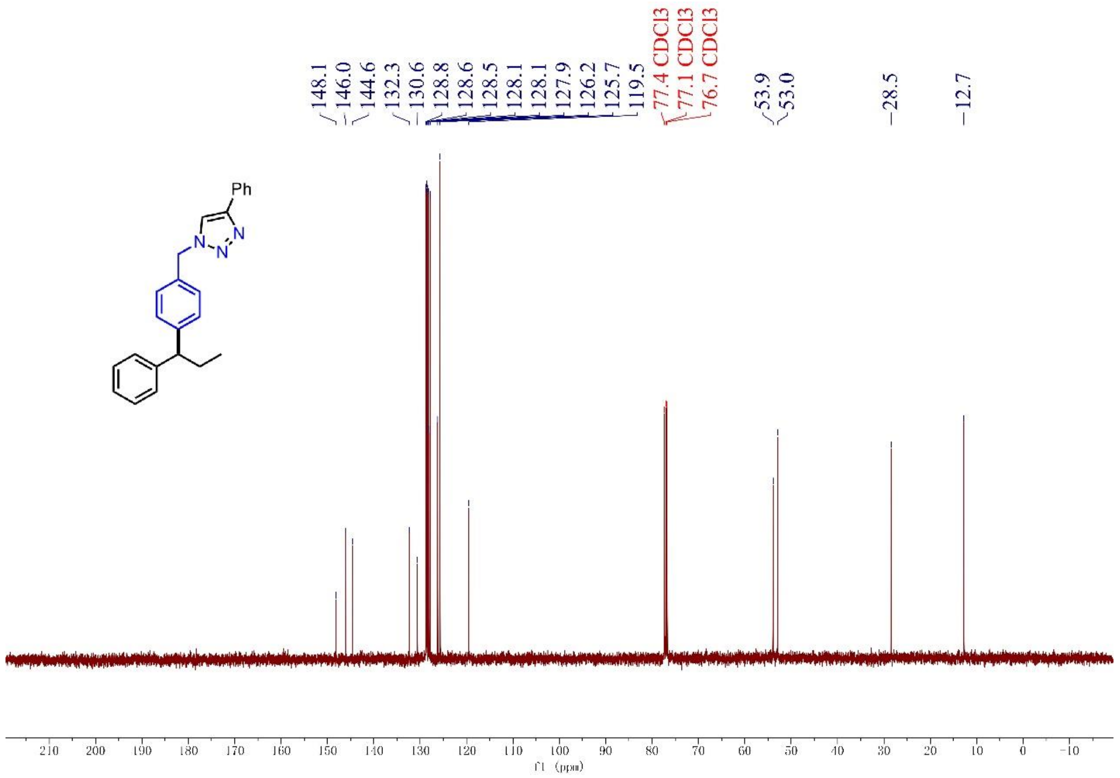

^{1}**H NMR** spectrum of **42** ($CDCl_3$)

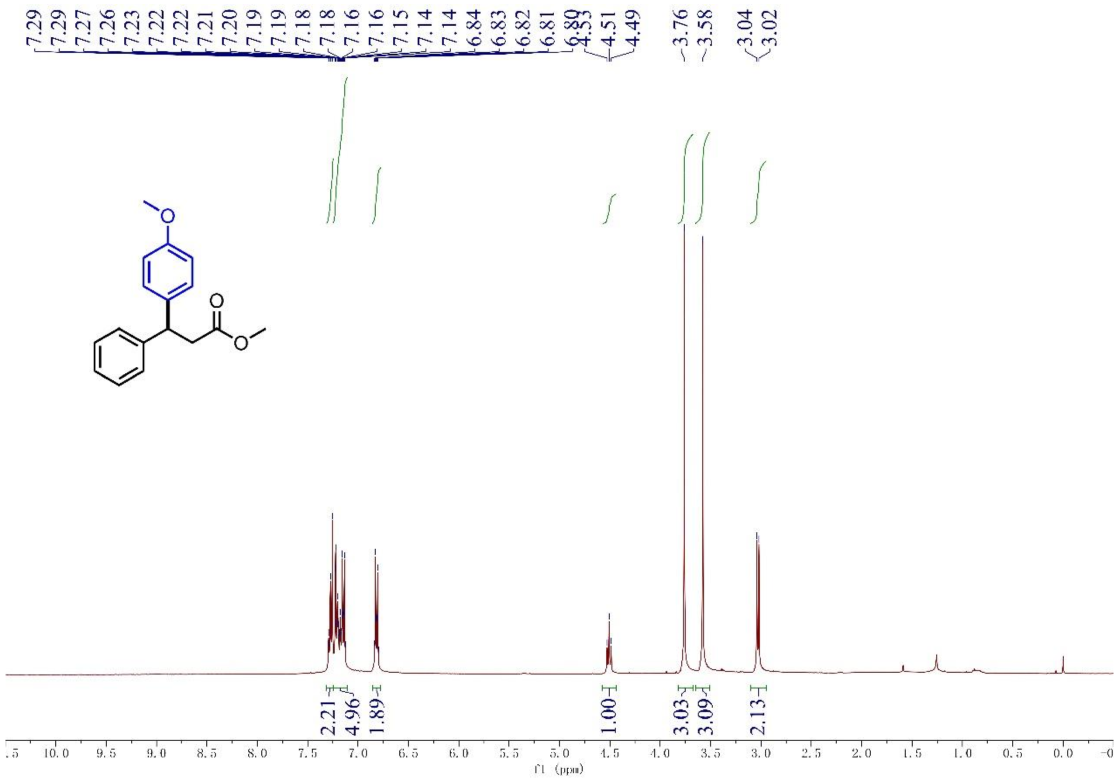

^{13}**C NMR** spectrum of **42** ($CDCl_3$)

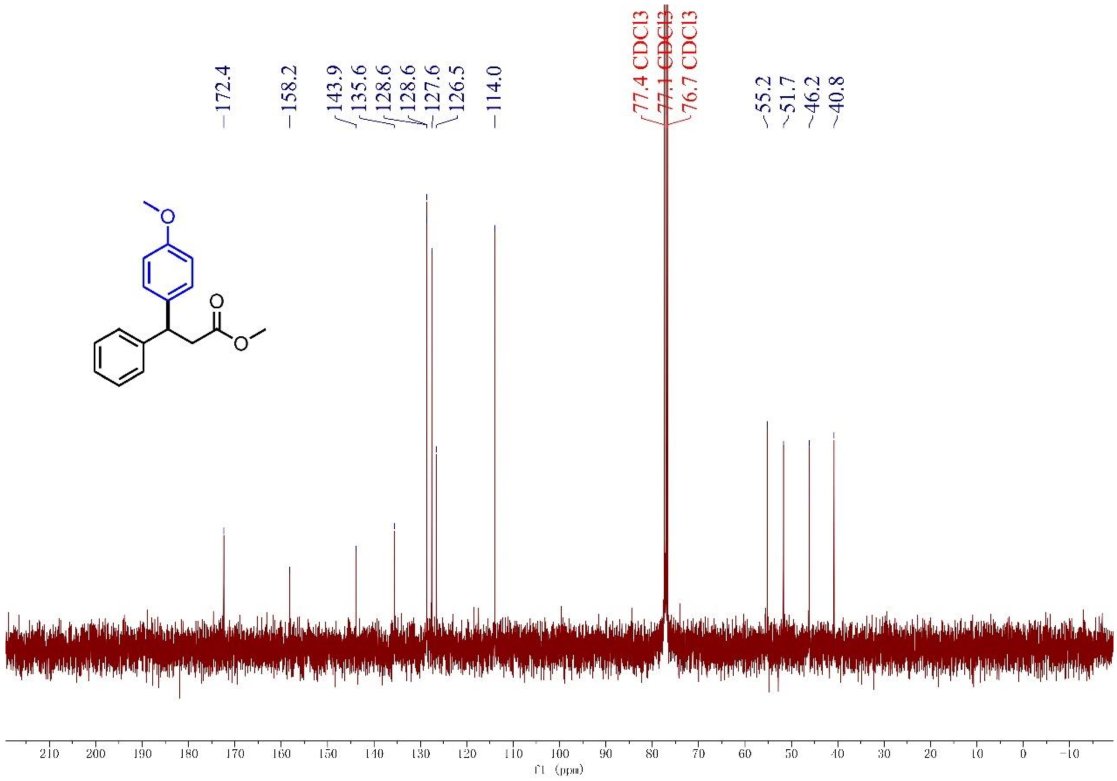

^{1}H NMR spectrum of **43** ($CDCl_3$)

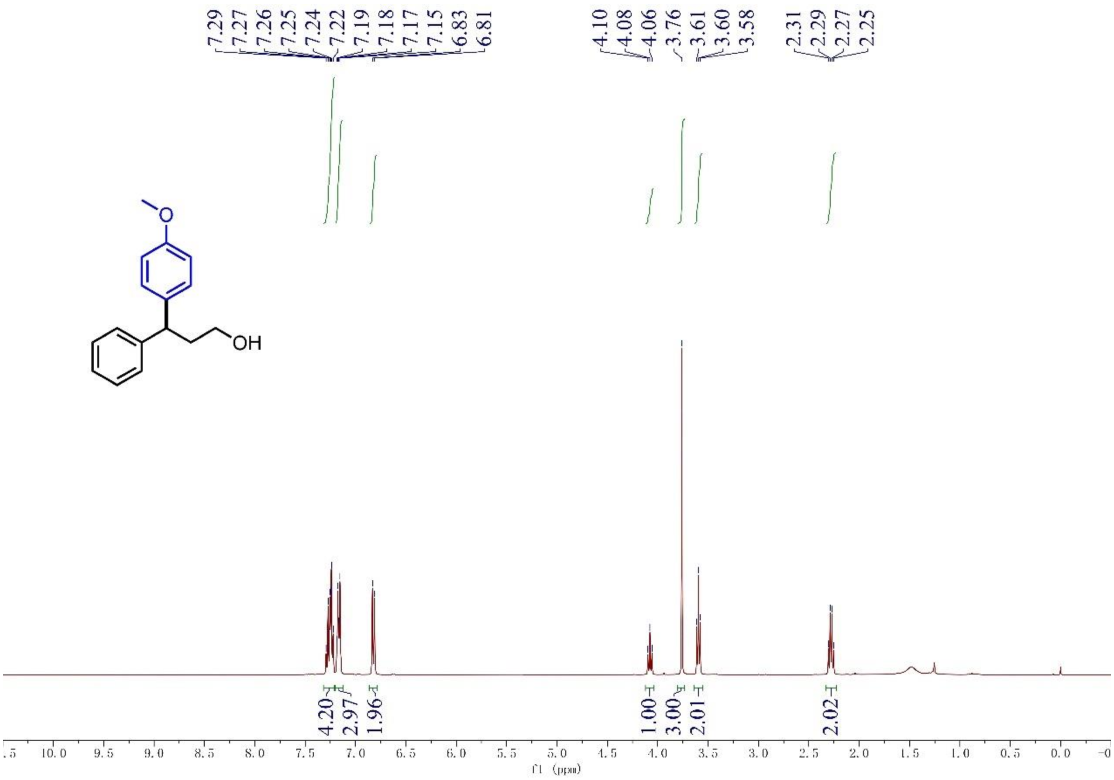

^{13}C NMR spectrum of **43** ($CDCl_3$)

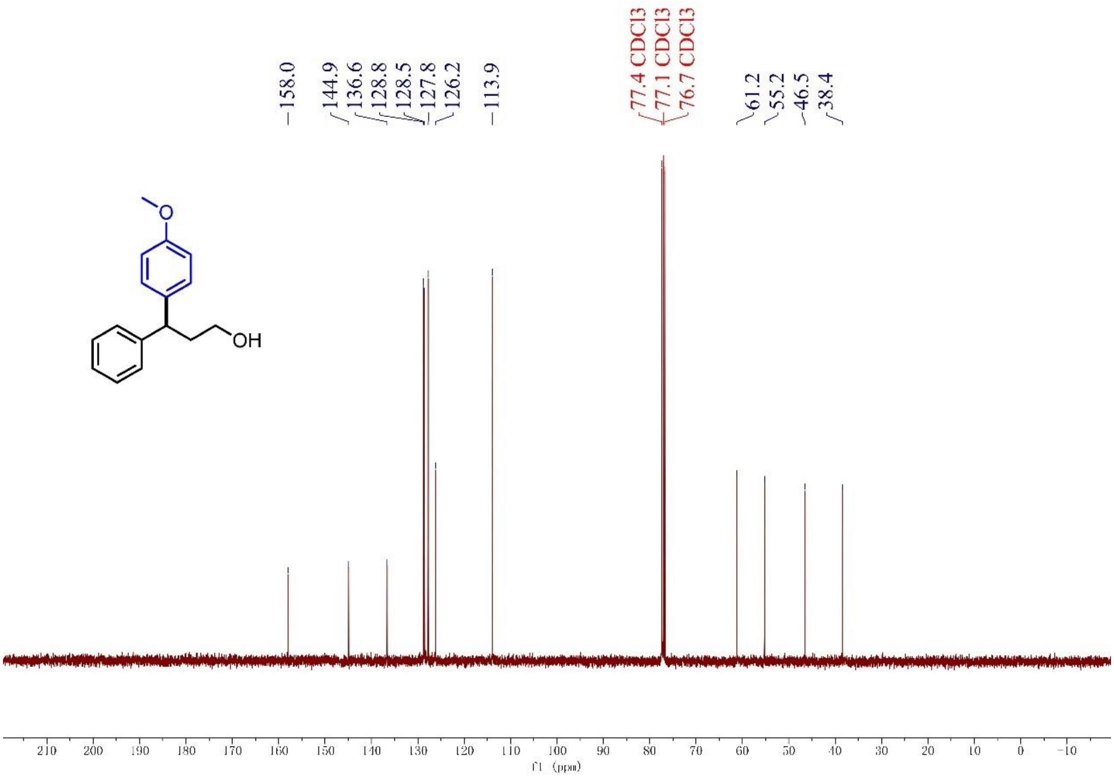

^{1}H NMR spectrum of **43** ($CDCl_3$)

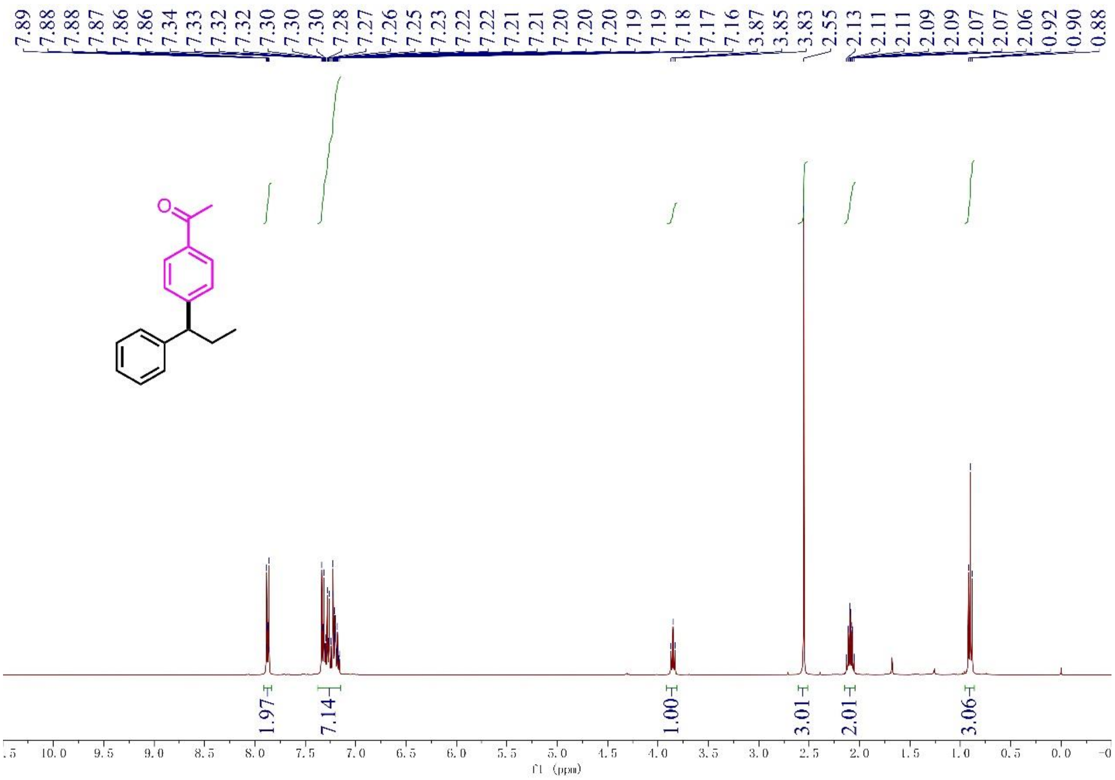

^{13}C NMR spectrum of **43** ($CDCl_3$)

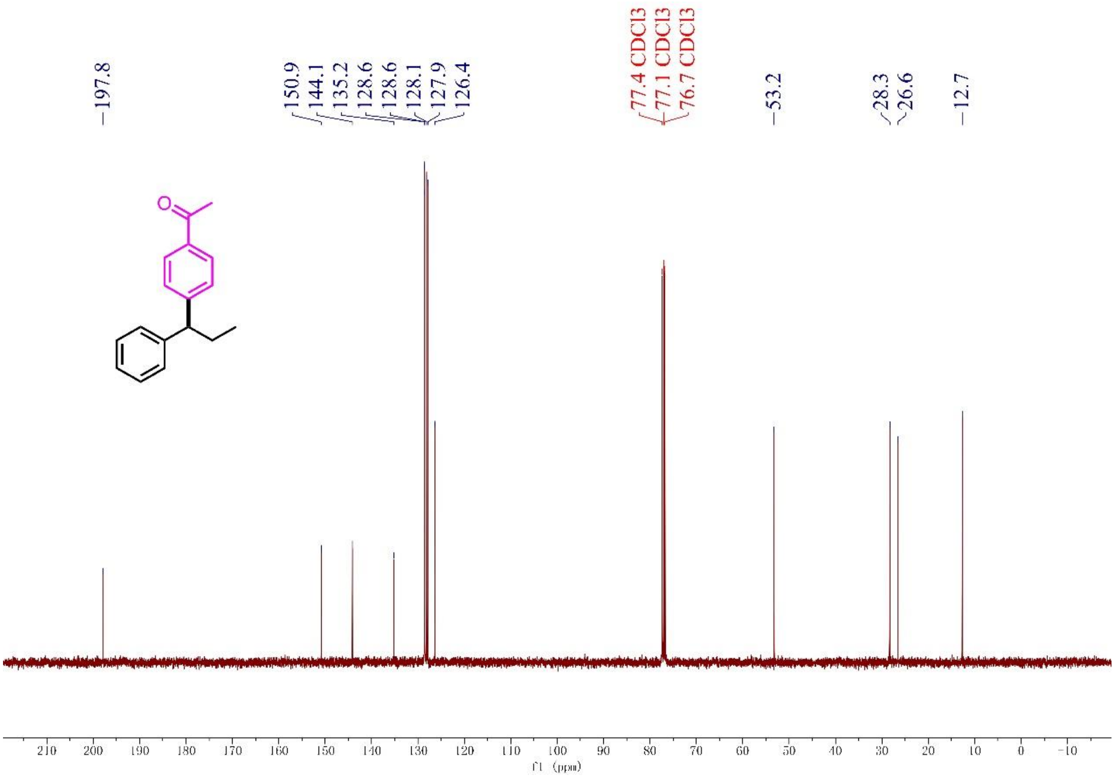

^{1}H NMR spectrum of **46** ($CDCl_3$)

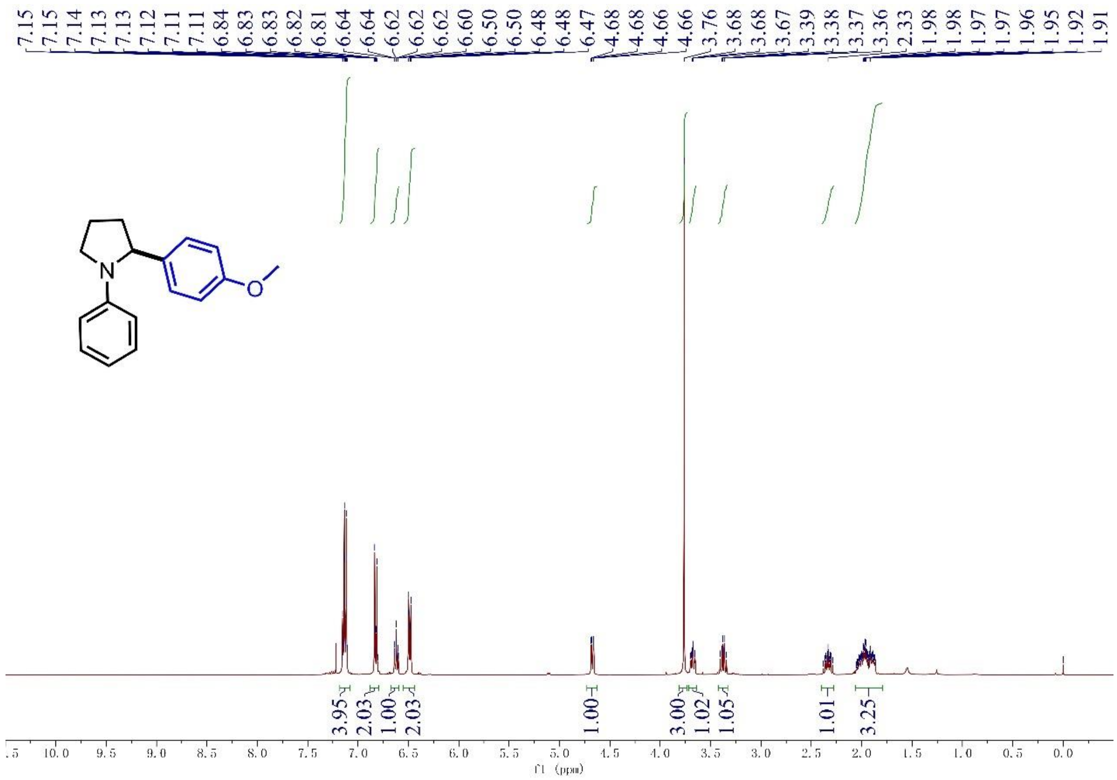

^{13}C NMR spectrum of **46** ($CDCl_3$)

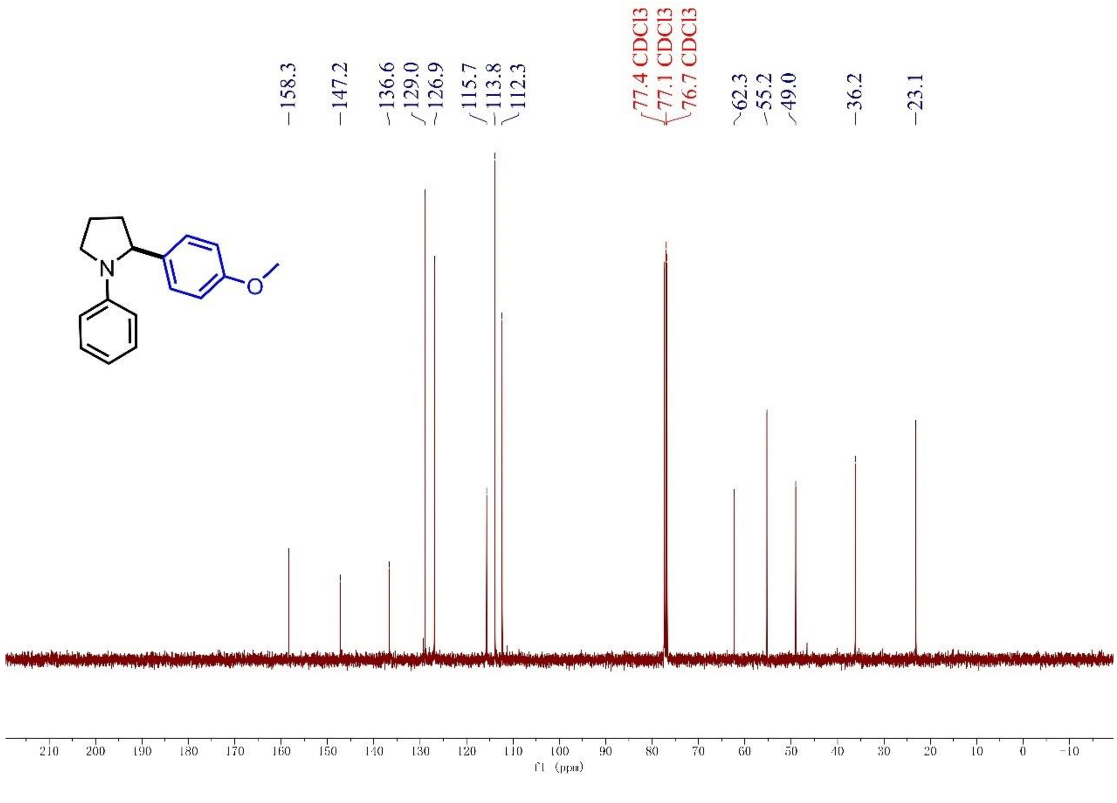

^{1}H NMR spectrum of **46** ($CDCl_3$)

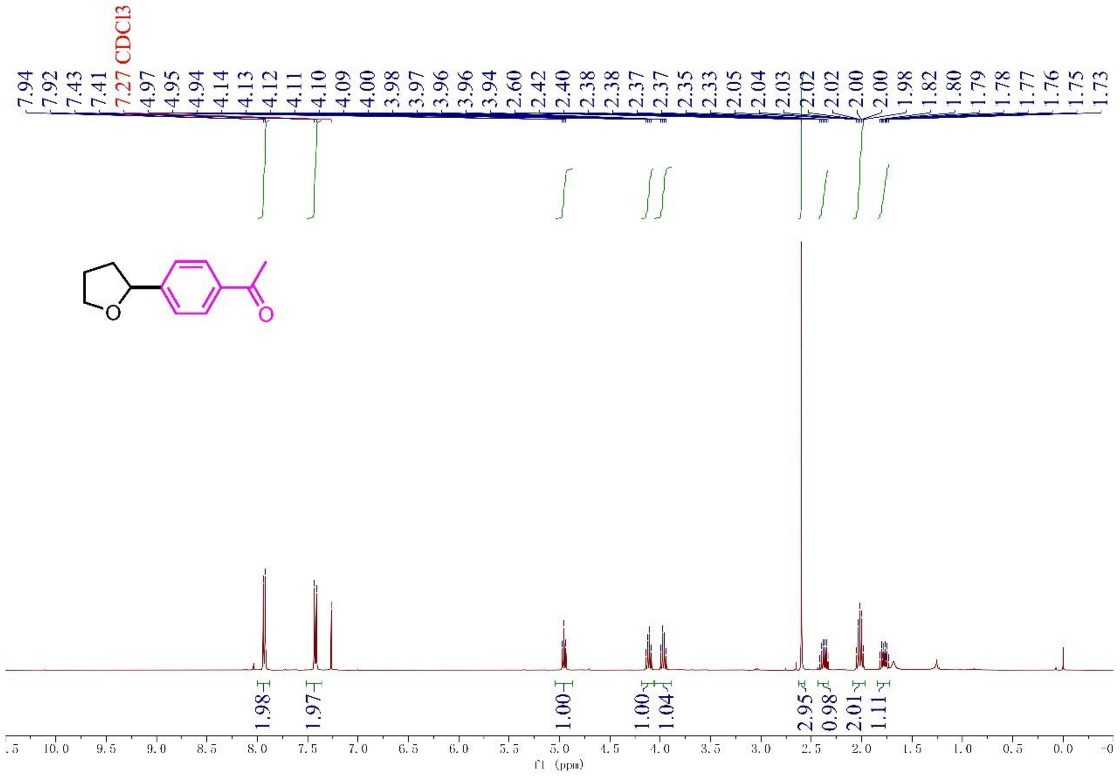

^{13}C NMR spectrum of **47** ($CDCl_3$)

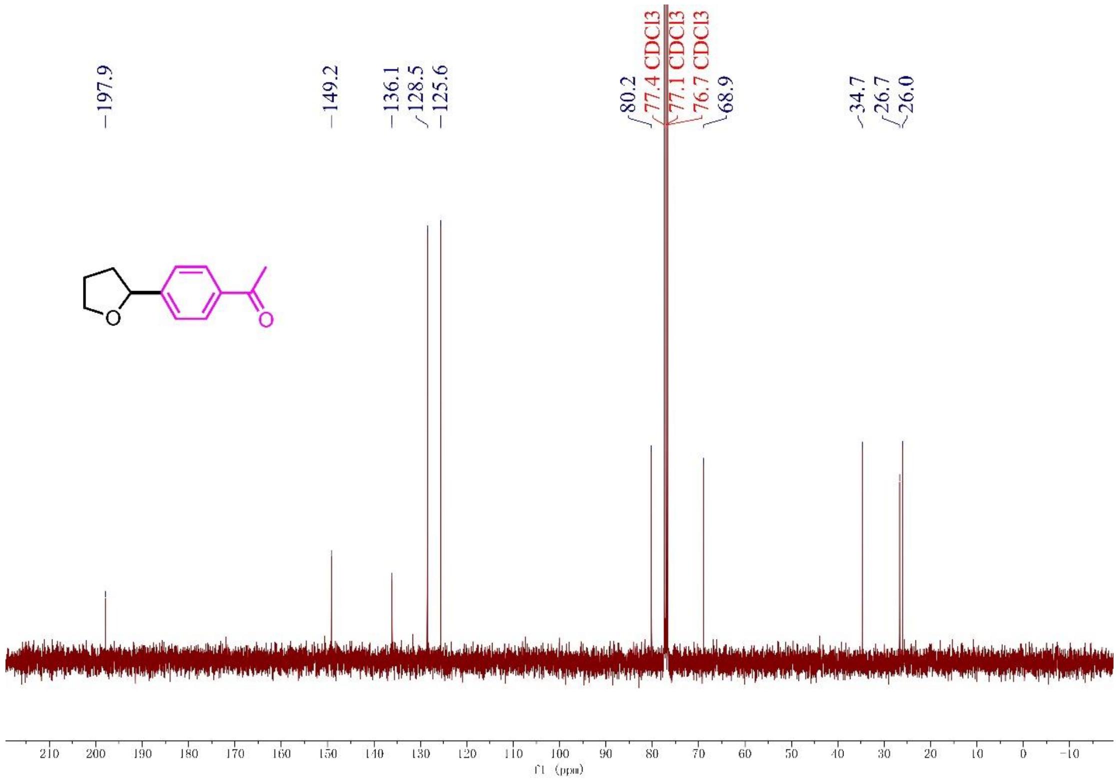

Supplement: Supplementary file 1 — Supporting Information [file CSSC-18-e202402196-s001.pdf]
